# Comparing interventions for early psychosis: a systematic review and component network meta-analysis

**DOI:** 10.1016/j.eclinm.2024.102537

**Published:** 2024-03-14

**Authors:** Ryan Williams, Edoardo G. Ostinelli, Joel Agorinya, Amedeo Minichino, Franco De Crescenzo, Daniel Maughan, Stephen Puntis, Charlotte Cliffe, Ayse Kurtulmus, Belinda R. Lennox, Andrea Cipriani

**Affiliations:** a Department of Brain Sciences, Imperial College London, London, UK; b South London & Maudsley NHS Foundation Trust, Maudsley Hospital, London, UK; c Department of Psychiatry, University of Oxford, Oxford, UK; d Oxford Precision Psychiatry Lab, NIHR Oxford Health Biomedical Research Centre, Oxford, UK; e Oxford Health NHS Foundation Trust, Warneford Hospital, Oxford, UK; f Accra Psychiatric Hospital, Accra, Ghana; g Biomedical Research Centre, Kings College London, London, UK; h Surrey and Borders Partnership NHS Foundation Trust, UK; i Department of Psychiatry, Istanbul Medeniyet University, Istanbul, Turkey

**Keywords:** Early Intervention in Psychosis, Mental health services, Schizophrenia, First episode psychosis, Component network meta-analysis

## Abstract

**Background:**

‘Early Intervention in Psychosis’ (EIP) services have been associated with improved outcomes for early psychosis. However, these services are heterogeneous and many provide different components of treatment. The impact of this variation on the sustained treatment effects is unknown.

**Methods:**

We performed a systematic review and component network meta-analysis (cNMA) of randomised controlled trials (RCTs) that compared specialised intervention services for early psychosis. We searched CENTRAL (published and unpublished), EMBASE, MEDLINE, CINAHL, PsycINFO and Web of Science from inception to February 2023. Primary outcomes were negative and positive psychotic symptoms at 3-month and 1-year follow-up and treatment dropouts. Secondary outcomes were depressive symptoms and social functioning at 1-year follow-up. We registered a protocol for our study in PROSPERO (CRD42017057420).

**Findings:**

We identified 37 RCTs including 4599 participants. Participants’ mean age was 25.8 years (SD 6.0) and 64.0% were men. We found evidence that psychological interventions (this component grouped all psychological treatment intended to treat, or ameliorate the consequences of, psychotic symptoms) are beneficial for reducing negative symptoms (iSMD −0.24, 95% CI −0.44 to −0.05, p = 0.014) at 3-month follow-up and may be associated with clinically relevant benefits in improving social functioning scores at 1-year follow-up (iSMD −0.52, 95% CI −1.05 to 0.01, p = 0.052). The addition of case management has a beneficial effect on reducing negative symptoms (iSMD −1.17, 95% CI −2.24 to −0.11, p = 0.030) and positive symptoms (iSMD −1.05, 95% CI −2.02 to −0.08, p = 0.033) at 1-year follow-up. Pharmacotherapy was present in all trial arms, meaning it was not possible to examine the specific effects of this component.

**Interpretation:**

Our findings suggest psychological interventions and case management in addition to pharmacotherapy as the core components of services for early psychosis to achieve sustained clinical benefits. Our conclusions are limited by the small number of studies and sparsely connected networks.

**Funding:**

10.13039/501100000272National Institute for Health and Care Research.

**Research in context**

**Evidence before this study**

Early Intervention in Psychosis (EIP) services deliver complex interventions, with components including pharmacotherapy, individual psychological therapy, family interventions, and social interventions. EIP services as a whole package have also been shown to be effective in improving outcomes. However how these components influence overall outcomes is not known. We searched for studies that examined the comparative contributions made by EIP components to the overall outcome (‘dismantling’ studies or component network meta-analyses). We searched PubMed on 16/11/2023, with no restrictions on language or publication date, using the following search strategy: (“Mental Disorders” [Mesh] “Psychotic Disorders” [Mesh] dismantl∗) OR (“Mental Disorders” [Mesh] “Psychotic Disorders” [Mesh] component network meta-analysis). This search returned two network meta-analyses, one comparing pharmacotherapies for acute agitation associated with psychotic disorders and another comparing lifestyle interventions for weight outcomes associated with psychotic disorders. No meta-research studies have examined the comparative efficacy of components of care delivered by EIP services.

**Added value of this study**

To our knowledge, this is the first meta-research study examining the component-specific performance of EIP services. We found suggestive evidence that specific components (psychological interventions and case management) may have beneficial effects compared to pharmacotherapy alone for some aspects of early psychosis—positive and negative psychotic symptoms and social functioning.

**Implications of all the available evidence**

Our findings support current service models for EIP services, although it calls for standardisation of their components. Case management and, to some extent, psychological interventions alongside pharmacotherapy may constitute the core combination of treatment to achieve sustained or prolonged benefits.

## Introduction

Psychotic disorders are distressing and disabling conditions with severe effects on global functioning.^1^^,^^2^ The incidence of a ‘first episode of psychosis’ has been estimated at around 50 in 100,000 people each year,^3^ with symptoms typically emerging during early adulthood,^4^ while the lifetime prevalence for any psychotic disorder is around four percent.^5^^,^^6^ Despite therapeutic advances over the past half-century, psychotic disorders remain severely incapacitating and their care accounts for high direct and indirect costs. Prognoses are variable—although up to a third of those who experience a first episode of psychosis may recover,^7^ around a quarter go on to develop ‘treatment resistant’ symptoms with high levels of impairment and healthcare needs.^8^^,^^9^

‘Early Intervention for Psychosis’ (EIP) services were conceived to provide specialised intensive treatment and support for people in the early stages of a psychotic disorder. They have proven to be both clinically effective and cost-effective,^10^ with meta-analyses demonstrating superiority over ‘treatment as usual’ for a range of outcomes—psychotic symptom severity, treatment adherence and social functioning.^11^^,^^12^ As a result, EIP services are now considered the gold standard for treating early psychosis in the UK and internationally.^13^, ^14^, ^15^

However, important unanswered questions remain regarding processes of EIP care. Specifically, there is no consensus on which components of the interventions delivered by EIP services contribute to their observed benefits. EIP services generally provide pharmacotherapy as standard, but specific examples differ widely in the other components of care that they provide (including case management, psychotherapies, family interventions and social interventions).^16^, ^17^, ^18^, ^19^ There is conflicting evidence regarding the specific effects of some of these components, especially in the longer term.^20^, ^21^, ^22^

While standards exist to guide the implementation of EIP services,^23^ these have been based largely on expert opinion rather than comparisons of different models. As current guidelines recommend EIP services for those with early psychosis,^24^ it is important to identify which of the components they employ are most effective in achieving sustained or prolonged benefits to establish better-fit EIP service models and guide societal decisions about resource allocation and funding.

In this study, we conducted a comprehensive systematic review to identify studies comparing interventions for early psychosis. We performed a series of component network meta-analyses (cNMA) to determine which components of the interventions provided by EIP services are associated with sustained reduction of psychotic symptoms and improved acceptability.

## Methods

### Search strategy and selection criteria

We registered a protocol for our study in PROSPERO (CRD42017057420). We have reported the current manuscript according to the Preferred Reporting Items for Systematic Reviews and Meta-Analyses (PRISMA) statement^25^ and its extension for NMAs.^26^ The PRISMA-NMA checklist and a list of changes from the protocol are provided in the Appendix. We searched CENTRAL (published and unpublished), EMBASE, MEDLINE, CINAHL, PsycINFO and Web of Science from inception to February 2023 (see Appendix for full search strings). We inspected reference lists of published and unpublished trials, and conference proceedings for additional potentially eligible records. No language restrictions were applied.

We included all randomised controlled trials (RCT) comparing interventions for people aged ≥16 years old of any sex with ‘first episode psychosis’ or ‘early psychosis’ (defined as within five years of symptom onset at the time of the study baseline), against either a control comparison–such as ‘standard treatment’ or ‘treatment as usual’–or another eligible intervention. ‘Psychosis’ included any primary psychotic disorder, or affective disorder with psychotic symptoms according to standardised criteria such as the Diagnostic and Statistical Manual of Mental Disorders: DSM III-V, or the Internal Classification of Diseases: ICD 10–11. Interventions could be of any duration. We classified all interventions in terms of combinations of 5 pre-specified components of care—case management, pharmacotherapy, psychological interventions, family interventions and social interventions. Interventions were classified based on the descriptions provided by original authors, including information about their structure. Table 1 provides the definitions of these components of interest for active/control study arms as a-priori and independently defined by two senior researchers in EIP (BL and PF). We excluded quasi-randomised trials and trials comparing interventions that could not be distinguished as different components (e.g., comparison of two different pharmacological interventions).

**Table 1:** Components of EIP interventions and their definitions.

Component	Abbreviation	Definition
Pharmacotherapy	MED	Provision of any drug treatment intended to treat psychotic symptoms. As well as antipsychotics (including depot and oral formulations), this may also include other novel classes of medication under investigation such as stimulants.
Case management	CM	Any model of care involving provision of individualised treatment with a specific named ‘case manager’. The ‘case manager’ role is variably named but must act as a fixed point of contact for the individual receiving treatment during the course of their care. Example models of care include ‘care coordination’, ‘intensive case management’ and ‘assertive community treatment’.
Psychological intervention	PSY	Provision of any individual or group psychological treatment intended to treat, or ameliorate the consequences of, psychotic symptoms (excluding family therapy). Examples include cognitive-behavioural therapy for psychosis (CBTp), cognitive remediation, acceptance and commitment therapy or psychodynamic psychotherapy. Psychoeducation alone was not considered psychotherapy as this was felt to fall within the remit of case management.
Family intervention	FI	Provision of any intervention involving carers or family members of people with psychosis. Examples include family therapy and targeted carer support programmes.
Social intervention	SI	Provision of any intervention intended to address adverse social conditions resulting from psychotic symptoms (difficulties with education, employment, housing, finances etc). Examples include social skills or vocational training programmes, supported employment placements or optimisation of social welfare packages by a social worker.

Three researchers (RW, JA, AM) independently assessed the eligibility of retrieved records at title/abstract and full-text phases, and extracted data in triplicate. The same researchers classified all studies, treatment arms and their constituent components according to the definitions in Table 1, using information from published reports and by contacting original investigators for clarification where necessary. Inter-rater reliability was calculated for the judgement of which components were present in each trial arm using percentage agreement. Any disagreements were solved by consensus.

### Outcome measures

Our primary outcomes were (i) change in severity of positive and negative psychotic symptoms as measured on a validated scale at 3-month and 1-year follow-up (after the initiation of the intervention), and (ii) discontinuation from treatment due to any reason (which we note may have included recovery and is not necessarily indicative of a poor outcome). For the assessment of psychotic symptoms, we included any rating scale with established reliability and validity (see summary of included studies in Appendix for scales). Our secondary outcomes were (i) severity of depressive symptoms and (ii) social functioning assessed using a continuous validated rating scale at 1-year follow-up.

### Statistical analysis

For each outcome we initially performed standard pairwise meta-analyses using a random-effects model for direct comparisons of any pair of interventions occurring in two or more studies. Where studies used different rating scales to assess outcomes, data were pooled using standardized mean difference (SMD) scores (Hedges-adjusted *g* scores).^27^ Dropouts were compared using risk ratios (RRs). We examined the distribution of potential effect modifiers (year of publication, mean age, percentage of male participants and duration of the intervention) across comparisons by visually comparing box-plots to assess for violations of the transitivity assumption. This approach has been employed in network meta-analytical models to explore the distribution of effect modifiers across comparisons.^28^, ^29^, ^30^, ^31^

We then performed a random-effects NMA to synthesize the available evidence from the network. We produced league tables with summary comparative effect sizes (SMDs or RRs) for each pair of interventions, with an accompanying ‘intervention effectiveness hierarchy’ based on the cumulative P scores.^32^ We assessed network statistical heterogeneity by comparing heterogeneity variance parameters (τ2) from NMA models with their empirical distribution.^33^ We evaluated global inconsistency using the design-by-treatment test and local inconsistency using the back-calculation method (comparing direct and indirect estimates).^34^ We assessed the normality assumption in the context of SMD meta-analysis by calculating mean/SD ratios for continuous primary outcomes in each intervention arm for included studies.

Finally, we performed a random-effects component NMA in which the effects of composite interventions were expressed as the sum of the effects of their constituent components (additive assumption). Using this model, we estimated component-specific incremental SMDs (iSMDs) and risk ratios (iRRs) for continuous and binary outcomes, respectively. We produced league tables with summary comparative effect sizes (SMDs or RRs) for each pair of components. We conducted sensitivity analyses for the component network meta-analysis excluding trials with participants aged <18 and with interventions lasting >3 years in order to ensure that our findings were not overly influenced by this relatively small group. We conducted all the NMAs in a frequentist setting. Full details of statistical models and fitting procedures are available in the Appendix. All analyses were performed using R version 4.3.1 and the *meta* and *netmeta* packages.

### Risk of bias and certainty of evidence

Two researchers (RW and JA) independently assessed the risk of bias for each primary outcome and study using the Risk of Bias 2 tool.^35^ This assessment was conducted for two of our primary outcomes (negative symptoms at 3 months and 1 year follow-up). We selected these outcomes based on evidence that negative symptoms may be particularly important for predicting longer-term prognosis—multiple studies have highlighted these as the best symptomatic predictor of functioning in the FEP population, both cross-sectionally and longitudinally.^36^, ^37^, ^38^, ^39^ Any disagreement was resolved through discussion or through consultation with study supervisors (BL and AC).

Where at least 10 studies were available, we assessed the small study effects (including publication bias) by examining contour-enhanced funnel plots of pairwise meta-analyses between all arms vs. ‘pharmacotherapy + case management’ interventions (the most common ‘standard treatment’ intervention).

We assessed the certainty of evidence using the Confidence in Network Meta-Analysis (CINeMA) framework.^40^

### Role of the funding source

The funders had no role in study design, data collection, data analysis, data interpretation, or writing of the report.

## Results

**Fig. 1:**
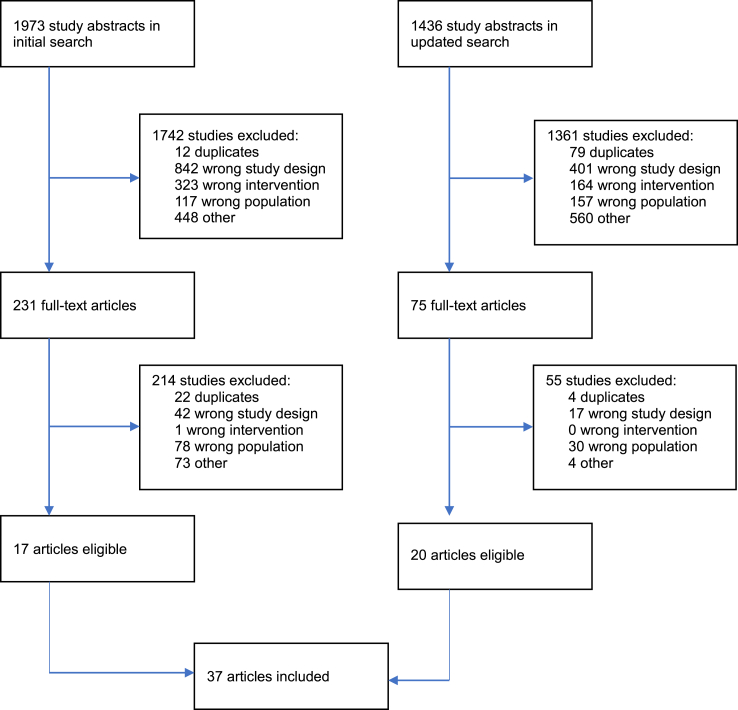
Prisma flow diagram.

In total, we retrieved 3409 references. After initial screening and full-text examination, 37 trials including a total of 4599 participants were included in our systematic review (Fig. 1). See Appendix for a summary of included studies and table of publications years/geographical distributions.

**Fig. 2:**
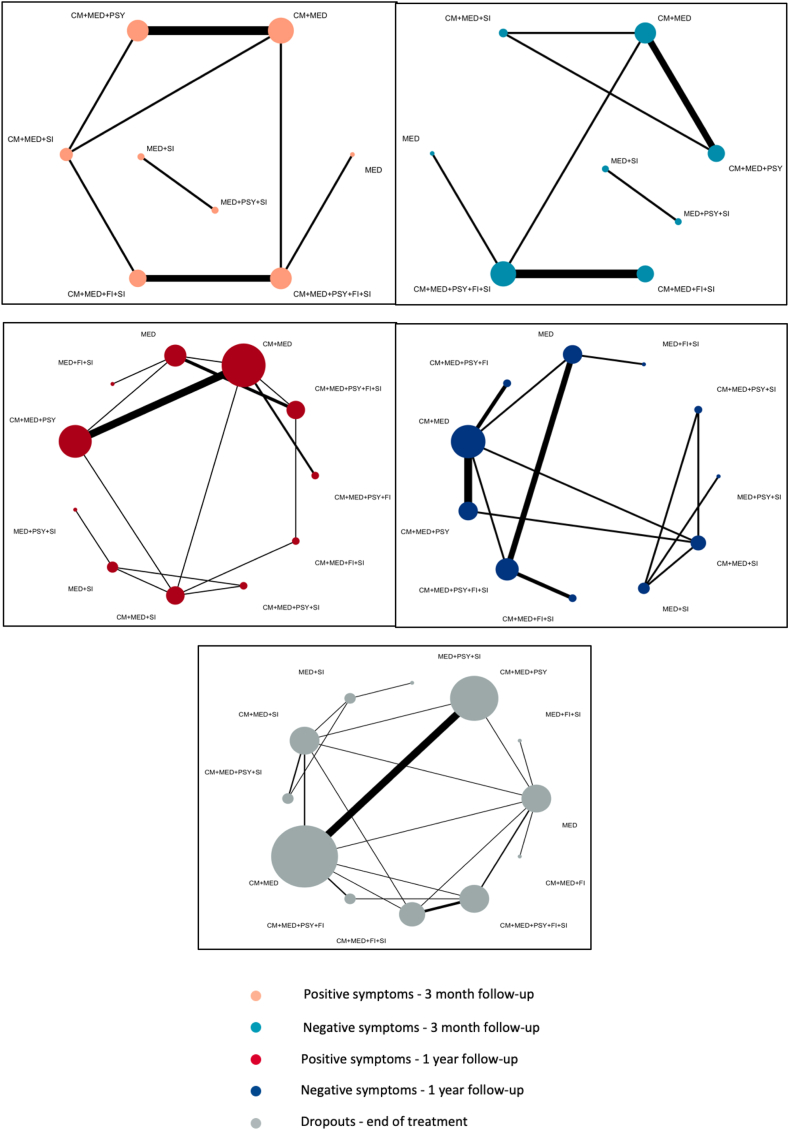
Network structure for primary outcomes. Network structure for the 5 primary outcomes examined in this article. Nodes represent combinations of components, and lines denote trials performing the corresponding comparison. The width of the lines is proportional to the number of trials comparing each pair of treatments. The size of the nodes is proportional to the number of randomised participants.

Included participants had a mean age of 25.8 years (SD 6.0). Sex was reported for 4496 participants, of which 2877 were male [64.0%]. Of the 37 trials, 34 compared two intervention arms, with the remaining studies comparing three arms. Across the identified interventions, the most prevalent component was ‘pharmacotherapy’ (included in 77 out of 77 arms, 100%) and the least was ‘family intervention’ (24 out of 77 arms, 31%). The network geometry for primary outcomes is shown in Fig. 2.

The interrater reliability of judgements for components was excellent, with an average percentage agreement of 92.1%. For the outcome ‘negative symptoms at 3 month follow-up’ risk of bias was rated as ‘low’ in 2 studies, ‘some concerns’ in 6, and ‘high’ in 1. For the outcome ‘negative symptoms at 1 year follow-up’ risk of bias was rated as ‘low’ in 4 studies, ‘some concerns’ in 7, and ‘high’ in 1 (see Appendix). Contour-enhanced funnel plots did not show any evidence of publication bias (see Appendix).

The distribution of potential effect modifiers across comparisons did not suggest violation of the transitivity assumption, although we acknowledge that the number of studies per comparison was small (see Appendix). However, as two comparisons involved interventions of particularly long duration (>3 years), we examined their impact on the overall analyses by excluding these in a sensitivity analysis (see Appendix). Examination of mean/SD ratios did not suggest violation of the normality assumption.

**Fig. 3:**
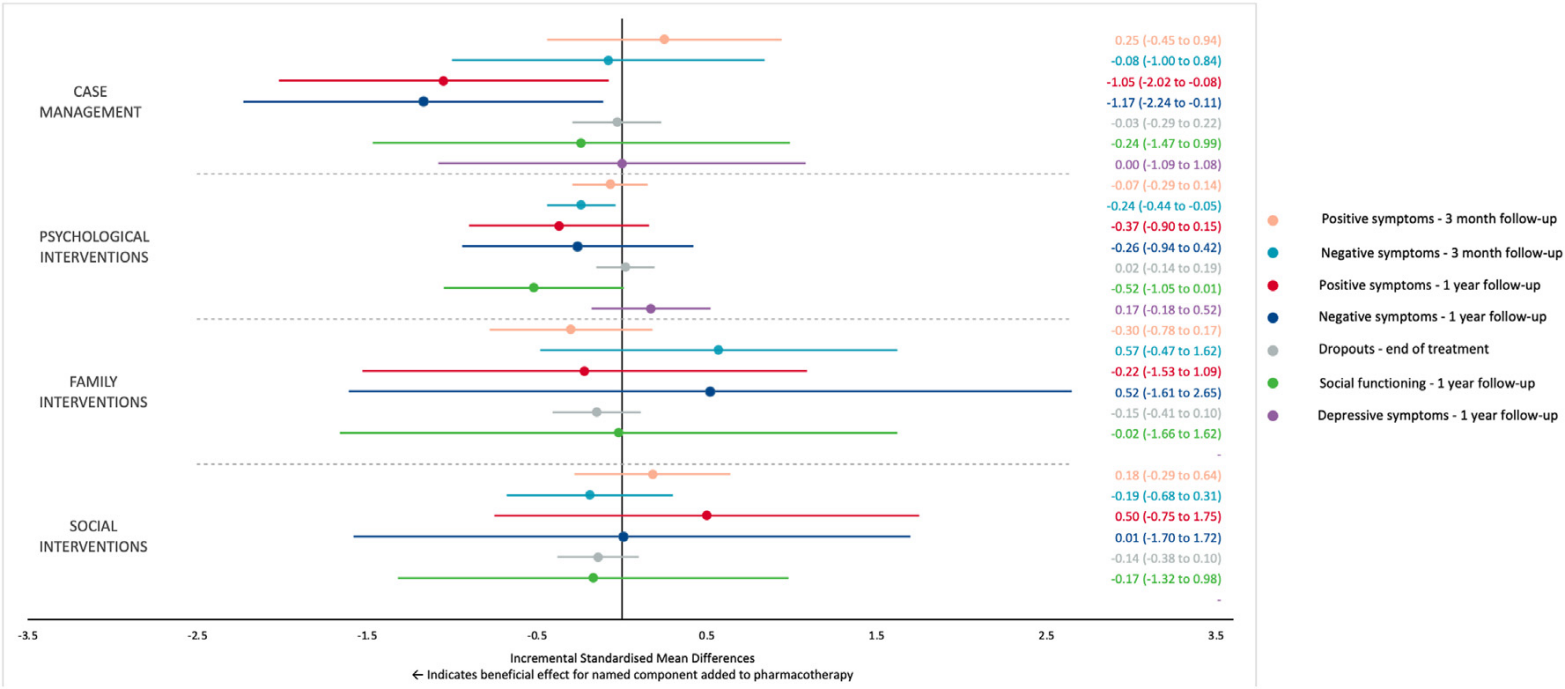
Forest plot of component network meta-analysis for primary and secondary outcomes. This plot shows the estimated component-specific incremental standardised mean differences of adding the row-defining component to an EIP package (including pharmacotherapy as standard), for each outcome. For dropouts, incremental risk ratios were converted to incremental standardised mean differences for plotting purposes.^41^ It was not possible to examine the effect of family interventions or social interventions for the outcome ‘depressive symptoms’ due to insufficient studies to isolate these components.

The number of studies and closed loops in the networks limited the assessment of consistency. Design-by-treatment tests for global inconsistency were statistically significant in the model for dropouts (Q = 18.59, 8 degrees of freedom, p = 0.02) and negative symptoms at 1-year follow-up (Q = 40.95, 3 degrees of freedom, p < 0.001). The back-calculation method did not identify any comparisons with evidence of local inconsistency for dropouts. Three out of five comparisons showed evidence of local inconsistency for negative symptoms at 1-year follow-up. Full results of the component network meta-analyses are available in the Appendix. Table 2 shows the comparative efficacy of any pair of specific components for all outcomes. Fig. 3 shows the incremental effect of adding a specific component to an EIP package across the considered outcomes.

**Table 2:** League table.

Comparative performance of single components. Negative standardised mean differences (lower triangle) or risk ratios lower than 1 (upper triangle) indicate that the row component is better than the column component.

As pharmacotherapy was present in all trial arms, it was not possible to differentiate the incremental effect of pharmacotherapy in the component analysis. Values for other components therefore represent the incremental standardised mean difference (beneficial or detrimental effect) of adding each component to an EIP package which includes pharmacotherapy as standard. There was suggestive evidence that the addition of psychological interventions was beneficial for reducing rates of negative psychotic symptoms at 3-month follow-up (iSMD, −0.24; 95% CI, −0.44 to −0.05, p = 0.014).

At 1-year follow-up, the addition of case management was beneficial for reducing rates of negative psychotic symptoms (iSMD, −1.17; 95% CI, −2.24 to −0.11, p = 0.030) and positive psychotic symptoms (iSMD, −1.05; 95% CI, −2.02 to −0.08, p = 0.033). No single component was associated with clinically important differences in the rates of dropouts by end of treatment.

In terms of our secondary outcomes, we found preliminary evidence that the addition of psychological interventions may vary from no clinically relevant effect to an important improvement of social functioning (iSMD, −0.52; 95% CI, −1.05 to 0.01, p = 0.052) one year after the treatment delivery. No other single component was associated with important differences in reducing depressive symptoms at 1-year follow-up (note that the component ‘pharmacotherapy’ in this study represents a grouping of all medications intended to treat psychotic symptoms—information on other medications, such as antidepressants, was not available—these may have been received by some participants in some studies, but it was not possible to control for this).

Our sensitivity analysis examining the effect of excluding trials with participants aged <18 and those with interventions lasting >3 years resulted in comparable findings (full results are available the Appendix).

The certainty of the evidence for all comparisons measured with CINeMA was rated as very low, due largely to the small number of studies resulting in high imprecision and heterogeneity parameters. Full information on CINeMA is described in the Appendix.

## Discussion

We conducted a comprehensive systematic review and several NMAs to reflect the breadth of currently available interventions for early psychosis. We also assessed the performance of specific components variably combined in these interventions based on 37 trials. This is the first meta-research study examining the comparative efficacy of components of EIP care. We used rigorous evidence synthesis methods to minimise the risk of selection bias and ensure a comprehensive representation of the available evidence. The quality of included studies was generally adequate, and our processes for characterising these studies achieved excellent interrater agreement.

The incremental benefits in sustained clinical improvements observed by adding specific components to pharmacotherapy were overall modest. This finding is expected, as antipsychotic medications have a robust evidence base for management of psychotic disorders^42^^,^^43^ and early psychosis specifically.^44^ Current guidelines^24^ acknowledge that stabilisation with pharmacotherapy is necessary particularly early in the clinical course prior to augmentation with psychosocial interventions, which have generally been thought to confer additional benefits in the medium to longer-term.^45^

The addition of psychological interventions did demonstrate advantages for negative symptoms at 3-month follow-up, with preliminary evidence for a potential effect on social functioning at 1-year follow-up. Effect sizes were small to moderate, in line with findings from previous studies (e.g., cognitive behavioural therapy for schizophrenia).^46^ No conclusions about the efficacy of any specific modality of psychological therapy can be drawn from these results. However, this profile of improvements has some clinical face validity in the context of psychological treatment frameworks for psychotic illness. For example, cognitive behavioural approaches for psychosis focus developing psychological ‘distance’ from psychotic experiences over time—a process which might be expected to yield early improvements in negative symptoms, with more gradual improvement in social functioning following. Although modest, these preliminary findings may provide explorative evidence that psychological interventions still have a role and should be made available as part of the regular package of EIP care.

The addition of case management also provided incremental benefits in reducing both positive and negative psychotic symptoms at 1-year follow-up. This corroborates findings from one of the flagship trials of an EIP model^47^ and a previous meta-analysis,^48^ both of which found that case management in an EIP setting was associated with greater reductions in psychotic symptoms than standard treatment (mainly based on pharmacotherapy).

This beneficial effect of case management for negative symptoms may be particularly important in light of the known limited effects of available pharmacological interventions,^49^ with current drugs yielding largely clinically insignificant results.^50^ The evidence of a persistent improvement after 1 year may indicate that the effects of case management take time to establish (previously suggested mechanisms include improved therapeutic alliance and engagement with continued treatment and monitoring).^51^ However, there is some suggestion that these benefits may also be resilient to extinction–a recent observational study which also reproduced specific improvements in negative symptoms with case management, did so over an even longer follow-up period.^52^

Family interventions were not associated with any clear benefits for any of our outcomes. This aligns (unsurprisingly) with the results of one of our included trials (the longest and largest study of a family intervention in an EIP setting to date),^53^ which found no differences from ‘standard treatment’ for primary or secondary outcomes. Similar results have previously been obtained from studies of family interventions for patients with established schizophrenia.^54^ However, it is worth reiterating that as this study estimates average effects, specific components such as family therapy may still have benefits at an individual level. The most recent consensus seems to be that family interventions may be beneficial predominantly for patients who reside in a particularly stressful family environment, or patients with a chronic illness who experience frequent relapses.^55^ Our results indicate that they may be better used targeted to these specific needs rather than necessarily being delivered as part of a standardised care package.

The inclusion of a social intervention component also failed to provide a sustained benefit 3 months and 1 year after the end of treatment. Social interventions have generally been considered a crucial factor in an effective EIP program, in the context of a supportive evidence base. Previous research has shown improvements in negative psychotic symptoms and social functioning specifically with social skills training compared to treatment as usual,^56^ and similarly positive findings exist for other social interventions such as vocational support.^57^ However, it should be noted that a more recent Cochrane review found no additional benefits from social skills training when compared to an active control ‘discussion group’ over several outcomes.^58^ It is possible that previous studies struggled to differentiate the effects of social interventions from those of case management, which may have been implemented simultaneously as part of a holistic intervention.

Guidelines for EIP service implementation currently include recommendations about the components of care that they provide.^59^ However, evidence for the comparative efficacy of components in this setting is lacking. Our results reinforce the importance of a comprehensive package of treatment components to optimise EIP outcomes, particularly in the longer term. Alongside pharmacotherapy, case management and psychological interventions may be particularly important components for reducing psychotic symptoms. Readers should note that our analyses are limited by several important factors outlined below, and that the evidence underlying these conclusions is still relatively imprecise.

The associations we have identified emphasise the need for further studies in this field. Unfortunately, large scale randomised controlled trials involving head-to-head comparisons of different EIP service models are likely to be impractical. However, realist evaluations of services would certainly be possible using observational methods. Such research could use existing variation in components of care between services to compare the differential efficacy in components, in order to extend and consolidate our estimates and ultimately optimise EIP service delivery. A consensus regarding a common essential assessment battery for EIP outcomes would enable future comparative and meta-research studies to be conducted more reliably. We would reiterate existing recommendations^60^ that this should include patient-reported outcome measures to enable the provision of care around patients’ specific experiences, preferences and needs.

Several limitations must be noted. The analyses were limited by the relatively small number of studies and sparsely connected networks. Many of our interpretations are therefore based on indirect rather than direct comparisons, and according to our CINeMA analysis the evidence for all comparisons was rated as very low certainty. The small number of studies also precluded assessment of publication bias or sources of heterogeneity for some outcomes. Several of the studies that we included also had relatively small sample sizes, which may lead to small-sample bias.

Our classification of components, while based on expert opinion, represents a simplification of a complex reality–there are certainly different ways for a specific component to be delivered (e.g., the component ‘psychological interventions’ includes a range of possible modalities of psychological therapy, the component ‘pharmacotherapy’ includes a range of possible medications). Therefore, our analyses can only provide preliminary evidence on the effects of including typical examples of components at the initiation of an EIP intervention. More studies would be required in order to appreciate the effects of specific sub-types of components (e.g., at the level of a specific medication, modality of psychological therapy or social intervention). We also had to exclude several studies that trialled interventions with components that were not classifiable into our pre-specified scheme—e.g., physical exercise programmes^61^^,^^62^ or technology-assisted symptom management.^63^ While it is currently unlikely that there are sufficient studies to conduct a meaningful analysis of these interventions, future meta-research studies may benefit by examining a broader range of components.

Durations of treatment in the 37 included studies varied, and it was also not possible to assess to what degree participants continued to be exposed to components of care during follow-up periods beyond the period of active ‘treatment’ as defined in each study. For example, after an EIP intervention involving case management, pharmacotherapy, family intervention and psychological intervention, some participants may have been referred on to community mental health teams where they would continue to receive case management (albeit at differing intensity) and pharmacotherapy throughout the follow-up period, and some not. This may have the result of underestimating the true effects of components that were relatively likely to be provided outside of the active study period (e.g., case management).

We only assessed the transitivity assumption for a small number of possible effect modifiers. While these did not demonstrate any clear evidence of intransitivity, there is always a possibility that our network may have been confounded by other unobserved imbalances across comparisons, such as illness severity or sociodemographic differences.

Our statistical models assume additivity of component effects, or the absence of interactions between components (i.e. that for any given component c, the relative effect of [c + X] vs. X is equivalent to any combination of components X [not including c]). While the additivity assumption is a simplification that cannot fully capture the complexity of multi-component care treatments, its experimental nature provides a preliminary insight on whether specific components may be, overall, beneficial or detrimental. Unfortunately we were unable to formally test the additivity assumption due to inadequate power, and acknowledge that findings from our model assume an additive interaction between components.

Our analysis only included studies from our search until February 2023. Prior to the publication of this manuscript, we conducted an updated search to January 2024. As this search identified only four further eligible studies,^64^, ^65^, ^66^, ^67^ which would represent an increase of only 10% in our included participants, and would not add any new combinations of components—we felt that their inclusion would be unlikely to result in material changes to our results or conclusions and did not justify repeating our analysis. Finally, although we conducted a methodologically rigorous literature search, we cannot exclude the possibility that we failed to identify relevant published or unpublished studies.

In conclusion, this cNMA of EIP services has identified potentially helpful components of care beyond pharmacotherapy alone, for improving symptoms and social functioning in the longer-term following treatment. While additional benefits appear to be modest, future EIP programmes aiming to optimise outcomes may prioritise implementing effective case management and psychological interventions alongside pharmacotherapy. Efforts should continue to develop a gold-standard EIP service framework for individuals with early psychosis.

## Contributors

BRL and AC formulated the presented research question and designed the study. BRL and PF specified the components for inclusion in the analysis. DM, AK, SP, CC and FDC performed the initial search, selected studies and extracted data. RW, JA and AM performed the updated search, selected the studies and extracted data. AM and SP verified the data. RW and EGO analysed the data. RW and EGO interpreted the results. RW wrote the first draft of the manuscript. All authors had access to all the data and provided critical input and revisions to the draft manuscripts and approved the final manuscript. BRL and AC had final responsibility for the decision to submit for publication.

## Data sharing statement

All authors had access to the full study dataset, which can be made available on request.

## Declaration of interests

EGO has received research and consultancy fees from Angelini Pharma, for work not related to this project. FDC is an employee of Boehringer Ingelheim International. AC has received research, educational and consultancy fees from INCiPiT (Italian Network for Paediatric Trials), CARIPLO Foundation, Lundbeck and Angelini Pharma, for work not related to this project. RW is supported by an NIHR Doctoral Fellowship (grant NIHR302320).
