# Supplementary material for: Comparing interventions for early psychosis: a systematic review and component network meta-analysis
Source: eClinicalMedicine. 2024 Mar 14;70:102537. doi: 10.1016/j.eclinm.2024.102537 (PMC10955207; doi:10.1016/j.eclinm.2024.102537)
Supplement: Appendix [file mmc1.docx]

# **COMPARING INTERVENTIONS FOR EARLY PSYCHOSIS: A SYSTEMATIC REVIEW AND COMPONENT NETWORK META-ANALYSIS**

Ryan Williams, Edoardo G Ostinelli, Joel Agorinya, Amedeo Minichino, Franco De Crescenzo, Daniel Maughan, Stephen Puntis, Charlotte Cliffe, Ayse Kurtulmus, Belinda Lennox, Andrea Cipriani

Supplementary Appendix

# INDEX

1. [FULL SEARCH STRATEGY](#_ONLINE_SEARCH_STRINGS)
2. [DESCRIPTION OF STATISTICAL MODELS](#_DESCRIPTION_OF_STATISTICAL)
3. [CHANGES FROM THE PUBLISHED PROTOCOL](#_CHANGES_FROM_THE)
4. [COMPONENTS OF EIP INTERVENTIONS AND DEFINITIONS](#_COMPONENTS_OF_EIP)
5. [STUDY CHARACTERISTICS](#_STUDY_CHARACTERISTICS)
6. [LOCATIONS / PUBLICATION YEARS OF INCLUDED TRIALS](#_LOCATIONS_AND_PUBLICATION)
7. [ASSESSMENT OF TRANSITIVITY](#_TRANSITIVITY)
8. [ASSESSMENT OF NORMALITY](#_ASSESSMENT_OF_NORMALITY)
9. PAIRWISE AND NETWORK META-ANALYSIS FOR EACH OUTCOME
   1. [SUMMARY](#_PAIRWISE_META-ANALYSES/_NMA_1)
   2. [POSITIVE PSYCHOTIC SYMPTOMS – 3 MONTHS](#_PRIMARY_OUTCOME_:)
      1. [Network league plot](#_PAIRWISE_AND_NETWORK)
      2. [Evaluation of inconsistency](#_INCONSISTENCY)
      3. [Ranking of intervention packages](#_RANKING_OF_TREATMENTS)
      4. [Publication bias/ small study effects](#_PUBLICATION_BIAS/_SMALL)
      5. [Network Plot](#_NETWORK_PLOT)
   3. [NEGATIVE PSYCHOTIC SYMPTOMS – 3 MONTHS](#_PRIMARY_OUTCOME_:_1)
      1. [Network league plot](#_PAIRWISE_AND_NETWORK)
      2. [Evaluation of inconsistency](#_INCONSISTENCY)
      3. [Ranking of intervention packages](#_RANKING_OF_TREATMENTS)
      4. [Publication bias/ small study effects](#_PUBLICATION_BIAS/_SMALL)
      5. [Network Plot](#_NETWORK_PLOT)
   4. [DROPOUTS – END OF TREATMENT](#_PRIMARY_OUTCOME_:_2)
      1. [Network league plot](#_PAIRWISE_AND_NETWORK)
      2. [Evaluation of inconsistency](#_ASSESSMENT_OF_INCONSISTENCY)
      3. [Ranking of intervention packages](#_RANKING_OF_TREATMENTS)
      4. [Publication bias/ small study effects](#_PUBLICATION_BIAS/_SMALL)
      5. [Network Plot](#_NETWORK_PLOT)
   5. [POSITIVE PSYCHOTIC SYMPTOMS – 1 YEAR](#_PRIMARY_OUTCOME_:_3)
      1. [Network league plot](#_PAIRWISE_AND_NETWORK)
      2. [Evaluation of inconsistency](#_EVALUATION_OF_INCONSISTENCY)
      3. [Ranking of intervention packages](#_RANKING_OF_TREATMENTS)
      4. [Publication bias/ small study effects](#_PUBLICATION_BIAS/_SMALL)
      5. [Network Plot](#_NETWORK_PLOT)
   6. [NEGATIVE PSYCHOTIC SYMPTOMS – 1 YEAR](#_PRIMARY_OUTCOME_:_4)
      1. [Network league plot](#_PAIRWISE_AND_NETWORK)
      2. [Evaluation of inconsistency](#_INCONSISTENCY)
      3. [Ranking of intervention packages](#_RANKING_OF_TREATMENTS)
      4. [Publication bias/ small study effects](#_PUBLICATION_BIAS/_SMALL)
      5. [Network Plot](#_NETWORK_PLOT)
   7. [DEPRESSIVE SYMPTOMS – 1 YEAR](#_SECONDARY_OUTCOME_:)
      1. [Network league plot](#_PAIRWISE_AND_NETWORK)
      2. [Evaluation of inconsistency](#_INCONSISTENCY)
      3. [Ranking of intervention packages](#_RANKING_OF_TREATMENTS)
      4. [Publication bias/ small study effects](#_PUBLICATION_BIAS/_SMALL)
      5. [Network Plot](#_NETWORK_PLOT)
   8. [SOCIAL FUNCTIONING – 1 YEAR](#_SECONDARY_OUTCOME_:_1)
      1. [Network league plot](#_PAIRWISE_AND_NETWORK)
      2. [Evaluation of inconsistency](#_INCONSISTENCY)
      3. [Ranking of intervention packages](#_RANKING_OF_TREATMENTS)
      4. [Publication bias/ small study effects](#_PUBLICATION_BIAS/_SMALL)
      5. [Network Plot](#_NETWORK_PLOT)
10. [COMPONENT NETWORK META-ANALYSIS](#_COMPONENT_NETWORK_META-ANALYSIS)
11. [SENSITIVITY ANALYSES](#_SENSITIVITY_ANALYSIS)
12. [CINeMA QUALITY ASSESSMENT OF COMPARISONS](#_CINEMA_:_EVALUATION_2)
13. [REFERENCES](#_REFERENCES)

# ONLINE SEARCH STRINGS

**Full search strategy**

**1. CENTRAL (via onlinelibrary.wiley.com)**

#1 psychoses

#2 psychosis:TI,AB,K

#3 (early psychosis):TI,AB,KY

#4 (first episode psychosis):TI,AB,KY

#5 (early intervention service):TI,AB,KY

#6 (early intervention services):TI,AB,KY

#7 (community care team ):TI,AB,KY

#8 (community care ):TI,AB,KY

#9 (counselling session ):TI,AB,KY

#10 counselling :TI,AB,KY

#11 (family therapy ):TI,AB,KY

#12 (individual placement support ):TI,AB,KY

#13 (individual placement ):TI,AB,KY

#14 (adherence coping ):TI,AB,KY

#15 (coping therapy):TI,AB,KY

#16 (home based care ):TI,AB,KY

#17 (home based ):TI,AB,KY

#18 (pharmacological treatment):TI,AB,KY

#19 #1 OR #2 OR #3 OR #4

#20 #5 OR #6 OR #7 OR #8 OR #9 OR #10 OR #11 OR #12 OR #13 OR #14 OR #15 OR #16 OR #17 OR #18

#21 #19 AND #20

**2. MEDLINE (via PubMed)**

1. Early Intervention[Mesh]

2. "Community Health Services"[Mesh]

3. "Community Mental Health Services"[Mesh]

4. "Counseling"[Mesh]

5. "Psychotherapy"[Mesh]

6. "Family Therapy"[Mesh]

7. "Home Care Services, Hospital-Based"[Mesh]

8. "Drug Therapy"[Mesh]

9. “early intervention service”[tiab]

10. “community care team”[tiab]

11. “individual placement support”[tiab]

12. “adherence therapy”[tiab]

13. #1 OR #2 OR #3 OR #4 OR #5 OR #6 OR #7 OR #8 OR #9 OR #10 OR #11 OR #12

14. "Psychotic Disorders"[Mesh]

15. Schizophrenia[Mesh]

16. psychosis[tiab]

17. “early psychosis”[tiab]

18. “first episode psychosis”[tiab]

19. “Randomized Controlled Trial"[Publication Type]

20. #14 OR #15 OR #16 OR #17 OR #18

21. #13 AND #19 AND #20

**3. CINAHL (via EBSCO HOST)**

1. (MM "Community Mental Health Services+")

2. (MM "Early Intervention+")

3. (MM "Counseling")

4. (MM "Psychotherapy+") OR (MH "Psychotherapy, Brief") OR (MH "Psychotherapy, Psychodynamic") OR (MH "Psychotherapy, Group") OR (MH "Cognitive Therapy") OR (MH "Validation Therapy")

5. (MH "Family Therapy") OR (MH "Family Therapy (Iowa NIC)")

6. (MM "Community Mental Health Services+")

7. (MM "Drug Therapy")

8. (MM "Support, Psychosocial")

9. (“early intervention” OR “community care team” OR “individual placement support” OR “adherence coping therapy”)

10. #1 OR #2 OR #3 OR #4 OR #5 OR #6 OR #7 OR #8 OR #9

11. MH "Clinical Trials+"

12. PT Clinical trial

13. TI clinic* N1 trial* or AB clinic* N1 trial*

14. TI ( singl* or doubl* or trebl* or tripl* ) and TI ( blind* or mask* )

15. AB ( singl* or doubl* or trebl* or tripl* ) and AB ( blind* or mask* )

16. TI randomi?ed control* trial* or AB randomi?ed control* trial*

17. MH "Random Assignment"

18. TI random* allocat* or AB random* allocat*

19. #11 OR #12 OR #13 OR #14 OR #15 OR #16 OR #17 OR #18

20. (MM "Psychotic Disorders+")

21. ("Schizophrenia" OR psychosis OR “early psychosis” OR “first episode psychosis”)

22. #20 OR #21

23. #10 AND #19 AND 22

**4. Web of Science (via THOMSON REUTERS)**

1. "Early Intervention" OR "Community Health Services" OR "Community Mental Health Services" OR "Counseling" OR "Psychotherapy" OR "Family Therapy" OR "Home Care Services, Hospital-Based" OR "Drug Therapy" OR “early intervention service” OR “community care team” OR “individual placement support” OR “adherence coping therapy”)

2. ("Psychotic Disorders" OR "Schizophrenia" OR psychosis OR “early psychosis” OR “first episode psychosis”)

3. TS= clinical trial* OR TS=research design OR TS=comparative stud* OR TS=evaluation stud* OR TS=controlled trial* OR TS=prospective stud* OR TS=random* OR TS=placebo* OR TS=(single blind*) OR TS=(double blind*)

4. #1 AND #2 AND #3

# DESCRIPTION OF STATISTICAL MODELS

Treatments in network meta-analysis (NMA) can be complex interventions. Some treatments may be combinations of others or have common components. The standard analysis provided by netmeta package in R is a NMA where all existing (single or combined) treatments are considered as different nodes in the network. Assume a two-arm study 𝑖 that compares intervention X (comprising, for example, components 𝑐1 and 𝑐2) versus intervention Y (comprising, for example, components 𝑐3 and 𝑐4) on a dichotomous outcome (e.g., recurrence). Then, using the log-odds ratios as an effect measure, the usual, random effects network meta-analysis (NMA) model can be written as follows:


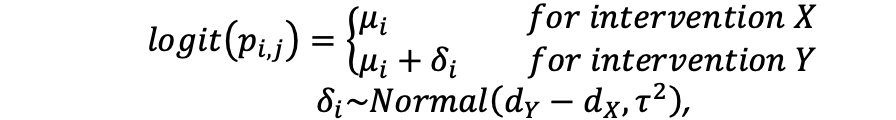


Here 𝑝𝑖,𝑗 denotes the probability of having an event in treatment arm 𝑗 of study 𝑖. 𝜏 denotes the heterogeneity standard deviation, assumed to be common for all log-odds ratios in the network. 𝜇𝑖 is a study-specific parameter, assumed independent across studies.

Exploiting the fact that some treatments are combinations of common components, an additive component network meta-analysis (CNMA) model can be used to separate the influence of individual components.

According to the cNMA model, adding a component 𝑐 to a composite intervention 𝑋 (which includes components other than 𝑐) will lead to an increase (or decrease) of the effectiveness of the X intervention that will only depend on 𝑐, but not on the other components included in 𝑋. For the case of a binary outcome (e.g., recurrence), this model estimates component-specific incremental risk ratios ( 𝑖*RR*𝑐), defined as the risk ratio between interventions (𝑋 + 𝑐) and 𝑋. Combining the various estimates of the *𝑖RR* of different components allows the estimation of RRs between any two composite interventions. Likewise, for continuous outcomes (e.g., depression endpoints), the model estimates component-specific incremental SMDs (𝑖𝑆𝑀𝐷), with larger values suggesting a larger impact on outcome.

This model assumes that the effect of a treatment combination is the sum of the effects of its components which implies that common components cancel out in comparisons. This model assumes no interaction between the components.

We fit the model using the netcomb command in the netmeta package in R. This function implements the additive model in a frequentist way.^1^

# CHANGES FROM THE PROTOCOL

Changes and clarifications to the published protocol:

1.

In the published protocol of this study, the original plan was to conduct a network meta-analysis to explore the comparative efficacy of interventions for early psychosis. However, during the course of the study, a novel technique known as component network meta-analysis emerged, with the capability of examining the constituent components of complex interventions. In light of this innovative methodology and its potential to provide deeper insights into the comparative effectiveness of components of care for early psychosis, a decision was made to adapt the study design from a standard network meta-analysis to a component network meta-analysis.

2.

In addition to our listed primary and secondary outcomes, we had planned to include reductions in psychotic symptoms at 6 weeks; adverse events such as weight gain, metabolic features, QT prolongation and extrapyramidal symptoms; and dropouts specifically due to adverse events. However, these outcomes were rarely reported and there were not enough available data for network analysis after data extraction.

3.

We had planned to conduct sensitivity analyses examining the impact of

(i) excluding studies where imputation of standard deviations was necessary; and

(ii) excluding studies focusing on patients with early psychosis and other comorbid medical or psychiatric conditions

However, this was not necessary due to none of our included studies meeting these criteria.

4.

In the published protocol of this study, we had intended to exclude all studies including participants aged <18. However, in the course of the literature search it became clear that a large proportion of studies of EIP services included some adolescents due to the real-world clinical remit of these services (i.e. accepting referrals for patients aged 16-35). We therefore decided to include studies with participants aged ≥16. However, we still excluded studies which only involved children (ie studies must also have included participants aged >18). We examined the impact of including these additional studies on the conclusions from the component network meta-analysis in a sensitivity analysis – see section below.

5.

In the published protocol of this study, we intended to include all studies regardless of the duration of the intervention under investigation. However, in the course of the literature search it became apparent that the duration of eligible interventions varied widely, with possible implications for transitivity. Currently, a maximum duration for EIP intervention of 3 years is a widely used standard in the United Kingdom^2^ and elsewhere.^3^ We made the decision to conduct an additional sensitivity analysis examining the effect of including studies which exceeded this duration – see section below.

# COMPONENTS OF EIP INTERVENTIONS AND DEFINITIONS

| **Component** | **Abbreviation** | **Definition** |
| --- | --- | --- |
| Pharmacotherapy | MED | Provision of any drug treatment intended to treat psychotic symptoms. As well as antipsychotics (including depot and oral formulations), this may also include other novel classes of medication under investigation such as stimulants. |
| Case Management | CM | Any model of care involving provision of individualised treatment with a specific named ‘case manager’. The ‘case manager’ role is variably named but must act as a fixed point of contact for the individual receiving treatment during the course of their care. Example models of care include ‘care coordination’, ‘intensive case management’ and ‘assertive community treatment’. |
| Psychological  Intervention | PSY | Provision of any individual or group psychological treatment intended to treat, or ameliorate the consequences of, psychotic symptoms (excluding family therapy). Examples include cognitive-behavioural therapy for psychosis (CBTp), cognitive remediation, acceptance and commitment therapy or psychodynamic psychotherapy. Psychoeducation alone was not considered psychotherapy as this was felt to fall within the remit of case management. |
| Family  Intervention | FI | Provision of any intervention involving carers or family members of people with psychosis. Examples include family therapy and targeted carer support programmes. |
| Social  Intervention | SI | Provision of any intervention intended to address adverse social conditions resulting from psychotic symptoms (difficulties with education, employment, housing, finances etc). Examples include social skills or vocational training programmes, supported employment placements or optimisation of social welfare packages by a social worker. |

# STUDY CHARACTERISTICS

| **Study** |  |  | **Population** | | | **Intervention** | | **Outcome Measures** | | |
| --- | --- | --- | --- | --- | --- | --- | --- | --- | --- | --- |
| **Study** | **Country** | **Diagnostic Criteria** | **N Randomised** | **Age (years)**  **Mean (SD)** | **Men %** | **Components** | **Duration (weeks)** | **Positive / negative symptoms** | **Depressive symptoms** | **Social functioning** |
| Alvarez-Jiminez 2021^4^ | Australia | DSM-IV | 86 | 21.0 (2.9) | 52.3 | CM, MED, SI | 78 | Positive and Negative Syndrome Scale for Schizophrenia | Calgary Depression Scale for Schizophrenia | Personal and Social Performance Scale |
|  | Australia | DSM-IV | 84 | 20.8 (2.8) | 53.6 | CM, MED | 78 | Positive and Negative Syndrome Scale for Schizophrenia | Calgary Depression Scale for Schizophrenia | Personal and Social Performance Scale |
| Bertelsen 2008^5^ | Denmark | ICD-10 | 275 | 26.6 (6.4) | 58 | CM, MED, FI, SI | 104 | Scale for the Assessment of Positive Symptoms | Not assessed | Not assessed |
|  | Denmark | ICD-10 | 272 | 26.6 (6.3) | 60 | CM, MED | 104 | Scale for the Assessment of Positive Symptoms | Not assessed | Not assessed |
| Cechnicki 2017^6^ | Poland | DSM-IV | 40 | 27.4 (5.6) | 42 | CM, MED, PSY, FI | 156 | Brief Psychiatric Rating Scale | Not assessed | Not assessed |
|  | Poland | DSM-IV | 40 | 25.9 (6.1) | 44 | CM, MED | 156 | Brief Psychiatric Rating Scale | Not assessed | Not assessed |
| Chan 2009^7^ | China | DSM-IV | 36 | 34.2 (10.1) | 72.2 | CM, MED, FI, SI | 13 | Brief Psychiatric Rating Scale | Not assessed | Not assessed |
|  | China | DSM-IV | 37 | 36.3 (13.0) | 59.5 | CM, MED, SI | 13 | Brief Psychiatric Rating Scale | Not assessed | Not assessed |
| Chien 2019^8^ | Hong Kong | DSM-IV | 60 | 24.2 (7.2) | 56.7 | CM, MED, PSY, SI | 26 | Positive and Negative Syndrome Scale for Schizophrenia | Not assessed | Specific Level of Functioning Scale |
|  | Hong Kong | DSM-IV | 60 | 25.8 (6.7) | 53.3 | CM, MED, SI | 26 | Positive and Negative Syndrome Scale for Schizophrenia | Not assessed | Specific Level of Functioning Scale |
|  | Hong Kong | DSM-IV | 60 | 25.4 (6.8) | 56.7 | MED, SI | 26 | Positive and Negative Syndrome Scale for Schizophrenia | Not assessed | Specific Level of Functioning Scale |
| Chien 2019^9^ | Hong Kong | not specified | 67 | 28.9 (9.5) | 55.22 | MED, PSY, SI | 12 | Positive and Negative Syndrome Scale for Schizophrenia | Not assessed | Specific Level of Functioning Scale |
|  | Hong Kong | not specified | 67 | 29.5 (10.0) | 53.73 | MED, SI | 12 | Positive and Negative Syndrome Scale for Schizophrenia | Not assessed | Specific Level of Functioning Scale |
| Gafoor 2010^10^ | UK | ICD-10 | 71 | 26.0 (6.0) | 55 | CM, MED, PSY, FI, SI | 78 | Positive and Negative Syndrome Scale for Schizophrenia | Not assessed | Not assessed |
|  | UK | ICD-10 | 73 | 26.6 (6.4) | 74 | CM, MED | 78 | Positive and Negative Syndrome Scale for Schizophrenia | Not assessed | Not assessed |
| De Haan 2022^11^ | Netherlands | DSM-IV | 68 | 21.2 (2.6) | 84 | CM, MED, FI, SI | 208 | Positive and Negative Syndrome Scale for Schizophrenia | Not assessed | Not assessed |
|  | Netherlands | DSM-IV | 65 | 21.7 (2.9) | 83 | CM, MED, SI | 208 | Positive and Negative Syndrome Scale for Schizophrenia | Not assessed | Not assessed |
|  | Netherlands | DSM-IV | 65 | 21.3 (3.3) | 77 | MED | 208 | Positive and Negative Syndrome Scale for Schizophrenia | Not assessed | Not assessed |
| Drake 2014^12^ | UK | DSM-IV | 31 | 24.7 (5.2) | 68 | CM, MED, PSY | 42 | Positive and Negative Syndrome Scale for Schizophrenia | Not assessed | Not assessed |
|  | UK | DSM-IV | 31 | 23.4 (4.4) | 53 | CM, MED | 42 | Positive and Negative Syndrome Scale for Schizophrenia | Not assessed | Not assessed |
| Erickson 2020^13^ | Canada | not specified | 56 | 23.4 (3.5) | 80 | CM, MED, PSY, FI, SI | 52 | Brief Psychiatric Rating Scale | Not assessed | Global Assessment of Functioning |
|  | Canada | not specified | 53 | 22.7 (3.3) | 85 | CM, MED, PSY, FI | 52 | Brief Psychiatric Rating Scale | Not assessed | Global Assessment of Functioning |
| Fan 2005^14^ | China | CCMD-III | 53 | no data | not reported | CM, MED | 52 | Brief Psychiatric Rating Scale | Not assessed | Not assessed |
|  | China | CCMD-III | 53 | no data | not reported | MED | 52 | Brief Psychiatric Rating Scale | Not assessed | Not assessed |
| Gonzalez-Ortega 2021^15^ | Spain | DSM-IV | 92 | 27.7 (7.7) | 54.7 | CM, MED, PSY | 26 | Positive and Negative Syndrome Scale for Schizophrenia | Hamilton Depression Rating Scale | Global Assessment of Functioning |
|  | Spain | DSM-IV | 92 | 29.3 (10.0) | 63.7 | CM, MED | 26 | Positive and Negative Syndrome Scale for Schizophrenia | Hamilton Depression Rating Scale | Global Assessment of Functioning |
| Grawe 2006^16^ | Norway | DSM-IV | 30 | 25.0 (4.0) | 63 | CM, MED, PSY, FI | 104 | Positive and Negative Syndrome Scale for Schizophrenia | Not assessed | Not assessed |
|  | Norway | DSM-IV | 20 | 25.0 (4.3) | 60 | CM, MED | 104 | Positive and Negative Syndrome Scale for Schizophrenia | Not assessed | Not assessed |
| Haddock 1999^17^ | UK | DSM-IV | 10 | 28.1 (7.2) | 100 | CM, MED, PSY | 21 | Brief Psychiatric Rating Scale | Not assessed | Not assessed |
|  | UK | DSM-IV | 11 | 30.0 (7.9) | 81.8 | CM, MED | 21 | Brief Psychiatric Rating Scale | Not assessed | Not assessed |
| Hui 2022^18^ | Hong Kong | DSM-IV | 120 | 37.8 (8.2) | 45 | CM, MED, PSY, FI, SI | 104 | Positive and Negative Syndrome Scale for Schizophrenia | Calgary Depression Scale for Schizophrenia | Social and Occupational Functioning Assessment Scale |
|  | Hong Kong | DSM-IV | 120 | 39.1 (8.9) | 40.8 | MED | 104 | Positive and Negative Syndrome Scale for Schizophrenia | Calgary Depression Scale for Schizophrenia | Social and Occupational Functioning Assessment Scale |
| Jackson 2008^19^ | Australia | DSM-IV | 31 | 22.1 (3.3) | 61.2 | CM, MED, PSY | 14 | Brief Psychiatric Rating Scale | Not assessed | Not assessed |
|  | Australia | DSM-IV | 31 | 22.5 (3.8) | 83.9 | CM, MED | 14 | Brief Psychiatric Rating Scale | Not assessed | Not assessed |
| Jackson 2009^20^ | UK | ICD-10 | 36 | 24.1 (4.7) | 86.1 | CM, MED, PSY | 26 | Positive and Negative Syndrome Scale for Schizophrenia | Not assessed | Not assessed |
|  | UK | ICD-10 | 30 | 22.3 (4.4) | 60 | CM, MED | 26 | Positive and Negative Syndrome Scale for Schizophrenia | Not assessed | Not assessed |
| Kuipers 2004^21^ | UK | OPCRIT | 32 | 27.7 (8.9) | 71.9 | CM, MED, PSY, FI, SI | 39 | Positive and Negative Syndrome Scale for Schizophrenia | Not assessed | Not assessed |
|  | UK | OPCRIT | 27 | 27.9 (6.3) | 81.5 | CM, MED | 39 | Positive and Negative Syndrome Scale for Schizophrenia | Not assessed | Not assessed |
| Lecomte 2008^22^ | Canada | DSM-IV | 48 | 24.9 (5.2) | 65 | CM, MED, PSY | 13 | Brief Psychiatric Rating Scale | Not assessed | Not assessed |
|  | Canada | DSM-IV | 27 | 23.1 (5.2) | 83 | CM, MED, SI | 13 | Brief Psychiatric Rating Scale | Not assessed | Not assessed |
|  | Canada | DSM-IV | 54 | 25.4 (5.2) | 74 | CM, MED | 13 | Brief Psychiatric Rating Scale | Not assessed | Not assessed |
| Linke 2019^23^ | Poland | ICD-10 | 44 | 28.8 (5.2) | 60.6 | CM, MED, PSY, SI | 10 | Positive and Negative Syndrome Scale for Schizophrenia | Not assessed | Global Assessment of Functioning |
|  | Poland | ICD-10 | 44 | 28.9 (5.8) | 66.7 | CM, MED, SI | 10 | Positive and Negative Syndrome Scale for Schizophrenia | Not assessed | Global Assessment of Functioning |
| Liu 2019^24^ | China | DSM-IV | 40 | 26.4 (7.4) | 65 | CM, MED PSY | 10 | Positive and Negative Syndrome Scale for Schizophrenia | Not assessed | Personal and Social Performance Scale |
|  | China | DSM-IV | 40 | 28.6 (5.7) | 50 | CM, MED | 10 | Positive and Negative Syndrome Scale for Schizophrenia | Not assessed | Personal and Social Performance Scale |
| MacDougall 2019^25^ | Canada | DSM-IV | 11 | 23.7 (6.6) | 76.5 | CM, MED, PSY, FI, SI | 12 | Scale for the Assessment of Positive Symptoms | Not assessed | Not assessed |
|  | Canada | DSM-IV | 10 | 23.7 (6.6 | 76.5 | CM, MED, FI, SI | 12 | Scale for the Assessment of Positive Symptoms | Not assessed | Not assessed |
| Marchira 2019^26^ | Indonesia | ICD-10 | 50 | 21.9 (4.7) | 64 | MED, FI | 8 | Brief Psychiatric Rating Scale | Not assessed | Not assessed |
|  | Indonesia | ICD-10 | 50 | 22.9 (4.4) | 58 | MED | 8 | Brief Psychiatric Rating Scale | Not assessed | Not assessed |
| Myin-Germeys 2022^27^ | Netherlands | ICD-10 | 71 | 26.0 (6.0) | 41 | CM, MED PSY | 8 | Brief Psychiatric Rating Scale | Not assessed | Social and Occupational Functioning Assessment Scale |
|  | Netherlands | ICD-10 | 77 | 24.0 (6.0) | 61 | CM, MED | 8 | Brief Psychiatric Rating Scale | Not assessed | Social and Occupational Functioning Assessment Scale |
| Nuechterlein 2022^28^ | USA | DSM-IV | 29 | 21.6 (3.2) | 77 | CM, MED, PSY, SI | 52 | Not assessed | Not assessed | Global Functioning Scale |
|  | USA | DSM-IV | 31 | 21.9 (4.4) | 77 | CM, MED, SI | 52 | Not assessed | Not assessed | Global Functioning Scale |
| Palma 2019^29^ | Spain | DSM-IV | 35 | 25.5 (4.8) | 68.4 | CM, MED, PSY, FI | 52 | Positive and Negative Syndrome Scale for Schizophrenia | Not assessed | Global Assessment of Functioning |
|  | Spain | DSM-IV | 27 | 25.6 (4.9) | 81.5 | CM, MED | 52 | Positive and Negative Syndrome Scale for Schizophrenia | Not assessed | Global Assessment of Functioning |
| Penn 2011^30^ | USA | DSM-IV | 23 | 23.5 (3.9) | 60.9 | CM, MED, PSY, FI, SI | 36 | Positive and Negative Syndrome Scale for Schizophrenia | Not assessed | Not assessed |
|  | USA | DSM-IV | 23 | 21.0 (2.1) | 60.9 | CM, MED, FI, SI | 36 | Positive and Negative Syndrome Scale for Schizophrenia | Not assessed | Not assessed |
| Pos 2019^31^ | Netherlands | DSM-IV | 49 | 25.1 (4.5) | 75.5 | CM, MED, PSY, FI, SI | 12 | Brief Negative Symptom Scale | Not assessed | Global Assessment of Functioning |
|  | Netherlands | DSM-IV | 50 | 25.7 (4.4) | 86 | CM, MED, FI, SI | 12 | Brief Negative Symptom Scale | Not assessed | Global Assessment of Functioning |
| Robinson 2022^32^ | USA | DSM-IV | 223 | 23.2 (5.2) | 78 | CM, MED, PSY, FI, SI | 104 | Positive and Negative Syndrome Scale for Schizophrenia | Hamilton Depression Rating Scale | Heinrichs-Carpenter Quality of Life Scale |
|  | USA | DSM-IV | 181 | 23.1 (4.9) | 66 | MED | 104 | Positive and Negative Syndrome Scale for Schizophrenia | Hamilton Depression Rating Scale | Heinrichs-Carpenter Quality of Life Scale |
| Rocha 2020^33^ | Portugal | DSM-IV | 9 | 29.5 (13.4) | 83.3 | CM, MED, PSY | 20 | Positive and Negative Syndrome Scale for Schizophrenia | Not assessed | Personal and Social Performance Scale |
|  | Portugal | DSM-IV | 7 | 27.0 (6.1) | 100 | CM, MED | 20 | Positive and Negative Syndrome Scale for Schizophrenia | Not assessed | Personal and Social Performance Scale |
| Ruggeri 2015^34^ | Italy | ICD-10 | 272 | 29.3 (9.8) | 61 | CM, MED, PSY, FI | 39 | Positive and Negative Syndrome Scale for Schizophrenia | Not assessed | Not assessed |
|  | Italy | ICD-10 | 172 | 31.5 (9.2) | 54.7 | CM, MED | 39 | Positive and Negative Syndrome Scale for Schizophrenia | Not assessed | Not assessed |
| Sonmez 2020^35^ | Norway | DSM-IV | 32 | 28.6 (7.4) | 53.1 | CM, MED, PSY | 26 | Positive and Negative Syndrome Scale for Schizophrenia | Calgary Depression Scale for Schizophrenia | Global Assessment of Functioning |
|  | Norway | DSM-IV | 31 | 27.1 (6.3) | 64.5 | CM, MED | 26 | Positive and Negative Syndrome Scale for Schizophrenia | Calgary Depression Scale for Schizophrenia | Global Assessment of Functioning |
| Srihari 2015^36^ | USA | DSM-IV | 61 | 22.4 (4.5) | 82 | CM, MED, PSY, FI, SI | 52 | Positive and Negative Syndrome Scale for Schizophrenia | Not assessed | Not assessed |
|  | USA | DSM-IV | 59 | 22.6 (5.3) | 81 | MED | 52 | Positive and Negative Syndrome Scale for Schizophrenia | Not assessed | Not assessed |
| Uzenoff 2008^37^ | USA | DSM-IV | 13 | 25.3 (6.0) | 60 | CM, MED, PSY | 26 | Positive and Negative Syndrome Scale for Schizophrenia | Not assessed | Not assessed |
|  | USA | DSM-IV | 11 | 19.8 (3.5) | 40 | CM, MED | 26 | Positive and Negative Syndrome Scale for Schizophrenia | Not assessed | Not assessed |
| Valencia 2012^38^ | Mexico | DSM-IV | 44 | 24.5 (3.0) | 76.9 | MED, FI, SI | 52 | Positive and Negative Syndrome Scale for Schizophrenia | Not assessed | Not assessed |
|  | Mexico | DSM-IV | 44 | 24.1 (3.2) | 73.5 | MED | 52 | Positive and Negative Syndrome Scale for Schizophrenia | Not assessed | Not assessed |
| Van-Duin 2021^39^ | Netherlands | not specified | 34 | 28.1 (6.5) | 73.5 | CM, MED, PSY, SI | 26 | Positive and Negative Syndrome Scale for Schizophrenia | Not assessed | Global Assessment of Functioning |
|  | Netherlands | not specified | 39 | 26.0 (4.9) | 69.2 | CM, MED, SI | 26 | Positive and Negative Syndrome Scale for Schizophrenia | Not assessed | Global Assessment of Functioning |
| Vidarsdottir 2019^40^ | Iceland | not specified | 25 | 23.6 (3.4) | 92 | CM, MED, PSY, FI, SI | 12 | Positive and Negative Syndrome Scale for Schizophrenia | Depression, Anxiety and Stress Scale | Life Skills Profile Scale |
|  | Iceland | not specified | 24 | 24.8 (2.9) | 83.3 | CM, MED, FI, SI | 12 | Positive and Negative Syndrome Scale for Schizophrenia | Depression, Anxiety and Stress Scale | Life Skills Profile Scale |

# LOCATIONS AND PUBLICATION YEARS OF INCLUDED TRIALS


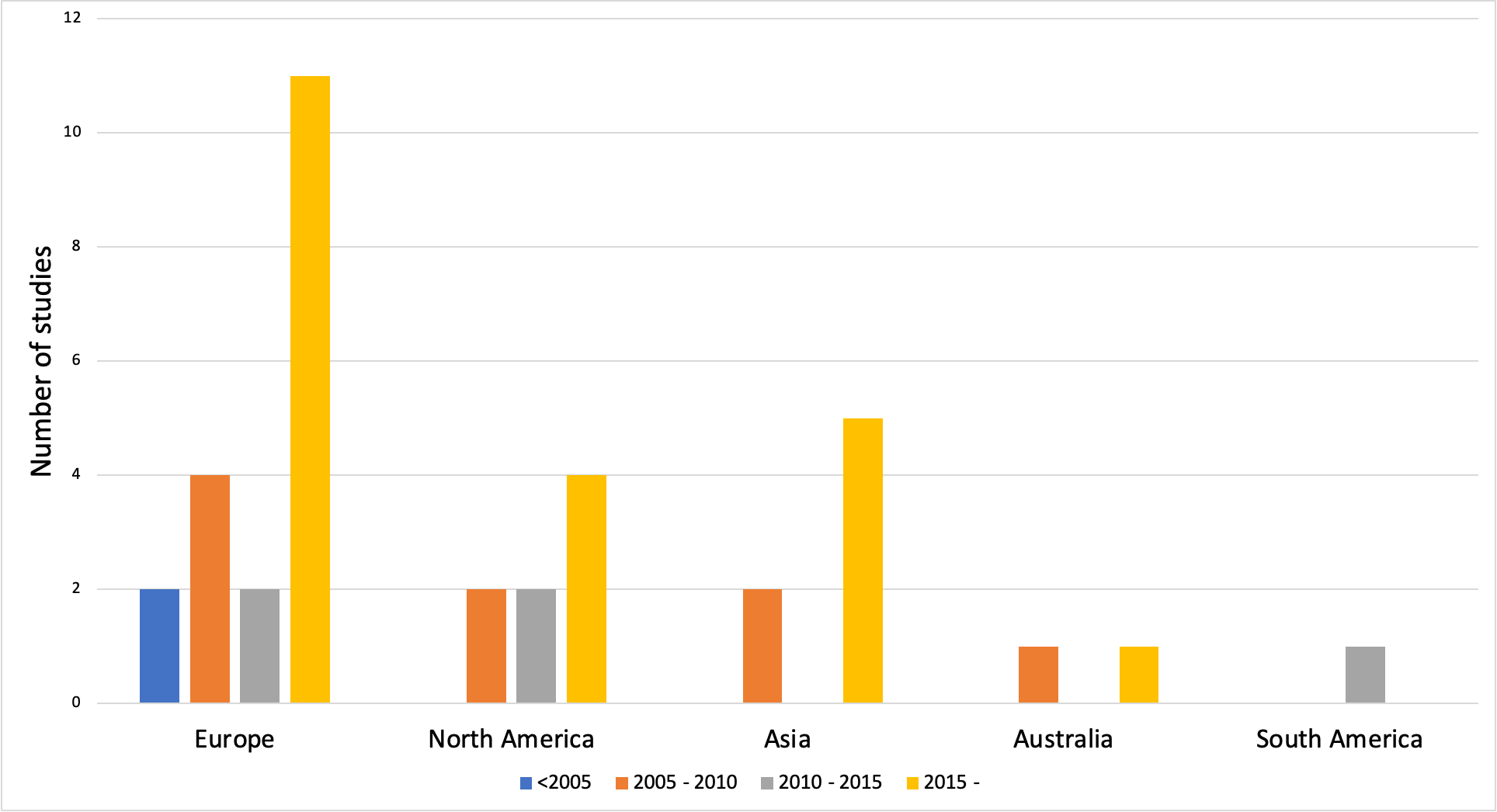


# ASSESSMENT OF TRANSITIVITY

We assessed the transitivity assumption by investigating the distribution of clinical and methodological effect modifiers across treatment comparisons for primary outcomes with boxplots. Values above Q3 + (1.5 x IQR) or below Q1 – (1.5 x IQR) are considered to be outliers. We considered the following effect modifiers: mean age of participants, the percentage of male participants, publication year and duration of intervention. The distribution of potential effect modifiers across comparisons did not suggest violation of the transitivity assumption, although we acknowledge that the number of studies per comparison was small. However, two comparisons did involve interventions of particularly long duration (>3 years) - we examined their impact on the overall analyses by excluding these in a sensitivity analysis, and none of the conclusions of our study were altered by this analysis (see section below ‘Sensitivity Analysis’).

1. Age - Mean


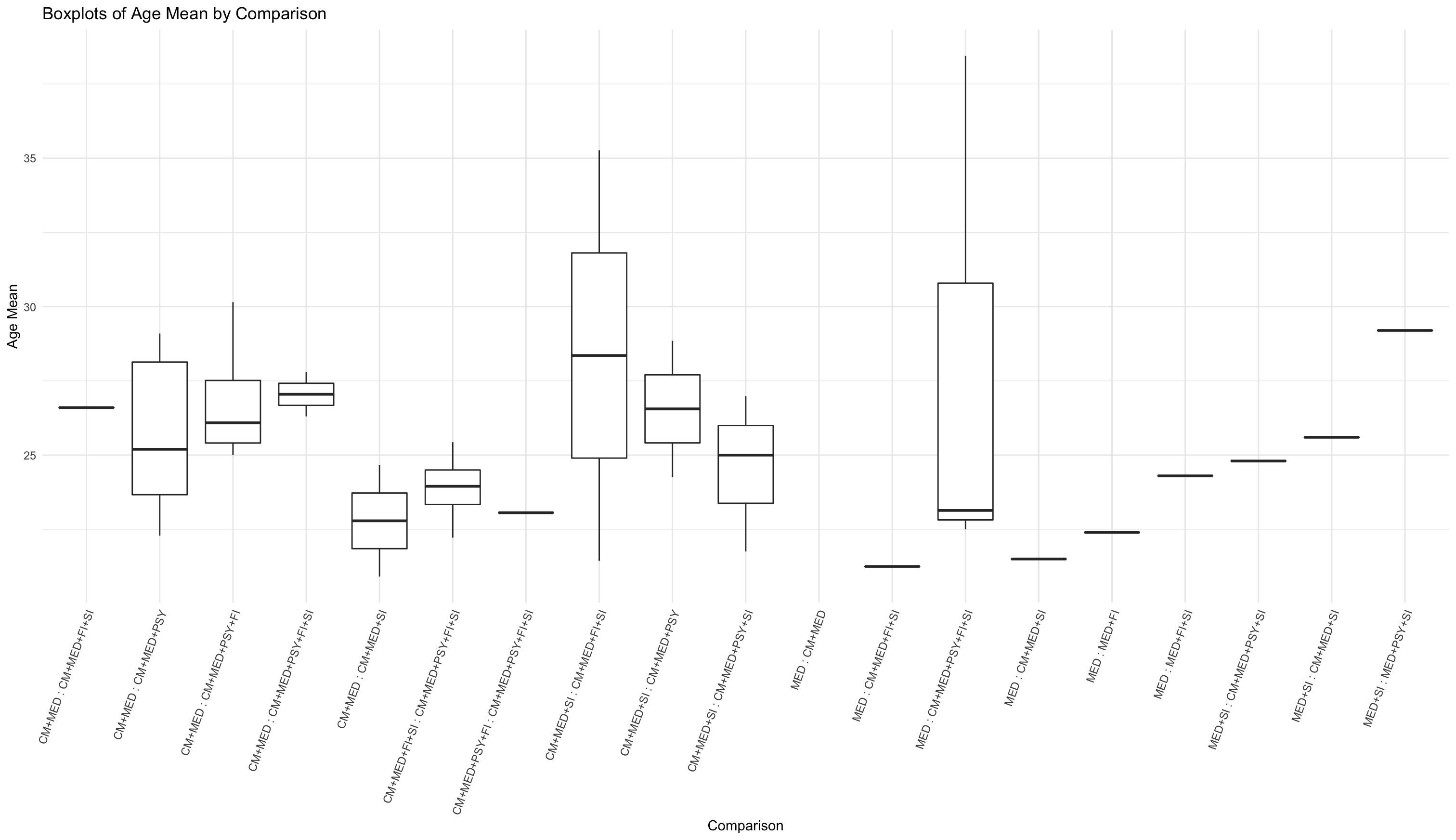


2. Gender – Percent Male


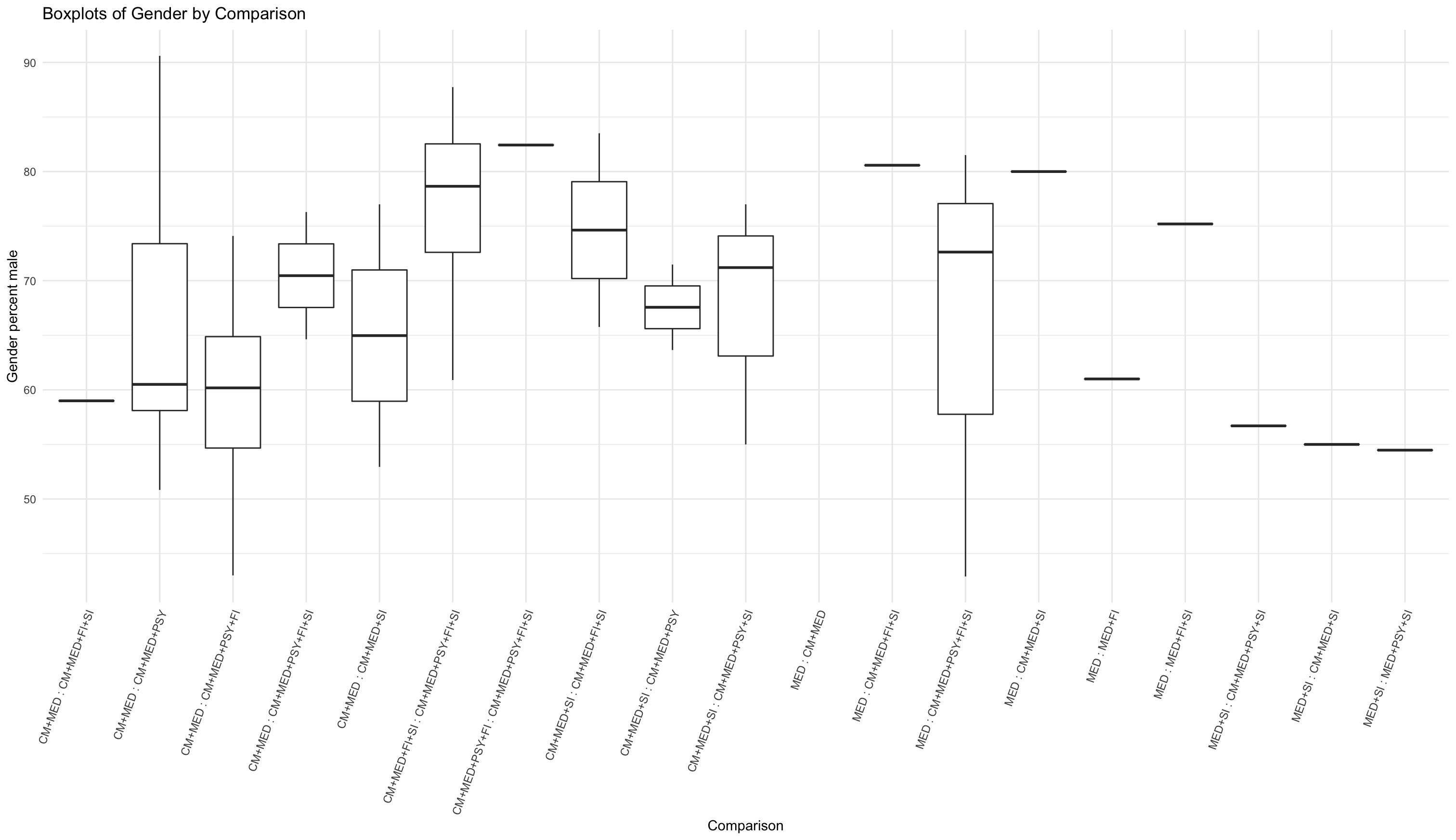


3. Publication Year


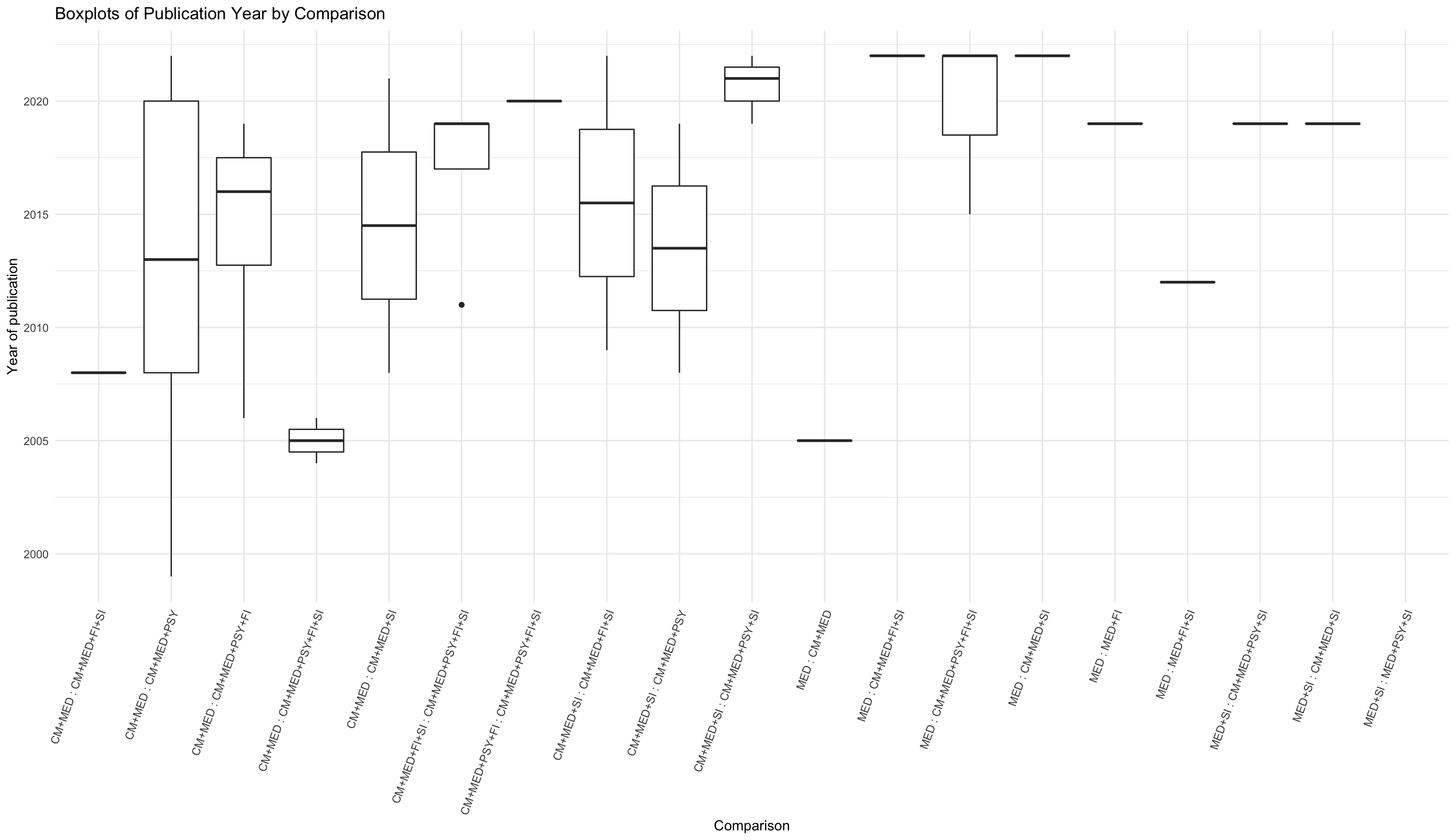


4. Duration of Intervention


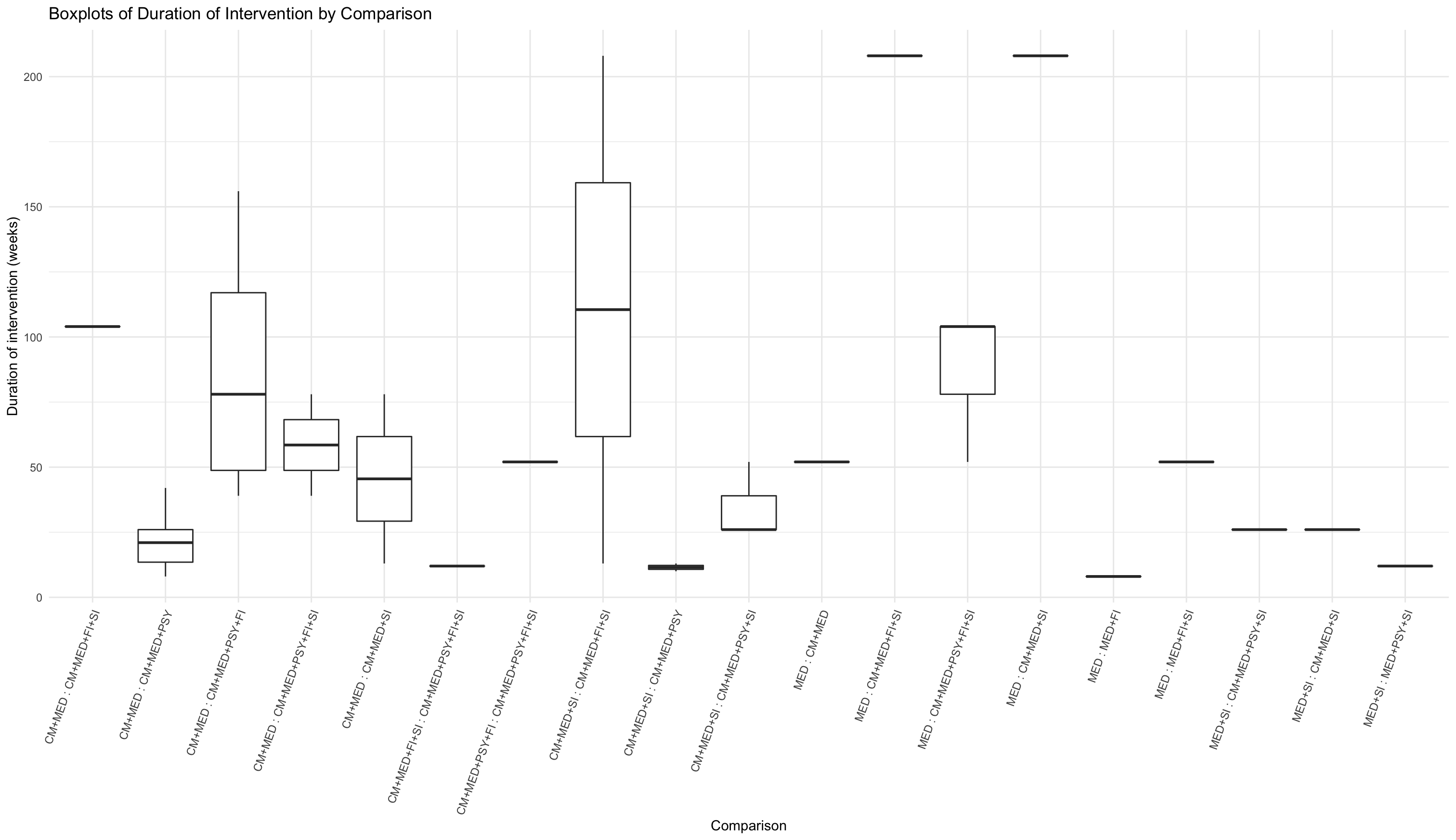


# ASSESSMENT OF NORMALITY

We assessed the normality assumption in the context of SMD meta-analysis by calculating mean/SD ratios for continuous primary outcomes in each intervention arm for included studies. Ratios less than 2 indicate skewness. Some cells are empty (.) because studies did not record data for that outcome (some studies recorded data for secondary outcomes only). Although there was some evidence of isolated skewed results, the overall distributions did not suggest violation of the normality assumption. Specifically, the number of arms that could be assumed normal based on a mean/sd ratio higher than 2 (i.e. a coefficient of variation equal or lower than 50%) were 4 out of 29 (86%) for positive psychotic symptoms at 3 months, 4 out of 25 (84%) for negative psychotic symptoms at 3 months, 5 out of 52 (90%) for positive psychotic symptoms at 1 year, and 7 out of 48 (85%) for negative psychotic symptoms at 1 year. We acknowledge that true normality of data can be verified only with access to individual participant data, an approach that would have introduced a number of additional advantages from the analytical point of view. Nonetheless, access to IPD often requires collaborative efforts and access to dedicated funding.

| **Study** | **Arm** | **Mean/ SD Ratio** | | | |
| --- | --- | --- | --- | --- | --- |
|  |  | **Positive Psychotic Symptoms (3 months)** | **Negative Psychotic Symptoms (3 months)** | **Positive Psychotic Symptoms (1 year)** | **Negative Psychotic Symptoms (1 year)** |
| Alvarez-Jiminez 2021^4^ | 1 | . | . | 3.24 | 2.78 |
|  | 2 | . | . | 2.99 | 2.59 |
| Bertelsen 2008^5^ | 1 | . | . | 1.62 | 2.08 |
|  | 2 | . | . | 1.61 | 2.27 |
| Cechnicki 2017^6^ | 1 | . | . | 4.83 | 2.74 |
|  | 2 | . | . | 2.94 | 1.98 |
| Chan 2009^7^ | 1 | 2.98 | . | 2.88 | . |
|  | 2 | 2.96 | . | 2.97 | . |
| Chien 2019^8^ | 1 | . | . | 6.90 | 3.25 |
|  | 2 | . | . | 8.79 | 3.30 |
|  | 3 | . | . | 8.78 | 2.66 |
| Chien 2019^9^ | 1 | 4.90 | 3.08 | 4.31 | 2.52 |
|  | 2 | 5.30 | 3.56 | 5.59 | 2.14 |
| Gafoor 2010^10^ | 1 | . | . | 3.36 | 2.33 |
|  | 2 | . | . | 4.15 | 2.74 |
| De Haan 2022^11^ | 1 | . | . | . | . |
|  | 2 | . | . | . | . |
|  | 3 | . | . | . | . |
| Drake 2014^12^ | 1 | . | . | 3.84 | . |
|  | 2 | . | . | 4.81 | . |
| Erickson 2020^13^ | 1 | . | . | . | . |
|  | 2 | . | . | . | . |
| Fan 2005^14^ | 1 | . | . | 5.13 | 2.35 |
|  | 2 | . | . | 2.78 | 7.34 |
| Gonzalez-Ortega 2021^15^ | 1 | . | 2.63 | . | 1.84 |
|  | 2 | . | 2.12 | . | 2.10 |
| Grawe 2006^16^ | 1 | . | . | . | . |
|  | 2 | . | . | . | . |
| Haddock 1999^17^ | 1 | 5.35 | . | . |  |
|  | 2 | 2.20 | . | . |  |
| Hui 2022^18^ | 1 | . | . | 3.47 | 2.59 |
|  | 2 | . | . | 2.95 | 2.65 |
| Jackson 2008^19^ | 1 | 1.83 | 2.73 | 1.76 | 2.34 |
|  | 2 | 1.90 | 2.77 | 2.08 | 2.32 |
| Jackson 2009^20^ | 1 | . | . | 2.10 | . |
|  | 2 | . | . | 2.30 | . |
| Kuipers 2004^21^ | 1 | 3.26 | 3.27 | 4.44 | 2.19 |
|  | 2 | 2.53 | 2.54 | 2.31 | 1.97 |
| Lecomte 2008^22^ | 1 | 3.81 | 1.61 | 3.88 | 1.84 |
|  | 2 | 2.41 | 2.22 | 3.65 | 2.02 |
|  | 3 | 3.04 | 1.45 | 2.37 | 2.64 |
| Linke 2019^23^ | 1 | 3.53 | 2.57 | . | . |
|  | 2 | 3.13 | 1.98 | . | . |
| Liu 2019^24^ | 1 | 4.60 | . | 5.48 | . |
|  | 2 | 5.76 | . | 3.74 | . |
| MacDougall 2019^25^ | 1 | 1.91 | 1.86 | . | . |
|  | 2 | 1.94 | 2.05 | . | . |
| Marchira 2019^26^ | 1 | 2.34 | . | . | . |
|  | 2 | 3.46 | . | . | . |
| Myin-Germeys 2022^27^ | 1 | . | . | 1.81 | 4.19 |
|  | 2 | . | . | 1.96 | 2.59 |
| Nuechterlein 2022^28^ | 1 | . | . | . | . |
|  | 2 | . | . | . | . |
| Palma 2019^29^ | 1 | . | . | 2.38 | 1.85 |
|  | 2 | . | . | 2.98 | 1.73 |
| Penn 2011^30^ | 1 | 3.75 | 3.87 | 3.42 | 3.63 |
|  | 2 | 4.01 | 2.99 | 4.22 | 3.59 |
| Pos 2019^31^ | 1 | . | 3.19 | . | 3.31 |
|  | 2 | . | 3.57 | . | 3.11 |
| Robinson 2022^32^ | 1 | 5.22 | 3.48 | 4.73 | 3.32 |
|  | 2 | 5.35 | 3.60 | 4.91 | 3.39 |
| Rocha 2020^33^ | 1 | . | . | 5.52 | 3.03 |
|  | 2 | . | . | 4.37 | 2.89 |
| Ruggeri 2015^34^ | 1 | . | . | 2.93 | 1.99 |
|  | 2 | . | . | 2.78 | 2.03 |
| Sonmez 2020^35^ | 1 | . | . | 2.03 | 2.87 |
|  | 2 | . | . | 2.68 | 2.55 |
| Srihari 2015^36^ | 1 | . | . | 3.51 | 2.10 |
|  | 2 | . | . | 3.46 | 2.22 |
| Uzenoff 2008^37^ | 1 | 3.59 | 2.72 | . | . |
|  | 2 | 2.79 | 2.01 | . | . |
| Valencia 2012^38^ | 1 | . | . | 4.19 | 2.67 |
|  | 2 | . | . | 3.56 | 2.32 |
| Van-Duin 2021^39^ | 1 | . | . | 3.73 | 3.15 |
|  | 2 | . | . | 3.93 | 2.46 |
| Vidarsdottir 2019^40^ | 1 | 2.63 | 2.84 | . | . |
|  | 2 | 2.92 | 3.51 | . | . |

# PAIRWISE META-ANALYSES/ NMA RESULTS SUMMARY

Full results for pairwise meta-analyses and NMAs including tests of statistical heterogeneity and inconsistency and network geometry are displayed by outcome in the sections below.

No interventions among the available comparisons were superior to pharmacotherapy alone at reducing positive or negative psychotic symptoms at 3 month follow-up, or dropouts by end of treatment.

There was suggestive evidence that the interventions ‘CM + MED’ (SMD, –0.72; 95% CI, –1.34 to –0.10; p = 0.022), ‘CM + MED + PSY’ (SMD, –0.89; 95% CI, –1.57 to –0.21; p = 0.010), ‘CM + MED + PSY + SI’ (SMD, –1.94; 95% CI, –3.05 to –0.82; p < 0.001) and ‘MED + FI + SI’ (SMD, –0.98; 95% CI, –1.77 to –0.18; p = 0.020) were superior to pharmacotherapy alone at reducing positive psychotic symptoms at 1 year.

There was suggestive evidence that ‘CM + MED’ (SMD, –2.65; 95% CI, –3.97 to –1.32; p < 0.001), ‘CM + MED + PSY’ (SMD, –3.21; 95% CI, –4.76 to –1.66; p < 0.001), ‘CM + MED + PSY + SI’ (SMD, –3.28; 95% CI, –5.82 to –0.75; p = 0.011), ‘CM + MED + SI’ (SMD, –2.80; 95% CI, –4.80 to –0.80; p = 0.006), ‘MED + PSY + SI’ (SMD, –3.34; 95% CI, –6.31 to –0.37; p = 0.028) and ‘MED + SI’ (SMD, –2.61; 95% CI, –5.14 to –0.08; p = 0.044) were superior to pharmacotherapy alone at reducing negative psychotic symptoms at 1 year.

There was suggestive evidence that ‘MED + FI + SI’ was superior to pharmacotherapy alone at improving social functioning at 1 year (SMD, 2.20; 95% CI, 1.29 to 3.11; p < 0.001).

# PRIMARY OUTCOME : POSITIVE SYMPTOMS (3 MONTHS)

## PAIRWISE AND NETWORK META-ANALYSIS

| **POSITIVE SYMPTOMS 3 MONTHS** | | | | | | | |
| --- | --- | --- | --- | --- | --- | --- | --- |
| **CM+MED** | . | 0.05  [-0.26 to 0.36] p = 0.76 | 0.04  [-0.83 to 0.91] p = 0.92 | -0.12  [-0.65 to 0.42] p = 0.67 | . | . | . |
| 0.21  [-0.35 to 0.76] p = 0.47 | **CM+MED+FI+SI** | . | -0.14  [-0.52 to 0.24] p = 0.46 | -0.35  [-0.82 to 0.11] p = 0.13 | . | . | . |
| 0.05  [-0.26 to 0.35] p = 0.76 | -0.16  [-0.73 to 0.41] p = 0.59 | **CM+MED+PSY** | . | -0.21  [-0.72 to 0.30] p = 0.43 | . | . | . |
| 0.06  [-0.52 to 0.64] p = 0.84 | -0.15  [-0.51 to 0.21] p = 0.43 | 0.01  [-0.60 to 0.62] p = 0.97 | **CM+MED+PSY+FI+SI** | . | 0.05  [-0.18 to 0.28] p = 0.67 | . | . |
| -0.14  [-0.60 to 0.31] p = 0.53 | -0.35  [-0.77 to 0.07] p = 0.11 | -0.19  [-0.65 to 0.27] p = 0.41 | -0.20  [-0.72 to 0.31] p = 0.44 | **CM+MED+SI** | . | . | . |
| 0.11  [-0.52 to 0.73] p = 0.73 | -0.10  [-0.52 to 0.33] p = 0.66 | 0.06  [-0.59 to 0.71] p = 0.85 | 0.05  [-0.18 to 0.28] p = 0.67 | 0.25  [-0.31 to 0.82] p = 0.38 | **MED** | **.** | . |
| ** | ** | ** | ** | ** | ** | **MED + PSY + SI** | -0.41  [-0.76 to -0.05] p = 0.008 |
| ** | ** | ** | ** | ** | ** | ** | **MED + SI** |

The lower triangle shows results from network meta-analyses (including direct and indirect evidence) in terms of SMDs for treatment in the column vs treatment in the row. Negative numbers favour the column-defining treatment vs the row-defining treatment.

The upper triangle shows results from pairwise meta-analyses for the treatment in the row vs the treatment in the column (direct evidence only). Negative numbers in the upper triangle favour the row-defining treatment vs the column-defining treatment. Some cells are empty (.) because there were no studies examining the corresponding comparison.

**For the network meta-analysis, it was not possible to compare ‘MED+PSY+SI’ and ‘MED+SI’ to other interventions because the only trial examining these interventions was disconnected from the network (see network plot, p. 35).

## EVALUATION OF INCONSISTENCY

We fit the NMA at the treatment level using the netmeta command in R. Statistical heterogeneity was estimated to be 𝜏2 = 0.02. The global, design-by-treatment test for inconsistency gave Q= 0.06, with 2 degrees of freedom, p-value= 0.97, I^2^ = 0% (95% uncertainty interval 0.0 to 70.8). The local approach to inconsistency (back-calculation method) gave the following results:

| comparison | k | prop | nma | direct | indir. | Diff | p-value |
| --- | --- | --- | --- | --- | --- | --- | --- |
| CM+MED:CM+MED+FI+SI | 0 | 0 | 0.21 | . | 0.21 | . | . |
| CM+MED:CM+MED+PSY | 4 | 0.98 | 0.05 | 0.05 | 0 | 0.05 | 0.96 |
| CM+MED:CM+MED+PSY+FI+SI | 1 | 0.45 | 0.06 | 0.04 | 0.07 | -0.03 | 0.96 |
| CM+MED:CM+MED+SI | 1 | 0.72 | -0.14 | -0.12 | -0.22 | 0.1 | 0.84 |
| CM+MED:MED | 0 | 0 | 0.11 | . | 0.11 | . | . |
| CM+MED+FI+SI:CM+MED+PSY | 0 | 0 | -0.16 | . | -0.16 | . | . |
| CM+MED+FI+SI:CM+MED+PSY+FI+SI | 3 | 0.89 | -0.15 | -0.14 | -0.17 | 0.03 | 0.96 |
| CM+MED+FI+SI:CM+MED+SI | 1 | 0.84 | -0.35 | -0.35 | -0.33 | -0.03 | 0.96 |
| CM+MED+FI+SI:MED | 0 | 0 | -0.1 | . | -0.1 | . | . |
| CM+MED+PSY:CM+MED+PSY+FI+SI | 0 | 0 | 0.01 | . | 0.01 | . | . |
| CM+MED+PSY:CM+MED+SI | 1 | 0.81 | -0.19 | -0.21 | -0.13 | -0.08 | 0.90 |
| CM+MED+PSY:MED | 0 | 0 | 0.06 | . | 0.06 | . | . |
| CM+MED+PSY+FI+SI:CM+MED+SI | 0 | 0 | -0.2 | . | -0.2 | . | . |
| CM+MED+PSY+FI+SI:MED | 1 | 1 | 0.05 | 0.05 | . | . | . |
| CM+MED+SI:MED | 0 | 0 | 0.25 | . | 0.25 | . | . |

## RANKING OF TREATMENTS

| Intervention | P Score |
| --- | --- |
| CM+MED+FI+SI | 0.78 |
| MED | 0.60 |
| CM+MED+PSY | 0.52 |
| CM+MED+PSY+FI+SI | 0.48 |
| CM+MED | 0.43 |
| CM+MED+SI | 0.19 |

## PUBLICATION BIAS/ SMALL STUDY EFFECTS

Funnel plotting and calculation of Egger’s test was not appropriate for this outcome due to small number of studies.

## NETWORK PLOT


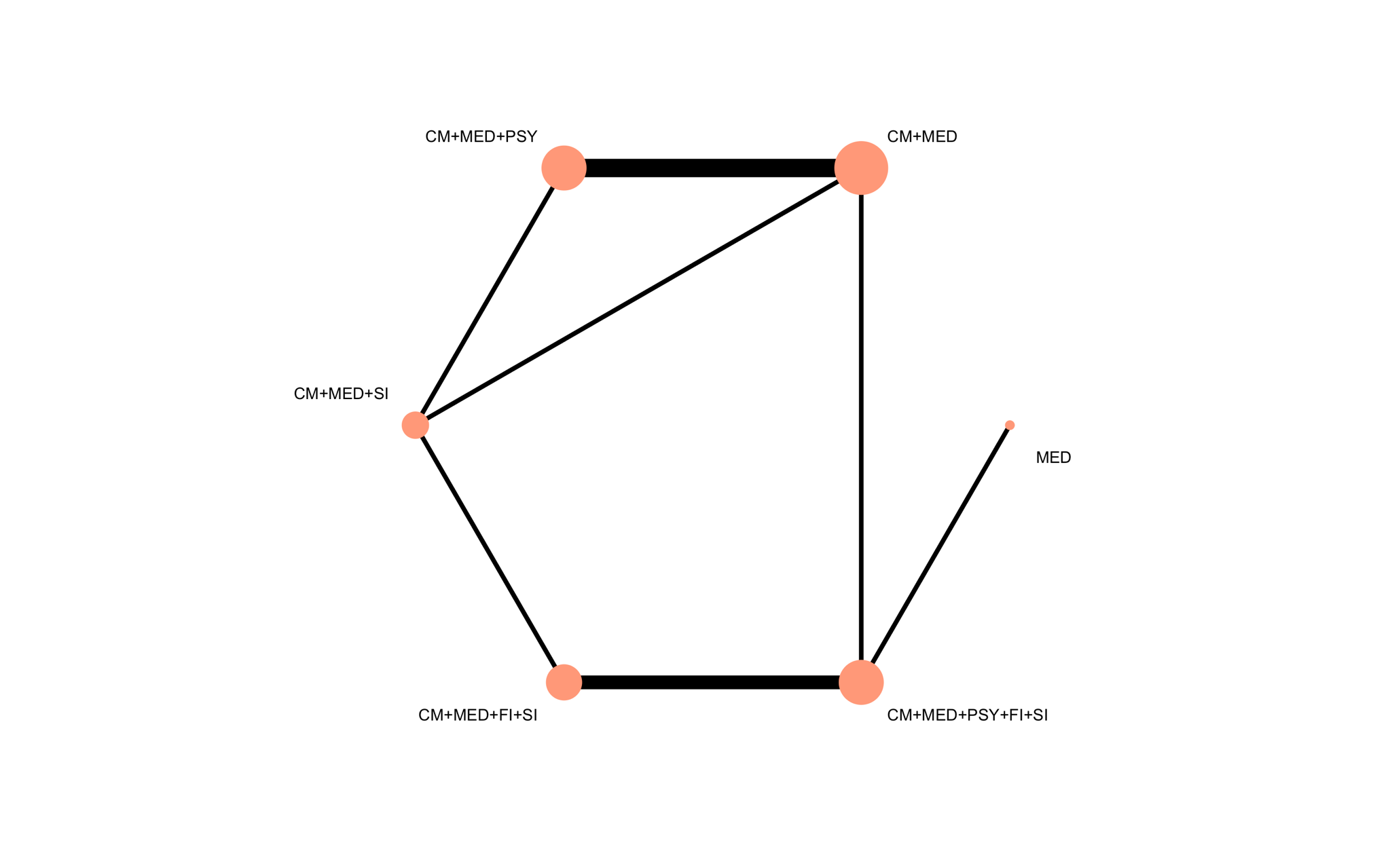

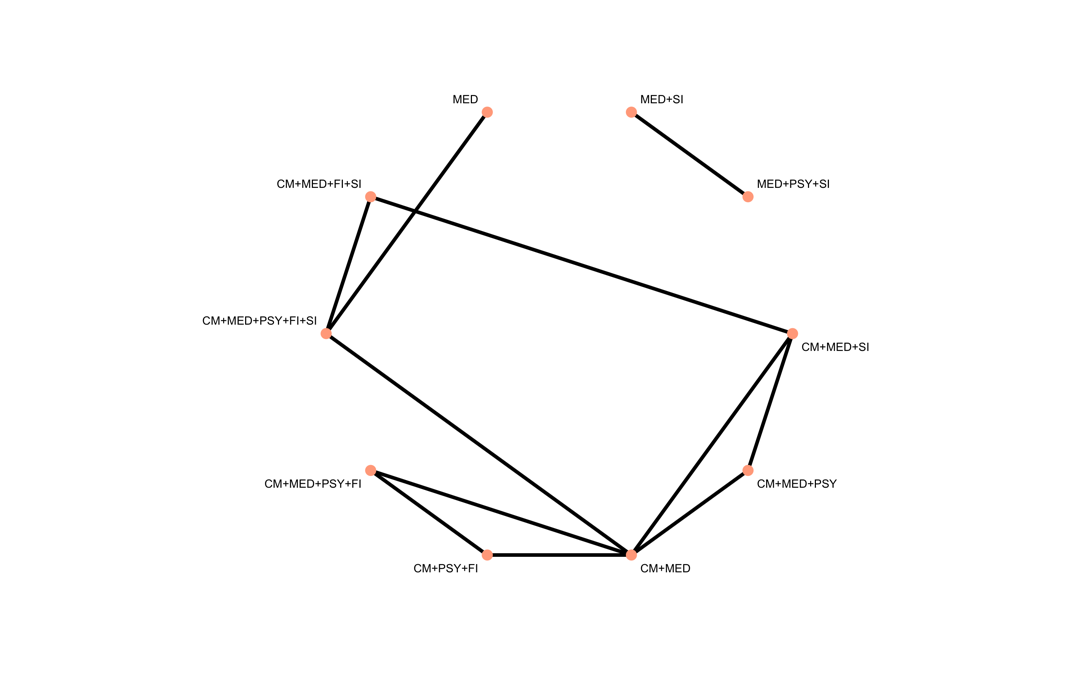


Nodes represent combinations of components, and lines denote trials performing the corresponding comparison. The width of the lines is proportional to the number of trials comparing each pair of treatments. The size of the nodes is proportional to the number of randomised participants.

# PRIMARY OUTCOME : NEGATIVE SYMPTOMS (3 MONTHS)

## PAIRWISE AND NETWORK META-ANALYSIS

| **NEGATIVE SYMPTOMS 3 MONTHS** | | | | | | | |
| --- | --- | --- | --- | --- | --- | --- | --- |
| **CM+MED** | . | 0.15  [-0.20 to 0.50] p = 0.39 | -0.15  [-1.04 to 0.75] p = 0.75 | 0.00  [-0.57 to 0.57] p = 1.00 | . | . | . |
| -0.32  [-1.27 to 0.62] p = 0.50 | **CM+MED+FI+SI** | . | 0.18  [-0.13 to 0.49] p = 0.26 | . | . | . | . |
| 0.15  [-0.20 to 0.50] p = 0.39 | 0.48  [-0.53 to 1.49] p = 0.35 | **CM+MED+PSY** | . | 0.09  [-0.45 to 0.64] p = 0.74 | . | . | . |
| -0.15  [-1.04 to 0.75] p = 0.75 | 0.18  [-0.13 to 0.49] p = 0.26 | -0.30  [-1.26 to 0.66] p = 0.54 | **CM+MED+PSY+FI+SI** | . | 0.07  [-0.24 to 0.38] p = 0.66 | . | . |
| 0.14  [-0.40 to 0.67] p = 0.62 | 0.46  [-0.63 to 1.55] p = 0.41 | -0.02  [-0.54 to 0.51] p = 0.95 | 0.28  [-0.76 to 1.32] p = 0.59 | **CM+MED+SI** | . | . | . |
| -0.08  [-1.02 to 0.87] p = 0.87 | 0.25  [-0.19 to 0.68] p = 0.27 | -0.23  [-1.24 to 0.78] p = 0.65 | 0.07  [-0.24 to 0.38] p = 0.66 | -0.21  [-1.30 to 0.87] p = 0.70 | **MED** | . | . |
| ** | ** | ** | ** | ** | ** | **MED + PSY + SI** | -0.45  [-0.83 to -0.07] p = 0.005 |
| ** | ** | ** | ** | ** | ** | ** | **MED + SI** |

The lower triangle shows results from network meta-analyses (including direct and indirect evidence) in terms of SMDs for treatment in the column vs treatment in the row. Negative numbers favour the column-defining treatment vs the row-defining treatment.

The upper triangle shows results from pairwise meta-analyses for the treatment in the row vs the treatment in the column (direct evidence only). Negative numbers in the upper triangle favour the row-defining treatment vs the column-defining treatment. Some cells are empty (.) because there were no studies examining the corresponding comparison

**For the network meta-analysis, it was not possible to compare ‘MED+PSY+SI’ and ‘MED+SI’ to other interventions because the only trial examining these interventions was disconnected from the network (see network plot, p. 40).

## EVALUATION OF INCONSISTENCY

We fit the NMA at the treatment level using the netmeta command in R. Statistical heterogeneity was estimated to be 𝜏2 = 0. The global, design-by-treatment test for inconsistency gave Q= 2.18, with 1 degree of freedom, p-value= 0.14, I^2^ = 10.4% (95% uncertainty interval 0.0 to 77.3). The local approach to inconsistency (back-calculation method) gave the following results:

| comparison | k | prop | nma | direct | indir. | Diff | p-value |
| --- | --- | --- | --- | --- | --- | --- | --- |
| CM+MED:CM+MED+FI+SI | 0 | 0 | -0.32 | . | -0.32 | . | . |
| CM+MED:CM+MED+PSY | 3 | 1 | 0.15 | 0.15 | . | . | . |
| CM+MED:CM+MED+PSY+FI+SI | 1 | 1 | -0.15 | -0.15 | . | . | . |
| CM+MED:CM+MED+SI | 1 | 0.88 | 0.14 | 0 | 1.16 | -1.16 | 0.17 |
| CM+MED:MED | 0 | 0 | -0.08 | . | -0.08 | . | . |
| CM+MED+FI+SI:CM+MED+PSY | 0 | 0 | 0.48 | . | 0.48 | . | . |
| CM+MED+FI+SI:CM+MED+PSY+FI+SI | 4 | 1 | 0.18 | 0.18 | . | . | . |
| CM+MED+FI+SI:CM+MED+SI | 0 | 0 | 0.46 | . | 0.46 | . | . |
| CM+MED+FI+SI:MED | 0 | 0 | 0.25 | . | 0.25 | . | . |
| CM+MED+PSY:CM+MED+PSY+FI+SI | 0 | 0 | -0.3 | . | -0.3 | . | . |
| CM+MED+PSY:CM+MED+SI | 1 | 0.91 | -0.02 | 0.09 | -1.21 | 1.3 | 0.17 |
| CM+MED+PSY:MED | 0 | 0 | -0.23 | . | -0.23 | . | . |
| CM+MED+PSY+FI+SI:CM+MED+SI | 0 | 0 | 0.28 | . | 0.28 | . | . |
| CM+MED+PSY+FI+SI:MED | 1 | 1 | 0.07 | 0.07 | . | . | . |
| CM+MED+SI:MED | 0 | 0 | -0.21 | . | -0.21 | . | . |

## RANKING OF TREATMENTS

| Intervention | P Score |
| --- | --- |
| CM+MED+PSY | 0.71 |
| CM+MED+SI | 0.66 |
| MED | 0.53 |
| CM+MED | 0.49 |
| CM+MED+PSY+FI+SI | 0.43 |
| CM+MED+FI+SI | 0.18 |

## PUBLICATION BIAS/ SMALL STUDY EFFECTS

Funnel plotting and calculation of Egger’s test was not appropriate for this outcome due to small number of studies.

## NETWORK PLOT


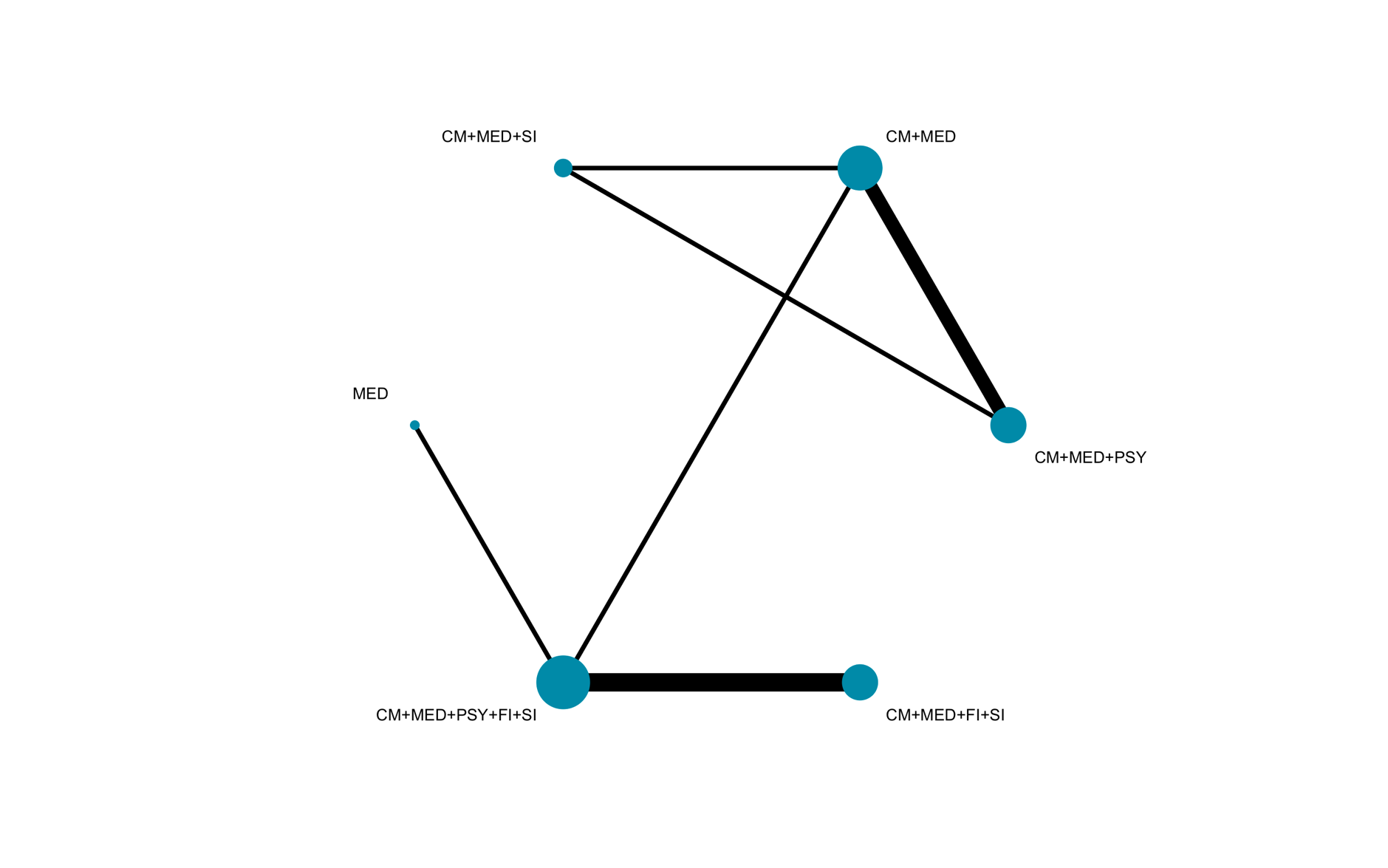

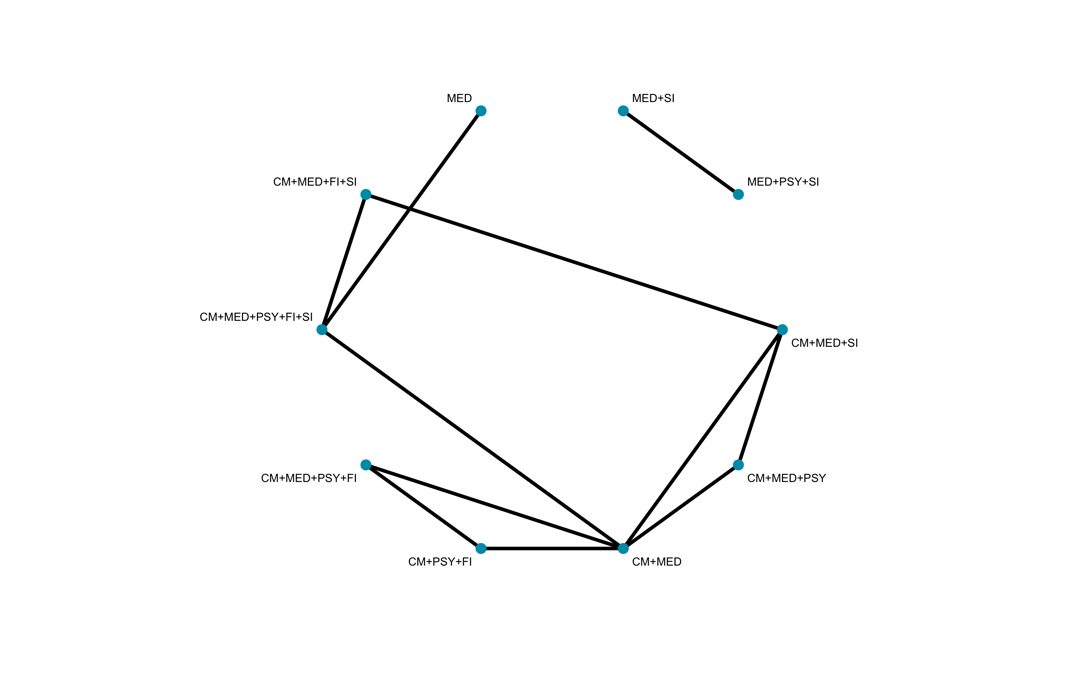


Nodes represent combinations of components, and lines denote trials performing the corresponding comparison. The width of the lines is proportional to the number of trials comparing each pair of treatments. The size of the nodes is proportional to the number of randomised participants.

# PRIMARY OUTCOME : DROPOUTS (END OF TREATMENT)

## PAIRWISE AND NETWORK META-ANALYSIS

| **DROPOUTS END OF TREATMENT** | | | | | | | | | | |
| --- | --- | --- | --- | --- | --- | --- | --- | --- | --- | --- |
| **CM+MED** | 1.56  [1.04 to 2.33] p = 0.030 | 0.95  [0.69 to 1.33] p = 0.78 | 0.99  [0.60 to 1.63] p = 0.97 | 1.24  [0.87 to 1.75] p = 0.23 | . | 1.22  [0.73 to 2.05] p = 0.45 | 0.71  [0.23 to 2.21] p = 0.56 | . | . | . |
| 1.42  [1.01 to 1.99] p = 0.046 | **CM+MED+FI+SI** | . | . | 1.03  [0.50 to 2.11] p = 0.94 | . | 0.96  [0.31 to 2.94] p = 0.94 | 0.82  [0.28 to 2.42] p = 0.72 | . | . | . |
| 0.94  [0.68 to 1.31] p = 0.72 | 0.66  [0.41 to 1.07] p = 0.09 | **CM+MED+PSY** | . | . | . | 0.84  [0.25 to 2.85] p = 0.78 | . | . | . | . |
| 1.14  [0.72 to 1.82] p = 0.58 | 0.81  [0.45 to 1.43] p = 0.46 | 1.21  [0.68 to 2.15] p = 0.51 | **CM+MED+PSY+FI** | 0.40  [0.11 to 1.47] p = 0.17 | . | . | . | . | . | . |
| 1.21  [0.91 to 1.63] p = 0.19 | 0.86  [0.58 to 1.27] p = 0.44 | 1.29  [0.83 to 2.00] p = 0.26 | 1.06  [0.63 to 1.81] p = 0.82 | **CM+MED+PSY+FI+SI** | . | . | 0.72  [0.52 to 0.98] p = 0.04 | . | . | . |
| 1.97  [0.79 to 4.93] p = 0.15 | 1.39  [0.53 to 3.65] p = 0.50 | 2.10  [0.80 to 5.52] p = 0.13 | 1.73  [0.62 to 4.83] p = 0.30 | 1.63  [0.63 to 4.20] p = 0.32 | **CM+MED+PSY+SI** | 0.58  [0.26 to 1.29] p = 0.18 | . | . | . | 1.00  [0.20 to 4.91] p = 1.00 |
| 1.15  [0.73 to 1.82] p = 0.54 | 0.81  [0.47 to 1.40] p = 0.46 | 1.23  [0.70 to 2.13] p = 0.47 | 1.01  [0.53 to 1.94] p = 0.98 | 0.95  [0.56 to 1.60] p = 0.85 | 0.58  [0.26 to 1.29] p = 0.18 | **CM+MED+SI** | 0.86  [0.29 to 2.53] p = 0.78 | . | . | 1.00  [0.20 to 4.91] p = 1.00 |
| 0.88  [0.60 to 1.29] p = 0.50 | 0.62  [0.39 to 0.98] p = 0.041 | 0.93  [0.56 to 1.54] p = 0.79 | 0.77  [0.43 to 1.38] p = 0.38 | 0.72  [0.54 to 0.97] p = 0.029 | 0.44  [0.17 to 1.17] p = 0.10 | 0.76  [0.43 to 1.33] p = 0.34 | **MED** | 2.00  [0.71 to 5.65] p = 0.19 | . | . |
| 1.75  [0.58 to 5.30] p = 0.32 | 1.24  [0.40 to 3.86] p = 0.71 | 1.86  [0.59 to 5.91] p = 0.29 | 1.54  [0.47 to 5.07] p = 0.48 | 1.45  [0.49 to 4.25] p = 0.50 | 0.89  [0.21 to 3.68] p = 0.87 | 1.52  [0.47 to 4.95] p = 0.49 | 2.00  [0.71 to 5.65] p = 0.19 | **MED+FI+SI** | . | . |
| 0.17  [0.01 to 4.48] p = 0.29 | 0.12  [0.00 to 3.20] p = 0.21 | 0.18  [0.01 to 4.83] p = 0.31 | 0.15  [0.01 to 4.05] p = 0.26 | 0.14  [0.01 to 3.72] p = 0.24 | 0.08  [0.00 to 2.20] p = 0.14 | 0.15  [0.01 to 3.76] p = 0.25 | 0.19  [0.01 to 5.18] p = 0.33 | 0.10  [0.00 to 3.04] p = 0.18 | **MED+PSY+SI** | 9.00  [0.49; 166.75] p = 0.14 |
| 1.51  [0.33 to 6.80] p = 0.59 | 1.07  [0.23 to 4.94] p = 0.94 | 1.60  [0.34 to 7.46] p = 0.55 | 1.32  [0.27 to 6.39] p = 0.73 | 1.24  [0.27 to 5.72] p = 0.78 | 0.76  [0.18 to 3.21] p = 0.71 | 1.31  [0.31 to 5.49] p = 0.71 | 1.72  [0.37 to 8.02] p = 0.49 | 0.86 0.87 [0.13 to 5.51] p = | 9.00  [0.49; 166.75] p = 0.14 | **MED+SI** |

The lower triangle shows results from network meta-analyses (including direct and indirect evidence) in terms of RRs for treatment in the column vs treatment in the row. Numbers less than 1 favour the column-defining treatment vs the row-defining treatment.

The upper triangle shows results from pairwise meta-analyses for the treatment in the row vs the treatment in the column (direct evidence only). Numbers less than 1 in the upper triangle favour the row-defining treatment vs the column-defining treatment. Some cells are empty (.) because there were no studies examining the corresponding comparison.

## EVALUATION OF INCONSISTENCY

We fit the NMA at the treatment level using the netmeta command in R. Statistical heterogeneity was estimated to be 𝜏2 = 0. The global, design-by-treatment test for inconsistency gave Q= 18.59, with 8 degrees of freedom, p-value = 0.02, I^2^ = 14.1% (95% uncertainty interval 0.0 to 46.1). The local approach to inconsistency (back-calculation method) gave the following results:

| comparison | k | prop | nma | direct | indir. | RoR | p-value |
| --- | --- | --- | --- | --- | --- | --- | --- |
| CM+MED:CM+MED+FI+SI | 1 | 0.67 | 1.68 | 1.93 | 1.26 | 1.52 | 0.42 |
| CM+MED:CM+MED+PSY | 11 | 0.99 | 0.93 | 0.96 | 0.09 | 10.74 | 0.28 |
| CM+MED:CM+MED+PSY+FI | 3 | 0.87 | 1.23 | 1 | 4.96 | 0.2 | 0.066 |
| CM+MED:CM+MED+PSY+FI+SI | 2 | 0.47 | 1.53 | 1.85 | 1.29 | 1.43 | 0.48 |
| CM+MED:CM+MED+PSY+SI | 0 | 0 | 2.27 | . | 2.27 | . | . |
| CM+MED:CM+MED+SI | 2 | 0.73 | 1.22 | 1.37 | 0.89 | 1.54 | 0.53 |
| CM+MED:MED | 1 | 0.19 | 0.89 | 0.68 | 0.94 | 0.73 | 0.66 |
| CM+MED:MED+FI+SI | 0 | 0 | 2.04 | . | 2.04 | . | . |
| CM+MED:MED+PSY+SI | 0 | 0 | 0.17 | . | 0.17 | . | . |
| CM+MED:MED+SI | 0 | 0 | 1.67 | . | 1.67 | . | . |
| CM+MED+FI+SI:CM+MED+PSY | 0 | 0 | 0.56 | . | 0.56 | . | . |
| CM+MED+FI+SI:CM+MED+PSY+FI | 0 | 0 | 0.73 | . | 0.73 | . | . |
| CM+MED+FI+SI:CM+MED+PSY+FI+SI | 4 | 0.39 | 0.91 | 1 | 0.86 | 1.16 | 0.80 |
| CM+MED+FI+SI:CM+MED+PSY+SI | 0 | 0 | 1.35 | . | 1.35 | . | . |
| CM+MED+FI+SI:CM+MED+SI | 1 | 0.31 | 0.73 | 0.95 | 0.65 | 1.47 | 0.62 |
| CM+MED+FI+SI:MED | 1 | 0.25 | 0.53 | 0.8 | 0.46 | 1.74 | 0.45 |
| CM+MED+FI+SI:MED+FI+SI | 0 | 0 | 1.21 | . | 1.21 | . | . |
| CM+MED+FI+SI:MED+PSY+SI | 0 | 0 | 0.1 | . | 0.1 | . | . |
| CM+MED+FI+SI:MED+SI | 0 | 0 | 0.99 | . | 0.99 | . | . |
| CM+MED+PSY:CM+MED+PSY+FI | 0 | 0 | 1.32 | . | 1.32 | . | . |
| CM+MED+PSY:CM+MED+PSY+FI+SI | 0 | 0 | 1.64 | . | 1.64 | . | . |
| CM+MED+PSY:CM+MED+PSY+SI | 0 | 0 | 2.43 | . | 2.43 | . | . |
| CM+MED+PSY:CM+MED+SI | 1 | 0.24 | 1.31 | 0.82 | 1.52 | 0.54 | 0.46 |
| CM+MED+PSY:MED | 0 | 0 | 0.95 | . | 0.95 | . | . |
| CM+MED+PSY:MED+FI+SI | 0 | 0 | 2.18 | . | 2.18 | . | . |
| CM+MED+PSY:MED+PSY+SI | 0 | 0 | 0.19 | . | 0.19 | . | . |
| CM+MED+PSY:MED+SI | 0 | 0 | 1.79 | . | 1.79 | . | . |
| CM+MED+PSY+FI:CM+MED+PSY+FI+SI | 1 | 0.23 | 1.24 | 0.36 | 1.8 | 0.2 | 0.066 |
| CM+MED+PSY+FI:CM+MED+PSY+SI | 0 | 0 | 1.84 | . | 1.84 | . | . |
| CM+MED+PSY+FI:CM+MED+SI | 0 | 0 | 0.99 | . | 0.99 | . | . |
| CM+MED+PSY+FI:MED | 0 | 0 | 0.72 | . | 0.72 | . | . |
| CM+MED+PSY+FI:MED+FI+SI | 0 | 0 | 1.65 | . | 1.65 | . | . |
| CM+MED+PSY+FI:MED+PSY+SI | 0 | 0 | 0.14 | . | 0.14 | . | . |
| CM+MED+PSY+FI:MED+SI | 0 | 0 | 1.35 | . | 1.35 | . | . |
| CM+MED+PSY+FI+SI:CM+MED+PSY+SI | 0 | 0 | 1.49 | . | 1.49 | . | . |
| CM+MED+PSY+FI+SI:CM+MED+SI | 0 | 0 | 0.8 | . | 0.8 | . | . |
| CM+MED+PSY+FI+SI:MED | 3 | 0.82 | 0.58 | 0.57 | 0.64 | 0.89 | 0.83 |
| CM+MED+PSY+FI+SI:MED+FI+SI | 0 | 0 | 1.33 | . | 1.33 | . | . |
| CM+MED+PSY+FI+SI:MED+PSY+SI | 0 | 0 | 0.11 | . | 0.11 | . | . |
| CM+MED+PSY+FI+SI:MED+SI | 0 | 0 | 1.09 | . | 1.09 | . | . |
| CM+MED+PSY+SI:CM+MED+SI | 3 | 1 | 0.54 | 0.54 | . | . | . |
| CM+MED+PSY+SI:MED | 0 | 0 | 0.39 | . | 0.39 | . | . |
| CM+MED+PSY+SI:MED+FI+SI | 0 | 0 | 0.9 | . | 0.9 | . | . |
| CM+MED+PSY+SI:MED+PSY+SI | 0 | 0 | 0.08 | . | 0.08 | . | . |
| CM+MED+PSY+SI:MED+SI | 1 | 0.82 | 0.73 | 1 | 0.17 | 5.87 | 0.39 |
| CM+MED+SI:MED | 1 | 0.36 | 0.73 | 0.84 | 0.67 | 1.26 | 0.77 |
| CM+MED+SI:MED+FI+SI | 0 | 0 | 1.67 | . | 1.67 | . | . |
| CM+MED+SI:MED+PSY+SI | 0 | 0 | 0.14 | . | 0.14 | . | . |
| CM+MED+SI:MED+SI | 1 | 0.82 | 1.36 | 1 | 5.87 | 0.17 | 0.39 |
| MED+FI+SI:MED | 1 | 1 | 0.44 | 0.44 | . | . | . |
| MED+PSY+SI:MED | 0 | 0 | 5.1 | . | 5.1 | . | . |
| MED+SI:MED | 0 | 0 | 0.53 | . | 0.53 | . | . |
| MED+FI+SI:MED+PSY+SI | 0 | 0 | 0.09 | . | 0.09 | . | . |
| MED+FI+SI:MED+SI | 0 | 0 | 0.82 | . | 0.82 | . | . |
| MED+PSY+SI:MED+SI | 1 | 1 | 9.57 | 9.57 | . | . | . |

## RANKING OF TREATMENTS

| Intervention | P Score |
| --- | --- |
| CM+MED+PSY+SI | 0.81 |
| MED+FI+SI | 0.73 |
| CM+MED+FI+SI | 0.73 |
| CM+MED+PSY+FI+SI | 0.67 |
| MED+SI | 0.63 |
| CM+MED+PSY+FI | 0.50 |
| CM+MED+SI | 0.48 |
| CM+MED | 0.32 |
| CM+MED+PSY | 0.28 |
| MED | 0.24 |
| MED+PSY+SI | 0.12 |

## PUBLICATION BIAS/ SMALL STUDY EFFECTS

We examined studies that compare an alternative treatment vs. ‘CM+MED’. Below we show the contour- adjusted funnel-plot. The funnel plot does not appear to be asymmetric. The p-value for Egger’s test is 0.9647. Overall, there is no clear evidence of publication bias and/or small study effects.


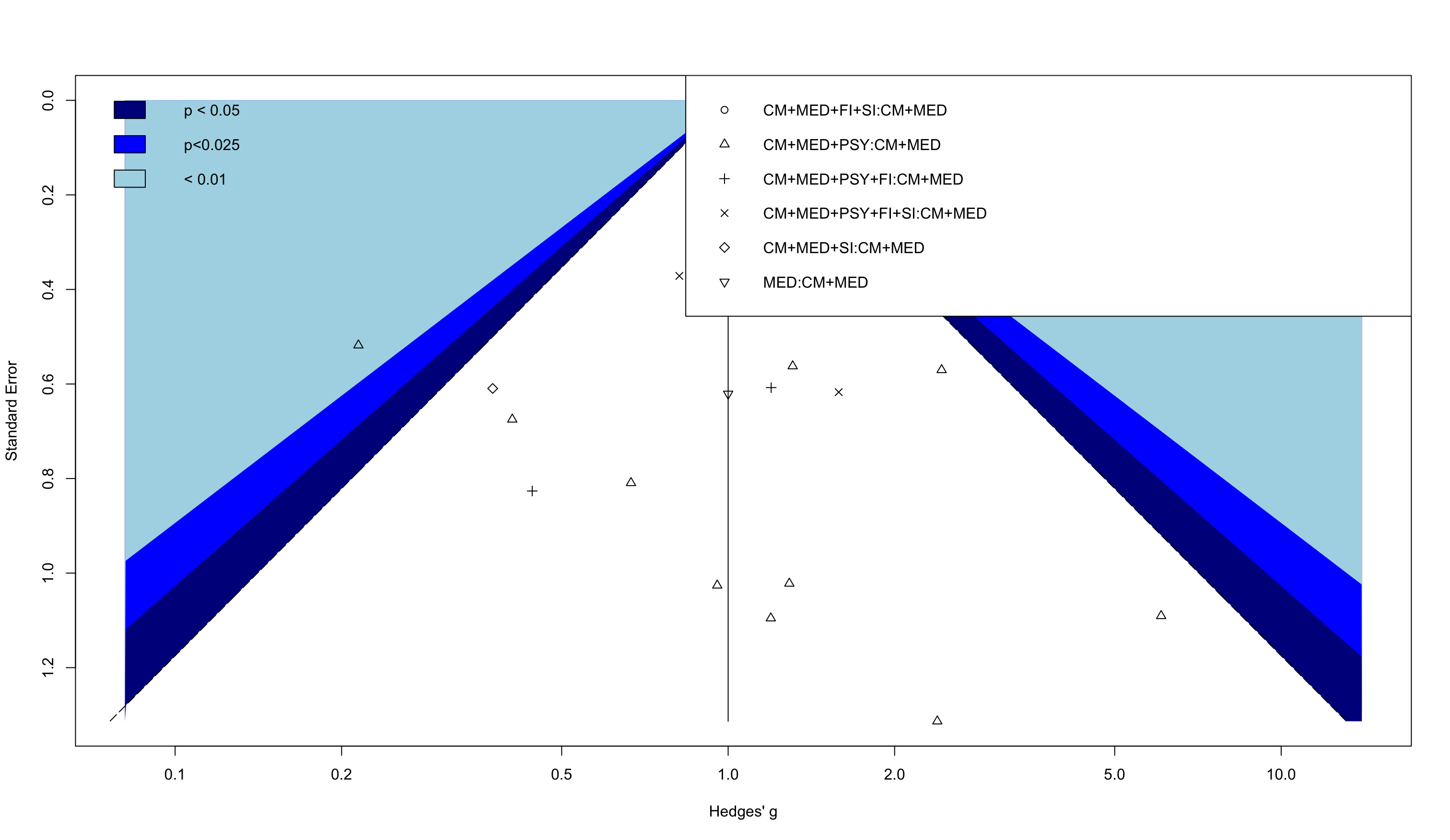


## NETWORK PLOT


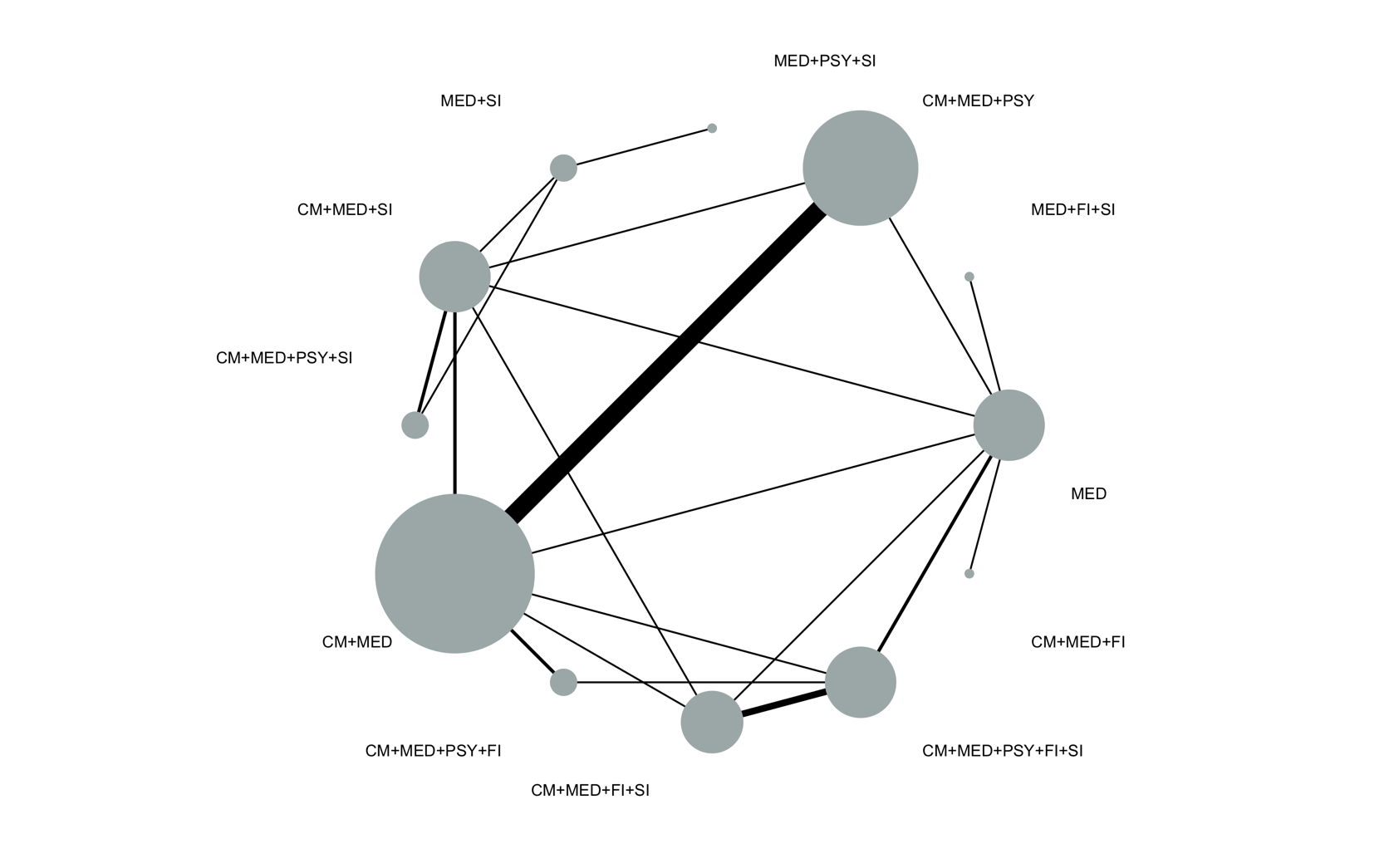


Nodes represent combinations of components, and lines denote trials performing the corresponding comparison. The width of the lines is proportional to the number of trials comparing each pair of treatments. The size of the nodes is proportional to the number of randomised participants.

# PRIMARY OUTCOME : POSITIVE SYMPTOMS (1 YEAR)

## PAIRWISE AND NETWORK META-ANALYSIS

| **POSITIVE SYMPTOMS 1 YEAR** | | | | | | | | | |
| --- | --- | --- | --- | --- | --- | --- | --- | --- | --- |
| **CM+MED** | . | 0.19  [-0.12 to 0.50] p = 0.23 | 0.17  [-1.06 to 1.40] p = 0.79 | . | -0.25  [-1.21 to 0.70] p = 0.61 | -1.09  [-1.85 to -0.32] p = 0.0052 | . | . | . |
| -0.18  [-0.99 to 0.62] p = 0.65 | **CM+MED+FI+SI** | . | 0.20  [-0.67 to 1.07] p = 0.65 | . | -0.58  [-1.36 to 0.20] p = 0.15 | . | . | . | . |
| 0.17  [-0.14 to 0.48] p = 0.28 | 0.36  [-0.48 to 1.19] p = 0.40 | **CM+MED+PSY** | . | . | -0.53  [-1.43 to 0.37] p = 0.25 | . | . | . | . |
| -0.25  [-0.90 to 0.40] p = 0.45 | -0.07  [-0.80 to 0.67] p = 0.86 | -0.42  [-1.13 to 0.28] p = 0.24 | **CM+MED+PSY+FI+SI** | . | . | -0.31  [-0.81 to 0.19] p = 0.23 | . | . | . |
| 1.22  [ 0.18 to 2.25] p = 0.021 | 1.40  [ 0.39 to 2.41] p = 0.0066 | 1.04  [ 0.00 to 2.08] p = 0.049 | 1.47  [ 0.37 to 2.56] p = 0.0088 | **CM+MED+PSY+SI** | -1.77  [-2.51 to -1.02] p < 0.001 | . | . | . | -3.36  [-4.16 to -2.56] p < 0.001 |
| -0.55  [-1.27 to 0.17] p = 0.13 | -0.37  [-1.05 to 0.32] p = 0.30 | -0.72  [-1.45 to 0.01] p = 0.051 | -0.30  [-1.11 to 0.51] p = 0.47 | -1.77  [-2.51 to -1.02] p = <0.001 | **CM+MED+SI** | . | . | . | -1.59  [-2.33 to -0.86] p < 0.001 |
| -0.72  [-1.34 to -0.10] p = 0.022 | -0.54  [-1.35 to 0.28] p = 0.20 | -0.89  [-1.57 to -0.21] p = 0.010 | -0.47  [-0.93 to -0.01] p = 0.047 | -1.94  [-3.05 to -0.82] p < 0.001 | -0.17  [-1.00 to 0.66] p = 0.69 | **MED** | 0.98  [ 0.18 to 1.77] p = 0.016 | . | . |
| 0.26  [-0.75 to 1.26] p = 0.62 | 0.44  [-0.69 to 1.58] p = 0.45 | 0.08  [-0.96 to 1.13] p = 0.87 | 0.51  [-0.41 to 1.43] p = 0.28 | -0.96  [-2.33 to 0.41] p = 0.17 | 0.81  [-0.34 to 1.96] p = 0.17 | 0.98  [ 0.18 to 1.77] p = 0.02 | **MED+FI+SI** | . | . |
| -0.73  [-2.00 to 0.54] p = 0.26 | -0.54  [-1.79 to 0.70] p = 0.39 | -0.90  [-2.17 to 0.37] p = 0.17 | -0.48  [-1.80 to 0.84] p = 0.48 | -1.94  [-3.03 to -0.86] p <0.001 | -0.18  [-1.22 to 0.87] p = 0.74 | -0.01  [-1.34 to 1.33] p = 0.99 | -0.99  [-2.54 to 0.57] p = 0.21 | **MED+PSY+SI** | -1.42  [-2.15 to -0.68] p < 0.001 |
| -2.15  [-3.18 to -1.11] p <0.001 | -1.96  [-2.97 to -0.95] p <0.001 | -2.32  [-3.35 to -1.28] p <0.001 | -1.89  [-2.99 to -0.80] p <0.001 | -3.36  [-4.16 to -2.56] p <0.001 | -1.59  [-2.33 to -0.86] p <0.001 | -1.43  [-2.54 to -0.31] p = 0.012 | -2.40  [-3.77 to -1.03] p <0.001 | -1.42  [-2.15 to -0.68] p <0.001 | **MED+SI** |

The lower triangle shows results from network meta-analyses (including direct and indirect evidence) in terms of SMDs for treatment in the column vs treatment in the row. Negative numbers favour the column-defining treatment vs the row-defining treatment.

The upper triangle shows results from pairwise meta-analyses for the treatment in the row vs the treatment in the column (direct evidence only). Negative numbers in the upper triangle favour the row-defining treatment vs the column-defining treatment. Some cells are empty (.) because there were no studies examining the corresponding comparison.

## EVALUATION OF INCONSISTENCY

We fit the NMA at the treatment level using the netmeta command in R. Statistical heterogeneity was estimated to be 𝜏2 = 0.09. The global, design-by-treatment test for inconsistency gave Q= 3.08, with 3 degrees of freedom, p-value= 0.38, I^2^ = 60.2% (95% uncertainty interval 20.2 to 80.1). The local approach to inconsistency (back-calculation method) gave the following results:

| comparison | k | prop | nma | direct | indir. | Diff | p-value |
| --- | --- | --- | --- | --- | --- | --- | --- |
| CM+MED:CM+MED+FI+SI | 0 | 0 | -0.18 | . | -0.18 | . | . |
| CM+MED:CM+MED+PSY | 7 | 0.99 | 0.17 | 0.19 | -1.41 | 1.6 | 0.26 |
| CM+MED:CM+MED+PSY+FI+SI | 1 | 0.28 | -0.25 | 0.17 | -0.42 | 0.59 | 0.43 |
| CM+MED:CM+MED+PSY+SI | 0 | 0 | 1.22 | . | 1.22 | . | . |
| CM+MED:CM+MED+SI | 1 | 0.56 | -0.55 | -0.25 | -0.94 | 0.68 | 0.35 |
| CM+MED:MED | 1 | 0.66 | -0.72 | -1.09 | -0.01 | -1.08 | 0.11 |
| CM+MED:MED+FI+SI | 0 | 0 | 0.26 | . | 0.26 | . | . |
| CM+MED:MED+PSY+SI | 0 | 0 | -0.73 | . | -0.73 | . | . |
| CM+MED:MED+SI | 0 | 0 | -2.15 | . | -2.15 | . | . |
| CM+MED+FI+SI:CM+MED+PSY | 0 | 0 | 0.36 | . | 0.36 | . | . |
| CM+MED+FI+SI:CM+MED+PSY+FI+SI | 1 | 0.71 | -0.07 | 0.2 | -0.72 | 0.92 | 0.26 |
| CM+MED+FI+SI:CM+MED+PSY+SI | 0 | 0 | 1.4 | . | 1.4 | . | . |
| CM+MED+FI+SI:CM+MED+SI | 1 | 0.77 | -0.37 | -0.58 | 0.34 | -0.92 | 0.26 |
| CM+MED+FI+SI:MED | 0 | 0 | -0.54 | . | -0.54 | . | . |
| CM+MED+FI+SI:MED+FI+SI | 0 | 0 | 0.44 | . | 0.44 | . | . |
| CM+MED+FI+SI:MED+PSY+SI | 0 | 0 | -0.54 | . | -0.54 | . | . |
| CM+MED+FI+SI:MED+SI | 0 | 0 | -1.96 | . | -1.96 | . | . |
| CM+MED+PSY:CM+MED+PSY+FI+SI | 0 | 0 | -0.42 | . | -0.42 | . | . |
| CM+MED+PSY:CM+MED+PSY+SI | 0 | 0 | 1.04 | . | 1.04 | . | . |
| CM+MED+PSY:CM+MED+SI | 1 | 0.66 | -0.72 | -0.53 | -1.09 | 0.56 | 0.47 |
| CM+MED+PSY:MED | 0 | 0 | -0.89 | . | -0.89 | . | . |
| CM+MED+PSY:MED+FI+SI | 0 | 0 | 0.08 | . | 0.08 | . | . |
| CM+MED+PSY:MED+PSY+SI | 0 | 0 | -0.9 | . | -0.9 | . | . |
| CM+MED+PSY:MED+SI | 0 | 0 | -2.32 | . | -2.32 | . | . |
| CM+MED+PSY+FI+SI:CM+MED+PSY+SI | 0 | 0 | 1.47 | . | 1.47 | . | . |
| CM+MED+PSY+FI+SI:CM+MED+SI | 0 | 0 | -0.3 | . | -0.3 | . | . |
| CM+MED+PSY+FI+SI:MED | 2 | 0.85 | -0.47 | -0.31 | -1.39 | 1.08 | 0.11 |
| CM+MED+PSY+FI+SI:MED+FI+SI | 0 | 0 | 0.51 | . | 0.51 | . | . |
| CM+MED+PSY+FI+SI:MED+PSY+SI | 0 | 0 | -0.48 | . | -0.48 | . | . |
| CM+MED+PSY+FI+SI:MED+SI | 0 | 0 | -1.89 | . | -1.89 | . | . |
| CM+MED+PSY+SI:CM+MED+SI | 1 | 1 | -1.77 | -1.77 | . | . | . |
| CM+MED+PSY+SI:MED | 0 | 0 | -1.94 | . | -1.94 | . | . |
| CM+MED+PSY+SI:MED+FI+SI | 0 | 0 | -0.96 | . | -0.96 | . | . |
| CM+MED+PSY+SI:MED+PSY+SI | 0 | 0 | -1.94 | . | -1.94 | . | . |
| CM+MED+PSY+SI:MED+SI | 1 | 1 | -3.36 | -3.36 | . | . | . |
| CM+MED+SI:MED | 0 | 0 | -0.17 | . | -0.17 | . | . |
| CM+MED+SI:MED+FI+SI | 0 | 0 | 0.81 | . | 0.81 | . | . |
| CM+MED+SI:MED+PSY+SI | 0 | 0 | -0.18 | . | -0.18 | . | . |
| CM+MED+SI:MED+SI | 1 | 1 | -1.59 | -1.59 | . | . | . |
| MED+FI+SI:MED | 1 | 1 | -0.98 | -0.98 | . | . | . |
| MED+PSY+SI:MED | 0 | 0 | 0.01 | . | 0.01 | . | . |
| MED+SI:MED | 0 | 0 | 1.43 | . | 1.43 | . | . |
| MED+FI+SI:MED+PSY+SI | 0 | 0 | -0.99 | . | -0.99 | . | . |
| MED+FI+SI:MED+SI | 0 | 0 | -2.4 | . | -2.4 | . | . |
| MED+PSY+SI:MED+SI | 1 | 1 | -1.42 | -1.42 | . | . | . |

## RANKING OF TREATMENTS

| Intervention | P Score |
| --- | --- |
| CM+MED+PSY+SI | 0.99 |
| CM+MED+PSY | 0.77 |
| MED+FI+SI | 0.75 |
| CM+MED | 0.63 |
| CM+MED+FI+SI | 0.54 |
| CM+MED+PSY+FI+SI | 0.49 |
| CM+MED+SI | 0.32 |
| MED+PSY+SI | 0.29 |
| MED | 0.22 |
| MED+SI | 0.00 |

## PUBLICATION BIAS/ SMALL STUDY EFFECTS

We examined studies that compare an alternative treatment vs. ‘CM+MED’. Below we show the contour- adjusted funnel-plot. The funnel plot does not appear to be asymmetric. The p-value for Egger’s test is 0.5276. Overall, there is no clear evidence of publication bias and/or small study effects.


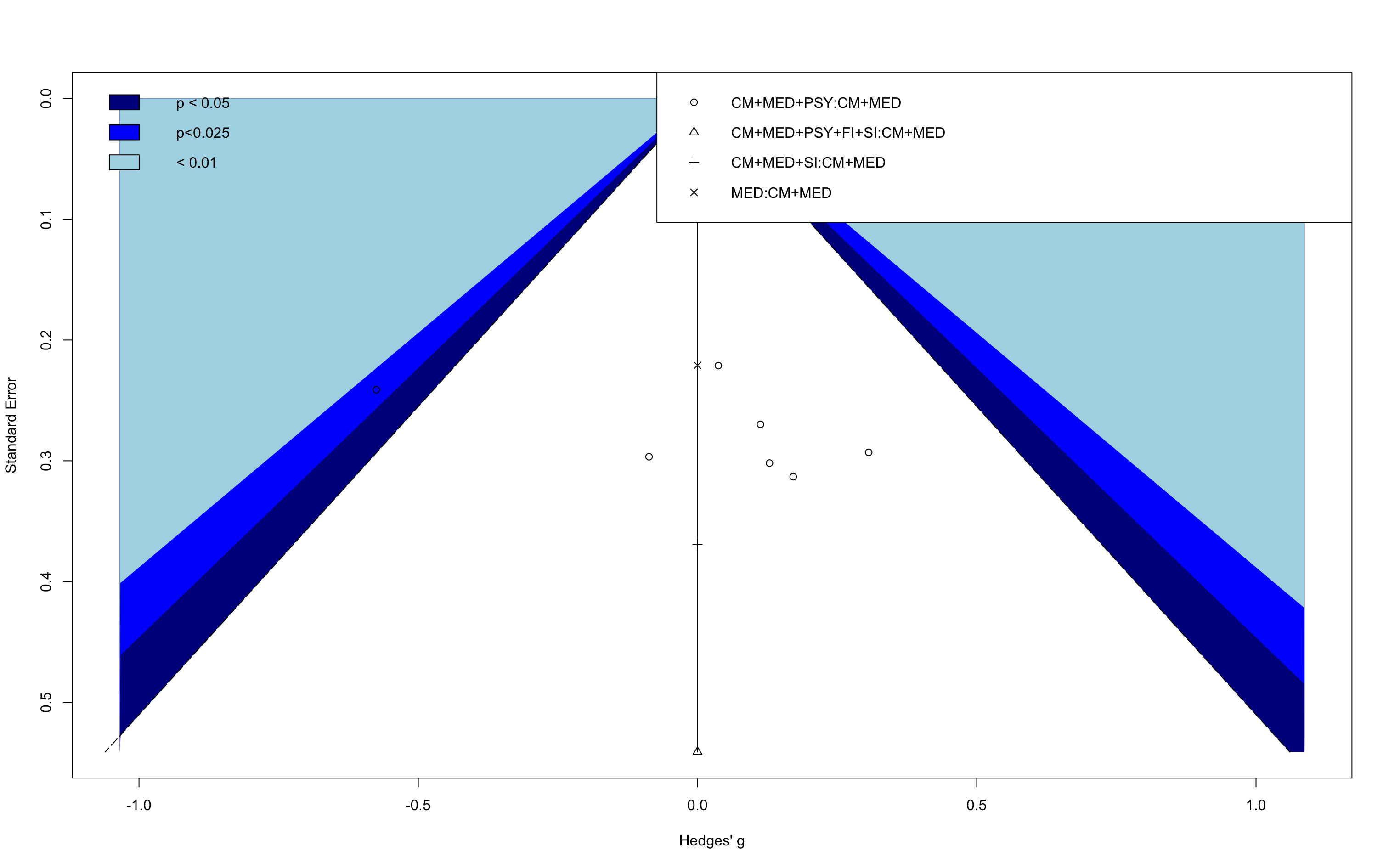


## NETWORK PLOT


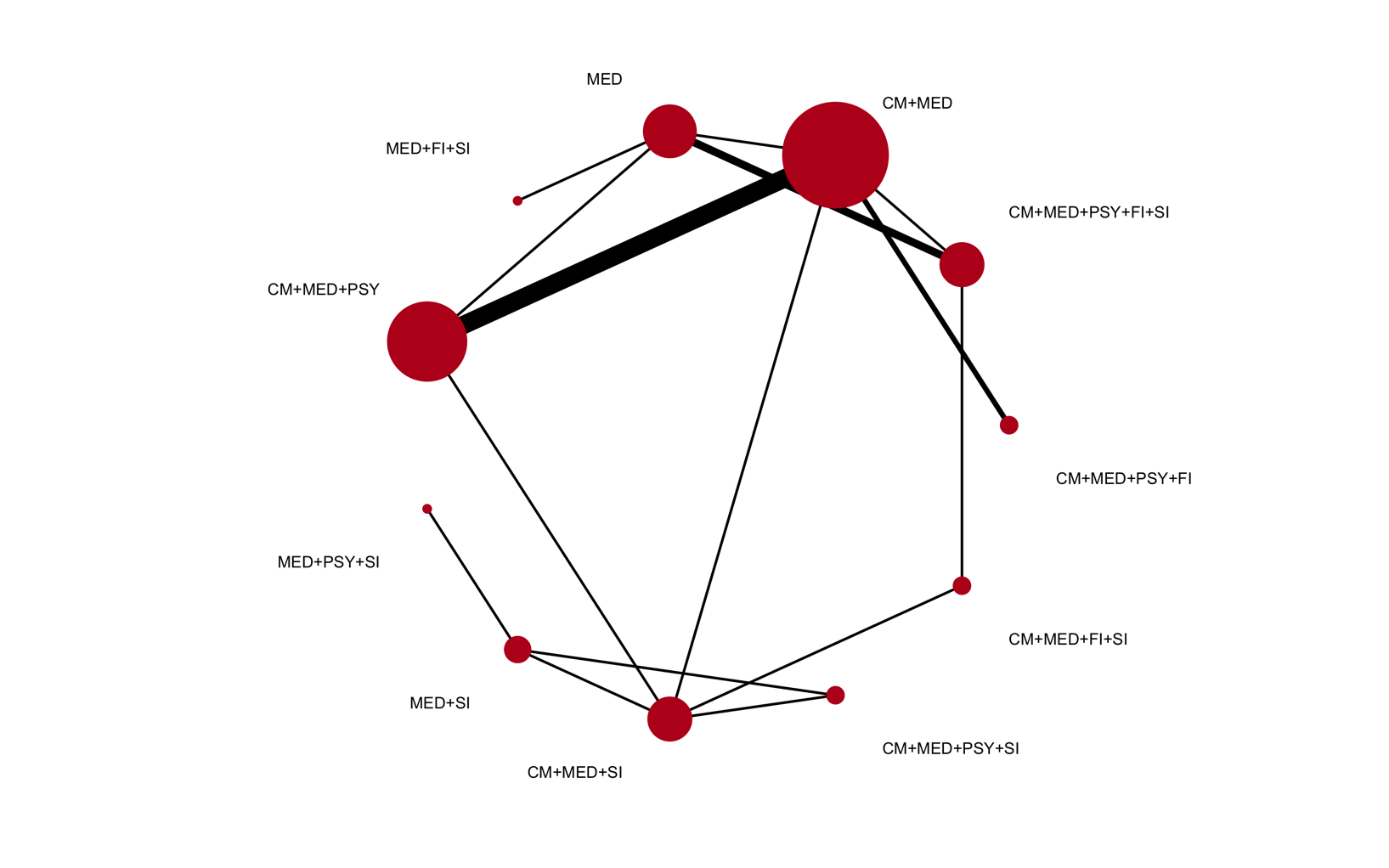


Nodes represent combinations of components, and lines denote trials performing the corresponding comparison. The width of the lines is proportional to the number of trials comparing each pair of treatments. The size of the nodes is proportional to the number of randomised participants.

# PRIMARY OUTCOME : NEGATIVE SYMPTOMS (1 YEAR)

## PAIRWISE AND NETWORK META-ANALYSIS

| **NEGATIVE SYMPTOMS 1 YEAR** | | | | | | | | | |
| --- | --- | --- | --- | --- | --- | --- | --- | --- | --- |
| **CM+MED** | . | 0.57  [-0.24 to 1.37] p = 0.17 | 0.12  [-1.73 to 1.96] p = 0.90 | . | -0.14  [-1.82 to 1.53] p = 0.87 | -4.27  [-5.95 to -2.59] p < 0.001 | . | . | . |
| -2.12  [-4.24 to 0.00] p = 0.050 | **CM+MED+FI+SI** | . | 0.27  [-1.36 to 1.90] p = 0.74 | . | . | . | . | . | . |
| 0.57  [-0.24 to 1.37] p = 0.17 | 2.69  [ 0.42 to 4.95] p = 0.020 | **CM+MED+PSY** | . | . | -0.14  [-1.78 to 1.50] p = 0.87 | . | . | . | . |
| -1.85  [-3.20 to -0.49] p = 0.008 | 0.27  [-1.36 to 1.90] p = 0.74 | -2.41  [-3.99 to -0.84] p = 0.003 | **CM+MED+PSY+FI+SI** | . | . | -0.11  [-1.20 to 0.99] p = 0.85 | . | . | . |
| 0.64  [-1.52 to 2.80] p = 0.56 | 2.76  [-0.27 to 5.79] p = 0.074 | 0.07  [-2.08 to 2.23] p = 0.95 | 2.49  [-0.07 to 5.04] p = 0.056 | **CM+MED+PSY+SI** | -0.49  [-2.04 to 1.07] p = 0.54 | . | . | . | -0.67  [-2.23 to 0.88] p = 0.40 |
| 0.15  [-1.35 to 1.66] p = 0.84 | 2.27  [-0.33 to 4.87] p = 0.087 | -0.41  [-1.91 to 1.08] p = 0.59 | 2.00  [-0.03 to 4.03] p = 0.053 | -0.49  [-2.04 to 1.07] p = 0.54 | **CM+MED+SI** | . | . | . | -0.19  [-1.74 to 1.36] p = 0.81 |
| -2.65  [-3.97 to -1.32] p < 0.001 | -0.53  [-2.44 to 1.39] p = 0.59 | -3.21  [-4.76 to -1.66] p < 0.001 | -0.80  [-1.80 to 0.20] p = 0.12 | -3.28  [-5.82 to -0.75] p = 0.011 | -2.80  [-4.80 to -0.80] p = 0.006 | **MED** | 0.81  [-0.77 to 2.40] p = 0.31 | . | . |
| -1.83  [-3.89 to 0.23] p = 0.082 | 0.29  [-2.19 to 2.77] p = 0.82 | -2.40  [-4.61 to -0.18] p = 0.034 | 0.02  [-1.86 to 1.89] p = 0.99 | -2.47  [-5.46 to 0.52] p = 0.11 | -1.98  [-4.54 to 0.57] p = 0.13 | 0.81  [-0.77 to 2.40] p = 0.31 | **MED+FI+SI** | . | . |
| 0.70  [-1.96 to 3.36] p = 0.61 | 2.82  [-0.59 to 6.22] p = 0.11 | 0.13  [-2.52 to 2.79] p = 0.92 | 2.54  [-0.44 to 5.53] p = 0.095 | 0.06  [-2.14 to 2.26] p = 0.96 | 0.54  [-1.65 to 2.74] p = 0.63 | 3.34  [ 0.37 to 6.31] p = 0.028 | 2.53  [-0.84 to 5.90] p = 0.14 | **MED+PSY+SI** | -0.73  [-2.29 to 0.82] p = 0.35 |
| -0.04  [-2.20 to 2.13] p = 0.97 | 2.08  [-0.94 to 5.11] p = 0.18 | -0.60  [-2.76 to 1.55] p = 0.59 | 1.81  [-0.74 to 4.36] p = 0.16 | -0.67  [-2.23 to 0.88] p = 0.40 | -0.19  [-1.74 to 1.36] p = 0.81 | 2.61  [ 0.08 to 5.14] p = 0.044 | 1.80  [-1.19 to 4.78] p = 0.24 | -0.73  [-2.29 to 0.82] p = 0.36 | **MED+SI** |

The lower triangle shows results from network meta-analyses (including direct and indirect evidence) in terms of SMDs for treatment in the column vs treatment in the row. Negative numbers favour the column-defining treatment vs the row-defining treatment.

The upper triangle shows results from pairwise meta-analyses for the treatment in the row vs the treatment in the column (direct evidence only). Negative numbers in the upper triangle favour the row-defining treatment vs the column-defining treatment. Some cells are empty (.) because there were no studies examining the corresponding comparison.

## EVALUATION OF INCONSISTENCY

We fit the NMA at the treatment level using the netmeta command in R. Statistical heterogeneity was estimated to be 𝜏2 = 0.02. The global, design-by-treatment test for inconsistency gave Q= 40.95, with 2 degrees of freedom, p-value<0.001, I^2^ = 90.3% (95% uncertainty interval 81.7 to 94.9). The local approach to inconsistency (back-calculation method) gave the following results:

| comparison | k | prop | nma | direct | indir. | Diff | p-value |
| --- | --- | --- | --- | --- | --- | --- | --- |
| CM+MED:CM+MED+FI+SI | 0 | 0 | -2.12 | . | -2.12 | . | . |
| CM+MED:CM+MED+PSY | 4 | 1 | 0.57 | 0.57 | . | . | . |
| CM+MED:CM+MED+PSY+FI+SI | 1 | 0.54 | -1.85 | 0.12 | -4.17 | 4.28 | 0.002 |
| CM+MED:CM+MED+PSY+SI | 0 | 0 | 0.64 | . | 0.64 | . | . |
| CM+MED:CM+MED+SI | 1 | 0.81 | 0.15 | -0.14 | 1.4 | -1.54 | 0.43 |
| CM+MED:MED | 1 | 0.62 | -2.65 | -4.27 | 0.01 | -4.28 | 0.002 |
| CM+MED:MED+FI+SI | 0 | 0 | -1.83 | . | -1.83 | . | . |
| CM+MED:MED+PSY+SI | 0 | 0 | 0.7 | . | 0.7 | . | . |
| CM+MED:MED+SI | 0 | 0 | -0.04 | . | -0.04 | . | . |
| CM+MED+FI+SI:CM+MED+PSY | 0 | 0 | 2.69 | . | 2.69 | . | . |
| CM+MED+FI+SI:CM+MED+PSY+FI+SI | 1 | 1 | 0.27 | 0.27 | . | . | . |
| CM+MED+FI+SI:CM+MED+PSY+SI | 0 | 0 | 2.76 | . | 2.76 | . | . |
| CM+MED+FI+SI:CM+MED+SI | 0 | 0 | 2.27 | . | 2.27 | . | . |
| CM+MED+FI+SI:MED | 0 | 0 | -0.53 | . | -0.53 | . | . |
| CM+MED+FI+SI:MED+FI+SI | 0 | 0 | 0.29 | . | 0.29 | . | . |
| CM+MED+FI+SI:MED+PSY+SI | 0 | 0 | 2.82 | . | 2.82 | . | . |
| CM+MED+FI+SI:MED+SI | 0 | 0 | 2.08 | . | 2.08 | . | . |
| CM+MED+PSY:CM+MED+PSY+FI+SI | 0 | 0 | -2.41 | . | -2.41 | . | . |
| CM+MED+PSY:CM+MED+PSY+SI | 0 | 0 | 0.07 | . | 0.07 | . | . |
| CM+MED+PSY:CM+MED+SI | 1 | 0.83 | -0.41 | -0.14 | -1.76 | 1.61 | 0.43 |
| CM+MED+PSY:MED | 0 | 0 | -3.21 | . | -3.21 | . | . |
| CM+MED+PSY:MED+FI+SI | 0 | 0 | -2.4 | . | -2.4 | . | . |
| CM+MED+PSY:MED+PSY+SI | 0 | 0 | 0.13 | . | 0.13 | . | . |
| CM+MED+PSY:MED+SI | 0 | 0 | -0.6 | . | -0.6 | . | . |
| CM+MED+PSY+FI+SI:CM+MED+PSY+SI | 0 | 0 | 2.49 | . | 2.49 | . | . |
| CM+MED+PSY+FI+SI:CM+MED+SI | 0 | 0 | 2 | . | 2 | . | . |
| CM+MED+PSY+FI+SI:MED | 2 | 0.84 | -0.8 | -0.11 | -4.39 | 4.28 | 0.002 |
| CM+MED+PSY+FI+SI:MED+FI+SI | 0 | 0 | 0.02 | . | 0.02 | . | . |
| CM+MED+PSY+FI+SI:MED+PSY+SI | 0 | 0 | 2.54 | . | 2.54 | . | . |
| CM+MED+PSY+FI+SI:MED+SI | 0 | 0 | 1.81 | . | 1.81 | . | . |
| CM+MED+PSY+SI:CM+MED+SI | 1 | 1 | -0.49 | -0.49 | . | . | . |
| CM+MED+PSY+SI:MED | 0 | 0 | -3.28 | . | -3.28 | . | . |
| CM+MED+PSY+SI:MED+FI+SI | 0 | 0 | -2.47 | . | -2.47 | . | . |
| CM+MED+PSY+SI:MED+PSY+SI | 0 | 0 | 0.06 | . | 0.06 | . | . |
| CM+MED+PSY+SI:MED+SI | 1 | 1 | -0.67 | -0.67 | . | . | . |
| CM+MED+SI:MED | 0 | 0 | -2.8 | . | -2.8 | . | . |
| CM+MED+SI:MED+FI+SI | 0 | 0 | -1.98 | . | -1.98 | . | . |
| CM+MED+SI:MED+PSY+SI | 0 | 0 | 0.54 | . | 0.54 | . | . |
| CM+MED+SI:MED+SI | 1 | 1 | -0.19 | -0.19 | . | . | . |
| MED+FI+SI:MED | 1 | 1 | -0.81 | -0.81 | . | . | . |
| MED+PSY+SI:MED | 0 | 0 | -3.34 | . | -3.34 | . | . |
| MED+SI:MED | 0 | 0 | -2.61 | . | -2.61 | . | . |
| MED+FI+SI:MED+PSY+SI | 0 | 0 | 2.53 | . | 2.53 | . | . |
| MED+FI+SI:MED+SI | 0 | 0 | 1.8 | . | 1.8 | . | . |
| MED+PSY+SI:MED+SI | 1 | 1 | -0.73 | -0.73 | . | . | . |

## RANKING OF TREATMENTS

| Intervention | P Score |
| --- | --- |
| CM+MED+PSY | 0.80 |
| CM+MED+PSY+SI | 0.79 |
| MED+PSY+SI | 0.79 |
| CM+MED+SI | 0.66 |
| CM+MED | 0.61 |
| MED+SI | 0.58 |
| MED+FI+SI | 0.26 |
| CM+MED+PSY+FI+SI | 0.25 |
| CM+MED+FI+SI | 0.19 |
| MED | 0.06 |

## PUBLICATION BIAS/ SMALL STUDY EFFECTS

Funnel plotting and calculation of Egger’s test was not appropriate for this outcome due to small number of studies.

## NETWORK PLOT


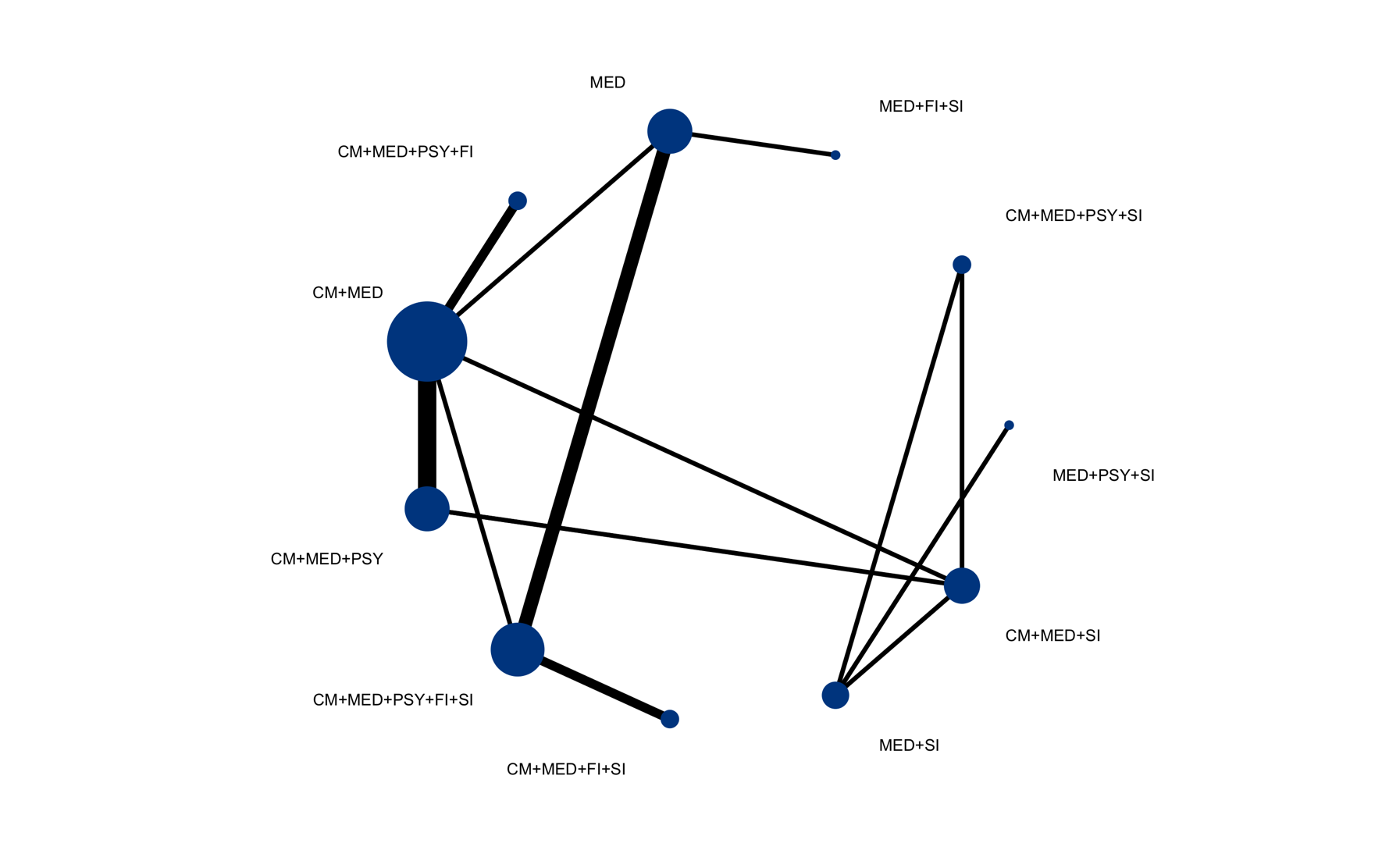


Nodes represent combinations of components, and lines denote trials performing the corresponding comparison. The width of the lines is proportional to the number of trials comparing each pair of treatments. The size of the nodes is proportional to the number of randomised participants.

# SECONDARY OUTCOME : DEPRESSIVE SYMPTOMS (1 YEAR)

## PAIRWISE AND NETWORK META-ANALYSIS

| **DEPRESSIVE SYMPTOMS 1 YEAR** | | | | |
| --- | --- | --- | --- | --- |
| **CM+MED** | . | -0.11  [-0.54 to 0.31] p = 0.59 | 0.14  [-0.92 to 1.20] p = 0.79 | . |
| 0.43  [-0.79 to 1.65] p = 0.49 | **CM+MED+FI+SI** | . | -0.29  [-0.90 to 0.32] p = 0.35 | . |
| -0.11  [-0.54 to 0.31] p = 0.59 | -0.54  [-1.84 to 0.75] p = 0.41 | **CM+MED+PSY** | . | . |
| 0.14  [-0.92 to 1.20] p = 0.79 | -0.29  [-0.90 to 0.32] p = 0.36 | 0.26  [-0.88 to 1.40] p = 0.66 | **CM+MED+PSY+FI+SI** | -0.15  [-0.39 to 0.10] p = 0.25 |
| 0.00  [-1.09 to 1.08] p = 0.99 | -0.43  [-1.09 to 0.22] p = 0.20 | 0.11  [-1.06 to 1.28] p = 0.85 | -0.15  [-0.39 to 0.10] p = 0.25 | **MED** |

The lower triangle shows results from network meta-analyses (including direct and indirect evidence) in terms of SMDs for treatment in the column vs treatment in the row. Negative numbers favour the column-defining treatment vs the row-defining treatment.

The upper triangle shows results from pairwise meta-analyses for the treatment in the row vs the treatment in the column (direct evidence only). Negative numbers in the upper triangle favour the row-defining treatment vs the column-defining treatment. Some cells are empty (.) because there were no studies examining the corresponding comparison.

## EVALUATION OF INCONSISTENCY

We fit the NMA at the treatment level using the netmeta command in R. Statistical heterogeneity was estimated to be 𝜏2 = 0.00. The global, design-by-treatment test for inconsistency gave Q= 0.56, with 1 degree of freedom, p-value= 0.46, I^2^ = 0.0%. The local approach to inconsistency (back-calculation method) gave the following results:

| comparison | k | prop | nma | direct | indir. | Diff | p-value |
| --- | --- | --- | --- | --- | --- | --- | --- |
| CM+MED:CM+MED+FI+SI | 0 | 0 | 0.43 | . | 0.43 | . | . |
| CM+MED:CM+MED+PSY | 2 | 1 | -0.11 | -0.11 | . | . | . |
| CM+MED:CM+MED+PSY+FI+SI | 1 | 1 | 0.14 | 0.14 | . | . | . |
| CM+MED:MED | 0 | 0 | 0 | . | 0 | . | . |
| CM+MED+FI+SI:CM+MED+PSY | 0 | 0 | -0.54 | . | -0.54 | . | . |
| CM+MED+FI+SI:CM+MED+PSY+FI+SI | 1 | 1 | -0.29 | -0.29 | . | . | . |
| CM+MED+FI+SI:MED | 0 | 0 | -0.43 | . | -0.43 | . | . |
| CM+MED+PSY:CM+MED+PSY+FI+SI | 0 | 0 | 0.26 | . | 0.26 | . | . |
| CM+MED+PSY:MED | 0 | 0 | 0.11 | . | 0.11 | . | . |
| CM+MED+PSY+FI+SI:MED | 1 | 1 | -0.15 | -0.15 | . | . | . |

## RANKING OF TREATMENTS

| Intervention | P Score |
| --- | --- |
| CM+MED+FI+SI | 0.82 |
| CM+MED+PSY+FI+SI | 0.58 |
| CM+MED | 0.46 |
| MED | 0.32 |
| CM+MED+PSY | 0.31 |

## PUBLICATION BIAS/ SMALL STUDY EFFECTS

Funnel plotting and calculation of Egger’s test was not appropriate for this outcome due to small number of studies.

## NETWORK PLOT


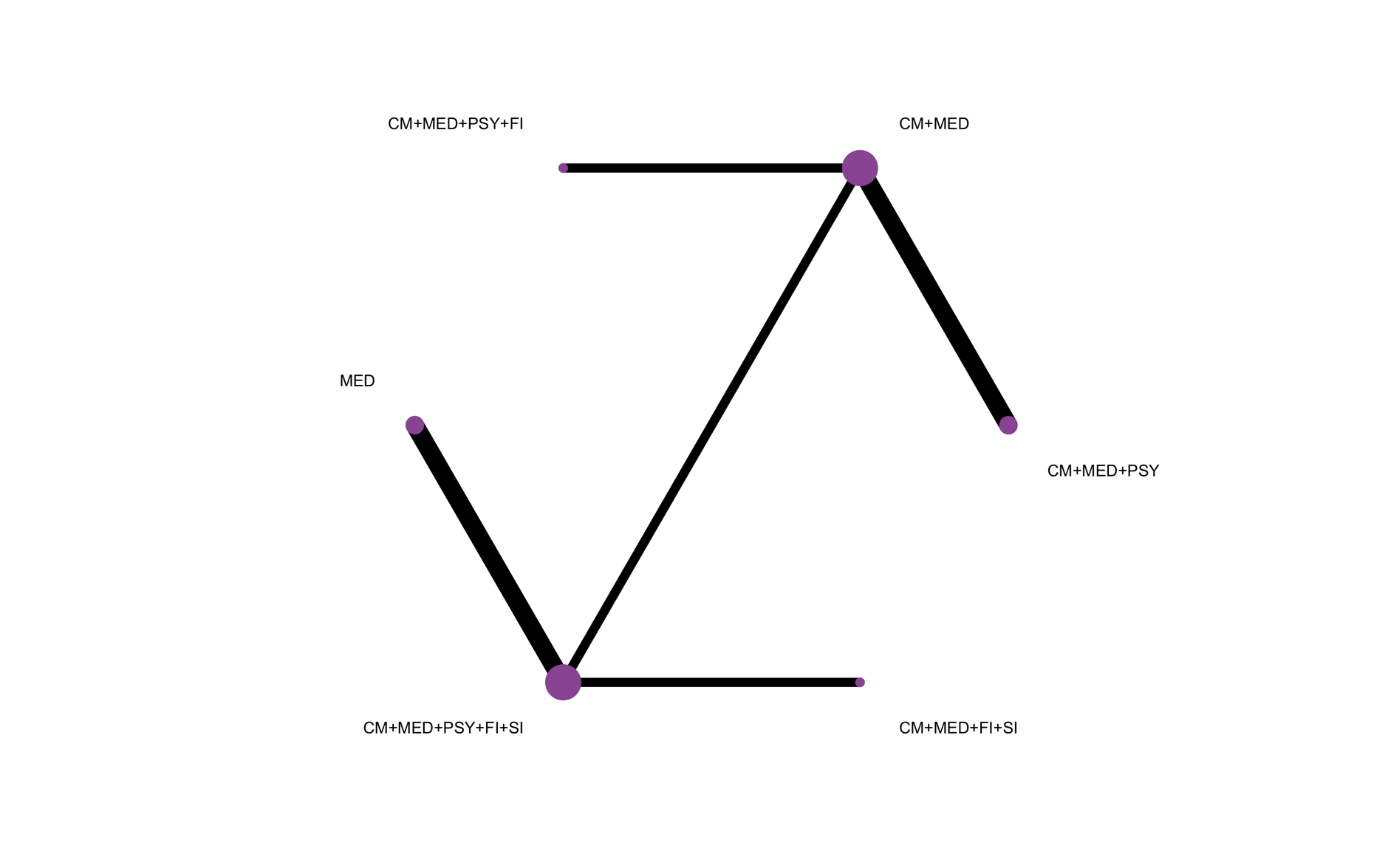


Nodes represent combinations of components, and lines denote trials performing the corresponding comparison. The width of the lines is proportional to the number of trials comparing each pair of treatments. The size of the nodes is proportional to the number of randomised participants.

# SECONDARY OUTCOME : SOCIAL FUNCTIONING (1 YEAR)

## PAIRWISE AND NETWORK META-ANALYSIS

| **SOCIAL FUNCTIONING 1 YEAR** | | | | | | | | | | |
| --- | --- | --- | --- | --- | --- | --- | --- | --- | --- | --- |
| **CM+MED** | . | -0.46  [-0.82 to -0.10] p = 0.012 | . | 0.56  [-0.73 to 1.84] p = 0.40 | . | -0.20  [-1.20 to 0.81] p = 0.70 | . | . | . | . |
| 0.77  [-0.81 to 2.36] p = 0.34 | **CM+MED+FI+SI** | . | . | -0.22  [-1.15 to 0.71] p = 0.64 | . | . | . | . | . | . |
| -0.46  [-0.82 to -0.10] p = 0.012 | -1.24  [-2.86 to 0.39] p = 0.14 | **CM+MED+PSY** | . | . | . | 0.45  [-0.50 to 1.41] p = 0.35 | . | . | . | . |
| 0.77  [-0.75 to 2.29] p = 0.32 | -0.00  [-1.23 to 1.23] p = 0.99 | 1.23  [-0.33 to 2.79] p = 0.12 | **CM+MED+PSY+FI** | -0.22  [-1.02 to 0.59] p = 0.60 | . | . | . | . | . | . |
| 0.56  [-0.73 to 1.84] p = 0.40 | -0.22  [-1.15 to 0.71] p = 0.64 | 1.02  [-0.32 to 2.35] p = 0.14 | -0.22  [-1.02 to 0.59] p = 0.60 | **CM+MED+PSY+FI+SI** | . | . | 0.02  [-0.73 to 0.77] p = 0.96 | . | . | . |
| -1.35  [-2.42 to -0.27] p = 0.014 | -2.12  [-4.04 to -0.20] p = 0.030 | -0.89  [-1.95 to 0.18] p = 0.10 | -2.12  [-3.98 to -0.26] p = 0.026 | -1.90  [-3.58 to -0.22] p = 0.026 | **CM+MED+PSY+SI** | 1.26  [ 0.65 to 1.87] p < 0.001 | . | . | . | 1.64  [ 0.83 to 2.44] p < 0.001 |
| -0.09  [-0.98 to 0.80] p = 0.85 | -0.86  [-2.68 to 0.96] p = 0.35 | 0.37  [-0.51 to 1.25] p = 0.41 | -0.86  [-2.62 to 0.90] p = 0.34 | -0.64  [-2.21 to 0.92] p = 0.42 | 1.26  [ 0.65 to 1.87] p < 0.001 | **CM+MED+SI** | . | . | . | 0.80  [ 0.01 to 1.60] p = 0.048 |
| 0.58  [-0.91 to 2.06] p = 0.45 | -0.20  [-1.39 to 0.99] p = 0.74 | 1.04  [-0.49 to 2.57] p = 0.19 | -0.19  [-1.29 to 0.90] p = 0.73 | 0.02  [-0.73 to 0.77] p = 0.96 | 1.92  [ 0.09 to 3.76] p = 0.040 | 0.66  [-1.07 to 2.40] p = 0.45 | **MED** | -2.20  [-3.11 to -1.29] p < 0.001 | . | . |
| -1.62  [-3.37 to 0.12] p = 0.068 | -2.40  [-3.90 to -0.90] p = 0.002 | -1.16  [-2.95 to 0.62] p = 0.20 | -2.39  [-3.82 to -0.97] p = 0.001 | -2.18  [-3.36 to -1.00] p < 0.001 | -0.28  [-2.33 to 1.77] p = 0.79 | -1.54  [-3.49 to 0.42] p = 0.13 | -2.20  [-3.11 to -1.29] p < 0.001 | **MED+FI+SI** | . | . |
| -0.48  [-1.89 to 0.93] p = 0.50 | -1.26  [-3.38 to 0.86] p = 0.25 | -0.02  [-1.43 to 1.38] p = 0.97 | -1.26  [-3.33 to 0.82] p = 0.24 | -1.04  [-2.95 to 0.87] p = 0.29 | 0.86  [-0.24 to 1.96] p = 0.13 | -0.40  [-1.49 to 0.70] p = 0.48 | -1.06  [-3.11 to 0.99] p = 0.31 | 1.14  [-1.11 to 3.38] p = 0.32 | **MED+PSY+SI** | 0.99  [ 0.20 to 1.79] p = 0.014 |
| 0.51  [-0.66 to 1.67] p = 0.39 | -0.27  [-2.24 to 1.70] p = 0.79 | 0.97  [-0.19 to 2.13] p = 0.10 | -0.26  [-2.18 to 1.65] p = 0.79 | -0.05  [-1.78 to 1.69] p = 0.96 | 1.86  [ 1.09 to 2.62] p < 0.001 | 0.60  [-0.16 to 1.35] p = 0.12 | -0.07  [-1.96 to 1.82] p = 0.94 | 2.13  [ 0.03 to 4.23] p = 0.047 | 0.99  [ 0.20 to 1.79] p = 0.014 | **MED+SI** |

The lower triangle shows results from network meta-analyses (including direct and indirect evidence) in terms of SMDs for treatment in the column vs treatment in the row. Positive numbers favour the column-defining treatment vs the row-defining treatment.

The upper triangle shows results from pairwise meta-analyses for the treatment in the row vs the treatment in the column (direct evidence only). Positive numbers in the upper triangle favour the row-defining treatment vs the column-defining treatment. Some cells are empty (.) because there were no studies examining the corresponding comparison.

## EVALUATION OF INCONSISTENCY

We fit the NMA at the treatment level using the netmeta command in R. Statistical heterogeneity was estimated to be 𝜏2 = 0.0816. The global, design-by-treatment test for inconsistency gave Q= 3.73, with 2 degree of freedom, p-value= 0.15, I^2^ = 64.2% (95% uncertainty interval 19.1 to 84.1). The local approach to inconsistency (back-calculation method) gave the following results:

| comparison | k | prop | nma | direct | indir. | Diff | p-value |
| --- | --- | --- | --- | --- | --- | --- | --- |
| CM+MED:CM+MED+FI+SI | 0 | 0 | 0.77 | . | 0.77 | . | . |
| CM+MED:CM+MED+PSY | 6 | 1 | -0.46 | -0.46 | . | . | . |
| CM+MED:CM+MED+PSY+FI | 0 | 0 | 0.77 | . | 0.77 | . | . |
| CM+MED:CM+MED+PSY+FI+SI | 1 | 1 | 0.56 | 0.56 | . | . | . |
| CM+MED:CM+MED+PSY+SI | 0 | 0 | -1.35 | . | -1.35 | . | . |
| CM+MED:CM+MED+SI | 1 | 0.78 | -0.09 | -0.2 | 0.28 | -0.48 | 0.66 |
| CM+MED:MED | 0 | 0 | 0.58 | . | 0.58 | . | . |
| CM+MED:MED+FI+SI | 0 | 0 | -1.62 | . | -1.62 | . | . |
| CM+MED:MED+PSY+SI | 0 | 0 | -0.48 | . | -0.48 | . | . |
| CM+MED:MED+SI | 0 | 0 | 0.51 | . | 0.51 | . | . |
| CM+MED+FI+SI:CM+MED+PSY | 0 | 0 | -1.24 | . | -1.24 | . | . |
| CM+MED+FI+SI:CM+MED+PSY+FI | 0 | 0 | 0 | . | 0 | . | . |
| CM+MED+FI+SI:CM+MED+PSY+FI+SI | 1 | 1 | -0.22 | -0.22 | . | . | . |
| CM+MED+FI+SI:CM+MED+PSY+SI | 0 | 0 | -2.12 | . | -2.12 | . | . |
| CM+MED+FI+SI:CM+MED+SI | 0 | 0 | -0.86 | . | -0.86 | . | . |
| CM+MED+FI+SI:MED | 0 | 0 | -0.2 | . | -0.2 | . | . |
| CM+MED+FI+SI:MED+FI+SI | 0 | 0 | -2.4 | . | -2.4 | . | . |
| CM+MED+FI+SI:MED+PSY+SI | 0 | 0 | -1.26 | . | -1.26 | . | . |
| CM+MED+FI+SI:MED+SI | 0 | 0 | -0.27 | . | -0.27 | . | . |
| CM+MED+PSY:CM+MED+PSY+FI | 0 | 0 | 1.23 | . | 1.23 | . | . |
| CM+MED+PSY:CM+MED+PSY+FI+SI | 0 | 0 | 1.02 | . | 1.02 | . | . |
| CM+MED+PSY:CM+MED+PSY+SI | 0 | 0 | -0.89 | . | -0.89 | . | . |
| CM+MED+PSY:CM+MED+SI | 1 | 0.85 | 0.37 | 0.45 | -0.1 | 0.55 | 0.66 |
| CM+MED+PSY:MED | 0 | 0 | 1.04 | . | 1.04 | . | . |
| CM+MED+PSY:MED+FI+SI | 0 | 0 | -1.16 | . | -1.16 | . | . |
| CM+MED+PSY:MED+PSY+SI | 0 | 0 | -0.02 | . | -0.02 | . | . |
| CM+MED+PSY:MED+SI | 0 | 0 | 0.97 | . | 0.97 | . | . |
| CM+MED+PSY+FI:CM+MED+PSY+FI+SI | 1 | 1 | -0.22 | -0.22 | . | . | . |
| CM+MED+PSY+FI:CM+MED+PSY+SI | 0 | 0 | -2.12 | . | -2.12 | . | . |
| CM+MED+PSY+FI:CM+MED+SI | 0 | 0 | -0.86 | . | -0.86 | . | . |
| CM+MED+PSY+FI:MED | 0 | 0 | -0.19 | . | -0.19 | . | . |
| CM+MED+PSY+FI:MED+FI+SI | 0 | 0 | -2.39 | . | -2.39 | . | . |
| CM+MED+PSY+FI:MED+PSY+SI | 0 | 0 | -1.26 | . | -1.26 | . | . |
| CM+MED+PSY+FI:MED+SI | 0 | 0 | -0.26 | . | -0.26 | . | . |
| CM+MED+PSY+FI+SI:CM+MED+PSY+SI | 0 | 0 | -1.9 | . | -1.9 | . | . |
| CM+MED+PSY+FI+SI:CM+MED+SI | 0 | 0 | -0.64 | . | -0.64 | . | . |
| CM+MED+PSY+FI+SI:MED | 1 | 1 | 0.02 | 0.02 | . | . | . |
| CM+MED+PSY+FI+SI:MED+FI+SI | 0 | 0 | -2.18 | . | -2.18 | . | . |
| CM+MED+PSY+FI+SI:MED+PSY+SI | 0 | 0 | -1.04 | . | -1.04 | . | . |
| CM+MED+PSY+FI+SI:MED+SI | 0 | 0 | -0.05 | . | -0.05 | . | . |
| CM+MED+PSY+SI:CM+MED+SI | 2 | 1 | 1.26 | 1.26 | . | . | . |
| CM+MED+PSY+SI:MED | 0 | 0 | 1.92 | . | 1.92 | . | . |
| CM+MED+PSY+SI:MED+FI+SI | 0 | 0 | -0.28 | . | -0.28 | . | . |
| CM+MED+PSY+SI:MED+PSY+SI | 0 | 0 | 0.86 | . | 0.86 | . | . |
| CM+MED+PSY+SI:MED+SI | 1 | 0.89 | 1.86 | 1.64 | 3.68 | -2.05 | 0.10 |
| CM+MED+SI:MED | 0 | 0 | 0.66 | . | 0.66 | . | . |
| CM+MED+SI:MED+FI+SI | 0 | 0 | -1.54 | . | -1.54 | . | . |
| CM+MED+SI:MED+PSY+SI | 0 | 0 | -0.4 | . | -0.4 | . | . |
| CM+MED+SI:MED+SI | 1 | 0.9 | 0.6 | 0.8 | -1.32 | 2.12 | 0.10 |
| MED+FI+SI:MED | 1 | 1 | 2.2 | 2.2 | . | . | . |
| MED+PSY+SI:MED | 0 | 0 | 1.06 | . | 1.06 | . | . |
| MED+SI:MED | 0 | 0 | 0.07 | . | 0.07 | . | . |
| MED+FI+SI:MED+PSY+SI | 0 | 0 | 1.14 | . | 1.14 | . | . |
| MED+FI+SI:MED+SI | 0 | 0 | 2.13 | . | 2.13 | . | . |
| MED+PSY+SI:MED+SI | 1 | 1 | 0.99 | 0.99 | . | . | . |

## RANKING OF TREATMENTS

| Intervention | P Score |
| --- | --- |
| MED+FI+SI | 0.92 |
| CM+MED+PSY+SI | 0.92 |
| CM+MED+PSY | 0.71 |
| MED+PSY+SI | 0.67 |
| CM+MED+SI | 0.52 |
| CM+MED | 0.48 |
| CM+MED+PSY+FI+SI | 0.30 |
| MED | 0.29 |
| MED+SI | 0.26 |
| CM+MED+FI+SI | 0.21 |
| CM+MED+PSY+FI | 0.21 |

## PUBLICATION BIAS/ SMALL STUDY EFFECTS

Funnel plotting and calculation of Egger’s test was not appropriate for this outcome due to small number of studies.

## NETWORK PLOT


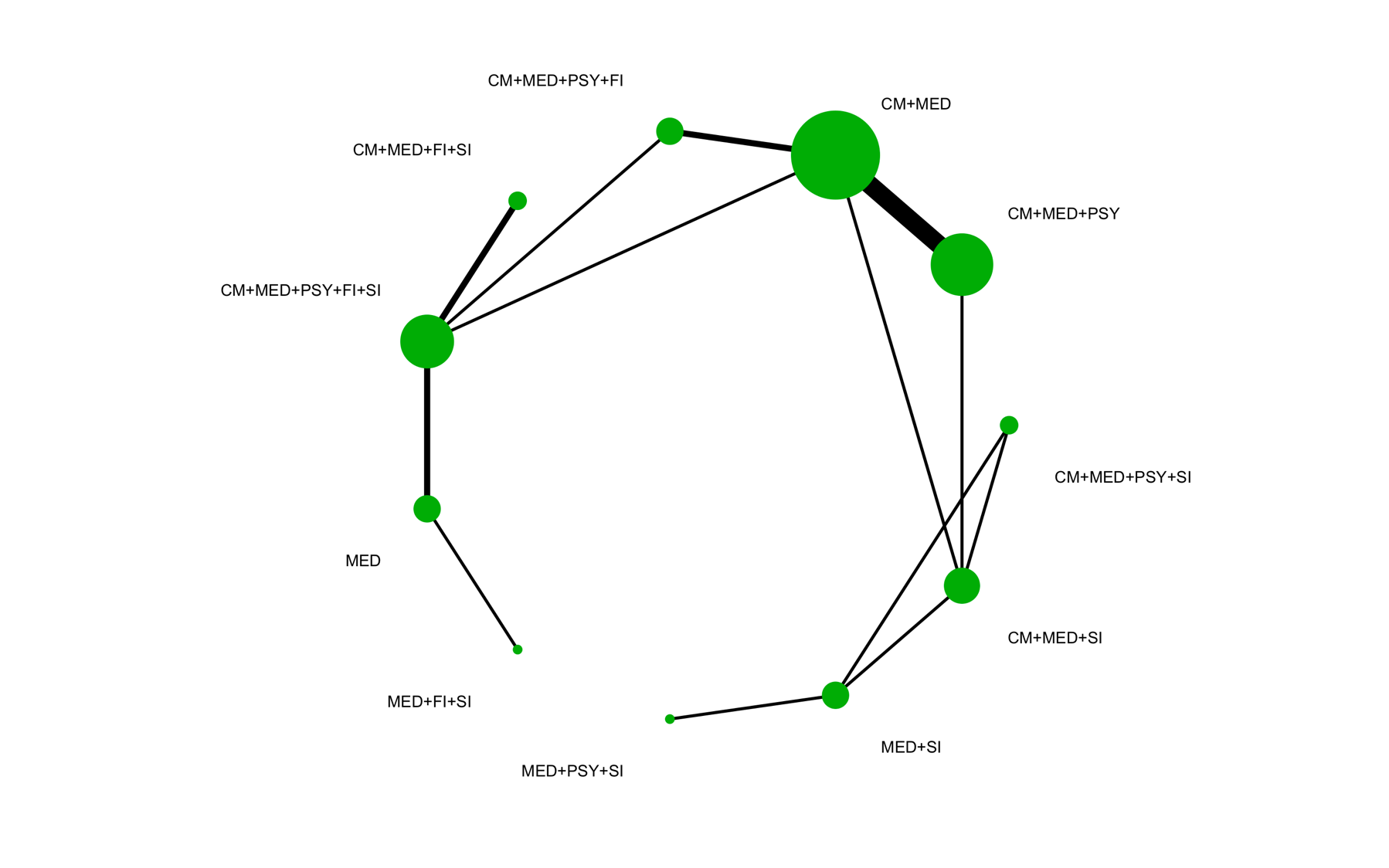


Nodes represent combinations of components, and lines denote trials performing the corresponding comparison. The width of the lines is proportional to the number of trials comparing each pair of treatments. The size of the nodes is proportional to the number of randomised participants.

# COMPONENT NETWORK META-ANALYSIS

|  | Positive symptoms (3 month follow-up) | Negative symptoms (3 month follow-up) | Positive symptoms (1 year follow-up) | Negative symptoms (1 year follow-up) | Dropouts (end of treatment) | Social functioning (1 year follow-up) | Depressive symptoms (1 year follow-up) |
| --- | --- | --- | --- | --- | --- | --- | --- |
| CASE MANAGEMENT | 0.25  (-0.45 to 0.94) p = 0.48 | -0.08  (-1.00 to 0.84) p = 0.87 | -1.05  (-2.02 to -0.08) p = 0.033 | -1.17  (-2.24 to -0.11) p = 0.030 | 0.92  (0.66 to 1.29) p = 0.63 | -0.24  (-1.47 to 0.99) p = 0.70 | 0.00  (-1.09 to 1.08) p = 0.99 |
| PSYCHOLOGICAL INTERVENTIONS | -0.07  (-0.29 to 0.14) p = 0.51 | -0.24  (-0.44 to -0.05) p = 0.014 | -0.37  (-0.90 to 0.15) p = 0.16 | -0.26  (-0.94 to 0.42) p = 0.45 | 1.08  (0.86 to 1.35) p = 0.53 | -0.52  (-1.05 to 0.01) p = 0.052 | 0.17  (-0.18 to 0.52) p = 0.33 |
| FAMILY INTERVENTIONS | -0.30  (-0.78 to 0.17) p = 0.21 | 0.57  (-0.47 to 1.62) p = 0.28 | -0.22  (-1.53 to 1.09) p = 0.74 | 0.52  (-1.61 to 2.65) p = 0.63 | 0.82  (0.57 to 1.20) p = 0.32 | -0.02  (-1.66 to 1.62) p = 0.98 | . |
| SOCIAL INTERVENTIONS | 0.18  (-0.29 to 0.64) p = 0.46 | -0.19  (-0.68 to 0.31) p = 0.46 | 0.50  (-0.75 to 1.75) p = 0.43 | 0.01  (-1.70 to 1.72) p = 0.99 | 0.88  (0.63 to 1.23) p = 0.45 | -0.17  (-1.32 to 0.98) p = 0.77 | . |

This table shows the component-specific incremental standardised mean differences (incremental risk ratios for Dropouts) of adding the row-defining component to an EIP treatment package (containing pharmacotherapy). Negative values represent beneficial effects (reductions in symptom scores). For dropouts, values less than 1 represent beneficial effects (reduced dropouts). Some cells are empty (.) because there were insufficient studies to enable a component analysis for the relevant comparison


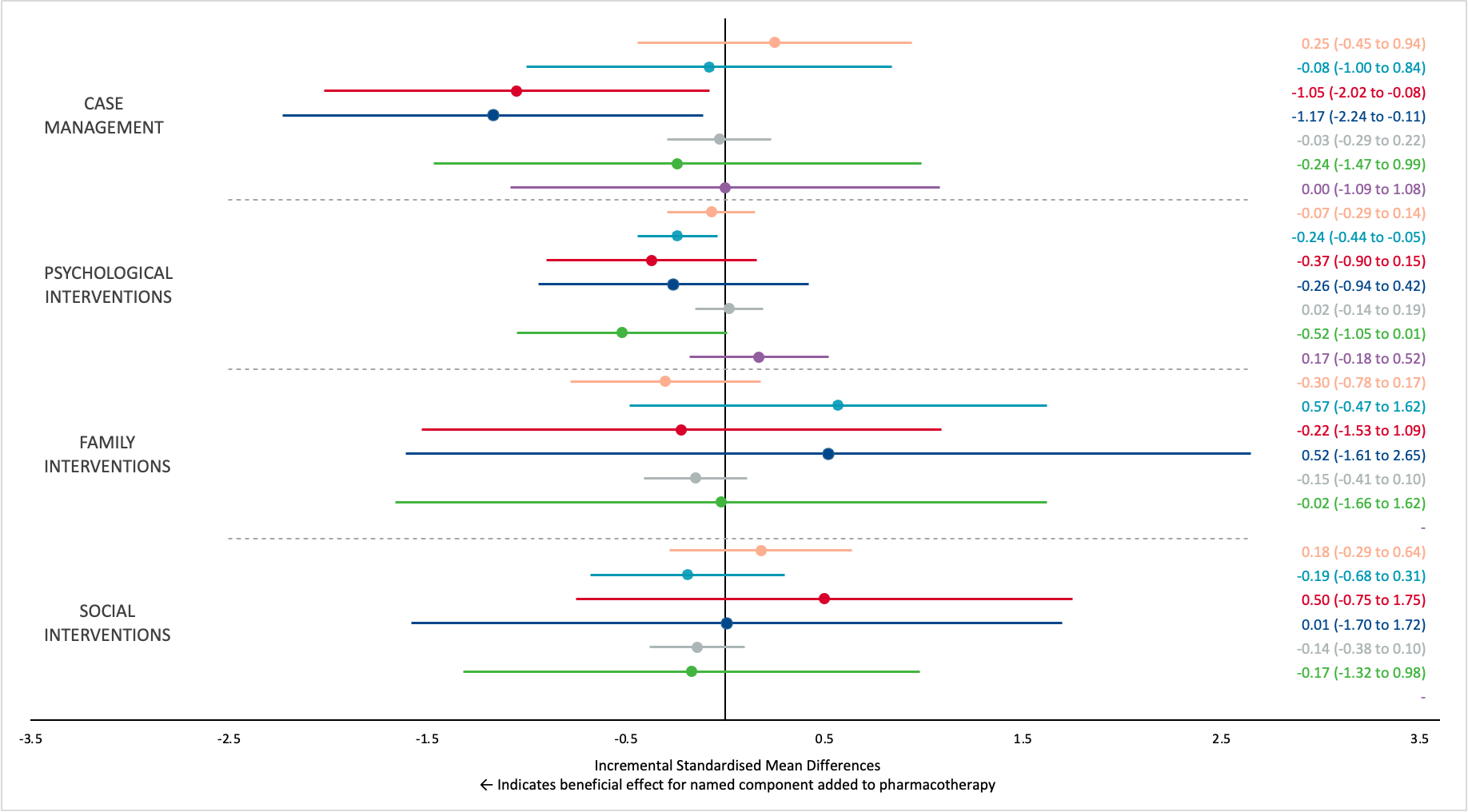


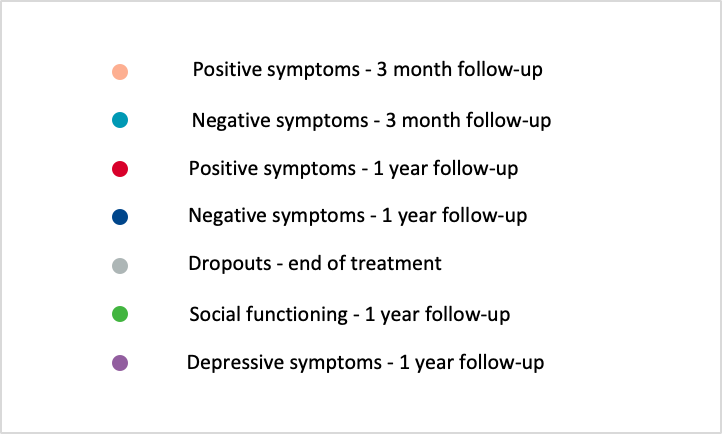


This plot shows the component-specific incremental standardised mean differences of adding the row-defining component to an EIP intervention package (including pharmacotherapy), for each outcome. For dropouts, incremental risk ratios were converted to incremental standardised mean differences for plotting purposes.^41^ It was not possible to examine the effect of family interventions or social interventions for the outcome ‘depressive symptoms’ due to insufficient studies to isolate these components.

# SENSITIVITY ANALYSIS

We conducted the following sensitivity analyses for our component network meta-analysis:

Sensitivity analysis 1: We examined the impact of studies which included participants aged 16-18 by excluding them from the analyses.

Sensitivity analysis 2: We examined the impact of studies where interventions were >3 years duration by excluding them from the analysis.

## **Sensitivity Analysis 1**

The following studies were excluded:

Alvarez-Jiminez 2021; De Haan 2022; Jackson HJ 2008; Jackson C 2009; Liu 2019; Myin-Germeys 2022; Robinson 2022; Uzenoff 2008; Valencia 2012.

The results of this sensitivity analysis are displayed in the tables below. None of the conclusions of our study were altered by this analysis. The magnitude of some of the effect sizes for associations identified in the primary analysis differed slightly. There was evidence that in this restricted sample, the addition of case management to an EIP package was also associated with improvements in social functioning at 1 year follow-up (iSMD, -0.82; 95% CI, -1.62 to -0.02; p = 0.044) while family interventions were actually associated with worsened social functioning at 1 year follow-up (iSMD, 1.59; 95% CI, 0.02 to 3.16; p = 0.047).

Results of adjusted component analysis:

|  | Positive symptoms (3 month follow-up) | Negative symptoms (3 month follow-up) | Positive symptoms (1 year follow-up) | Negative symptoms (1 year follow-up) | Dropouts (end of treatment) | Social functioning (1 year follow-up) | Depressive symptoms (1 year follow-up) |
| --- | --- | --- | --- | --- | --- | --- | --- |
| CASE MANAGEMENT | . | . | -1.69  (-2.70 to -0.68) p = 0.0010 | -1.92  (-3.47 to -0.37) p = 0.015 | 0.80  (0.38 to 1.65) p = 0.54 | -0.82  (-1.62 to -0.02) p = 0.044 | 0.00  (-1.09 to 1.08) p = 1.00 |
| PSYCHOLOGICAL INTERVENTIONS | -0.02  (-0.29 to 0.25) p = 0.87 | -0.20  (-0.44 to 0.05) p = 0.11 | -0.60  (-1.19 to -0.01) p = 0.045 | -0.30  (-1.31 to 0.72) p = 0.57 | 0.95  (0.67 to 1.35) p = 0.78 | -0.79  (-1.15 to -0.42) p < 0.001 | 0.17  (-0.18 to 0.52) p = 0.20 |
| FAMILY INTERVENTIONS | -0.31  (-0.85 to 0.23) p = 0.26 | 0.50  (-0.63 to 1.63) p = 0.39 | -0.29  (-1.59 to 1.01) p = 0.66 | -0.19  (-3.38 to 3.75) p = 0.92 | 0.96  (0.53 to 1.74) p = 0.90 | 1.59  (0.02 to 3.16) p = 0.047 | . |
| SOCIAL INTERVENTIONS | 0.19  (-0.33 to 0.71) p = 0.47 | -0.16  (-0.71 to 0.40) p = 0.58 | 0.33  (-0.88 to 1.53) p = 0.60 | -0.01  (-2.12 to 2.10) p = 0.99 | 0.79  (0.46 to 1.37) p = 0.40 | -0.24  (-0.90 to 0.42) p = 0.47 | . |

This table shows the component-specific incremental standardised mean differences (incremental risk ratios for Dropouts) of adding the row-defining component to an EIP treatment package (containing pharmacotherapy). Negative values represent beneficial effects (reductions in symptom scores). For dropouts, values less than 1 represent beneficial effects (reduced dropouts). Some cells are empty (.) because there were insufficient studies to enable a component analysis for the relevant comparison

## **Sensitivity Analysis 2**

The following studies were excluded:

De Haan 2022.

The results of this sensitivity analysis are displayed in the tables below. None of the conclusions of our study were altered by this analysis. The magnitude of some of the effect sizes for associations identified in the primary analysis differed slightly.

Results of adjusted component analysis:

|  | Positive symptoms (3 month follow-up) | Negative symptoms (3 month follow-up) | Positive symptoms (1 year follow-up) | Negative symptoms (1 year follow-up) | Dropouts (end of treatment) | Social functioning (1 year follow-up) | Depressive symptoms (1 year follow-up) |
| --- | --- | --- | --- | --- | --- | --- | --- |
| CASE MANAGEMENT | 0.25  (-0.45 to 0.94) p = 0.44 | -0.08  (-1.00 to 0.84) p = 0.43 | -1.15  (-2.24 to -0.07) p = 0.0052 | -1.33  (-2.54 to -0.12) p = 0.020 | 0.91  (0.57 to 1.45) p = 0.68 | -0.24  (-1.47 to 0.99) p = 0.77 | 0.00  (-1.09 to 1.08) p = 1.00 |
| PSYCHOLOGICAL INTERVENTIONS | -0.07  (-0.29 to 0.14) p = 0.51 | -0.24  (-0.44 to -0.05) p = 0.014 | -0.39  (-0.95 to 0.18) p = 0.093 | -0.27  (-1.01 to 0.47) p = 0.45 | 1.06  (0.84 to 1.35) p = 0.61 | -0.52  (-1.05 to 0.01) p = 0.074 | 0.17  (-0.18 to 0.52) p = 0.23 |
| FAMILY INTERVENTIONS | -0.30  (-0.78 to 0.17) p = 0.21 | 0.57  (-0.47 to 1.62) p = 0.28 | -0.27  (-1.67 to 1.13) p = 0.55 | 0.35  (-2.01 to 2.70) p = 0.82 | 0.83  (0.56 to 1.22) p = 0.34 | -0.02  (-1.66 to 1.62) p = 0.58 | . |
| SOCIAL INTERVENTIONS | 0.18  (-0.29 to 0.64) p = 0.46 | -0.19  (-0.68 to 0.31) p = 0.46 | 0.45  (-0.89 to 1.78) p = 0.52 | 0.00  (-1.86 to 1.87) p = 1.00 | 0.88  (0.62 to 1.25) p = 0.47 | -0.17  (-1.32 to 0.98) p = 0.68 | . |

This table shows the component-specific incremental standardised mean differences (incremental risk ratios for Dropouts) of adding the row-defining component to an EIP treatment package (containing pharmacotherapy). Negative values represent beneficial effects (reductions in symptom scores). For dropouts, values less than 1 represent beneficial effects (reduced dropouts). Some cells are empty (.) because there were insufficient studies to enable a component analysis for the relevant comparison.

# CINEMA : EVALUATION OF CERTAINTY OF EVIDENCE

We evaluated the certainty of evidence using the Confidence in Network Meta-Analysis Software.^42^ CINeMA is a software which uses the *netmeta* R-package. We assessed each network estimate according to the following criteria:

1. Risk of bias: We assigned each trial an overall risk of bias judgement according to the following criteria: a study with a judgements of high risk of bias in one or more domains has been considered a ‘high’ risk of bias study, a study with judgements of ‘some concerns’ in two or more domains has been considered to have ‘some concerns’, and a study with only one domain with ‘some concerns’ has been considered a ‘low’ risk of bias study. We rated each pairwise comparison based on the average bias judgment across direct estimate contribution from individual studies to the contribution matrix. This assessment was conducted for two of our primary outcomes (negative symptoms at 3 months and 1 year follow-up) by using the Confidence in Network Meta-Analysis Software.^42^ We selected these outcomes based on evidence that negative symptoms may be particularly important for predicting longer-term prognosis.^43, 44^
2. Indirectness: We trust that the included studies in our review answered the targeted research question. However, we completed an additional judgement of the study indirectness (defined as the ‘relevance of the included studies to the research question... whether populations, interventions, outcomes, and study settings are representative of the settings, populations, or outcomes about which reviewers want to make inferences’) by applying the following criteria. We considered any study which violated two of these criteria to have ‘moderate’ indirectness, and any study that violated three or more to have ‘high’ indirectness.
   1. All people with any psychotic disorder within 5 years of onset are eligible (violated if inclusion criteria are more restrictive than this e.g. disorder within 1 year of onset/ specific diagnosis such as schizophrenia only, or other criteria such as minimum scores on symptom scales etc).
   2. All participants received some combination of the components pharmacotherapy, case management, psychological therapy, family intervention, social intervention (violated if study only stated what was ‘available/ offered’ in intervention/ control arms and not what participants actually received).
   3. Interventions were conducted in a ‘generic’ community mental health setting (violated if conducted in an inpatient setting or other setting with particular conditions for access e.g. prison).

Risk of Bias Chart for outcome 1 : Negative symptoms at 3-month follow-up.

| **Study** | **Randomisation Process** | **Deviations from intended interventions** | **Missing outcome data** | **Measurement of the outcome** | **Selection of the reported result** | **Overall Risk of bias** |
| --- | --- | --- | --- | --- | --- | --- |
| Jackson 2008 | Low | Low | High | Low | Some concerns | High |
| Kuipers 2004 | Low | Low | Some concerns | Some concerns | Low | Some concerns |
| Lecomte 2008 | Some concerns | Low | Some concerns | Some concerns | Some concerns | Some concerns |
| MacDougall 2019 | Low | Low | Some concerns | Low | Low | Low |
| Penn 2011 | Low | Low | Some concerns | Low | Low | Low |
| Pos 2019 | Some concerns | Low | Some concerns | Low | Some concerns | Some concerns |
| Robinson 2022 | Some concerns | Low | Some concerns | Low | Low | Some concerns |
| Uzenoff 2008 | Some concerns | Low | Some concerns | Low | Some concerns | Some concerns |
| Vidarsdottir 2019 | Low | Low | Some concerns | Some concerns | Some concerns | Some concerns |

Risk of Bias Chart showing the contribution of low, moderate or high RoB comparisons to each network estimate for outcome 1 : Negative symptoms at 3-month follow-up.

| MED:CM+MED+PSY+FI+SI |
| --- |
| CM+MED:CM+MED+PSY |
| CM+MED:CM+MED+SI |
| CM+MED:CM+MED+PSY+FI+SI |
| CM+MED+PSY:CM+MED+SI |
| CM+MED+FI+SI:CM+MED+PSY+FI+SI |
| MED: CM+MED |
| MED: CM+MED+PSY |
| MED:CM+MED+SI |
| MED:CM+MED+FI+SI |
| CM+MED:CM+MED+FI+SI |
| CM+MED+PSY:CM+MED+FI+SI |
| CM+MED+PSY:CM+MED+PSY+FI+SI |
| CM+MED+SI:CM+MED+FI+SI |
| CM+MED+SI:CM+MED+PSY+FI+S |


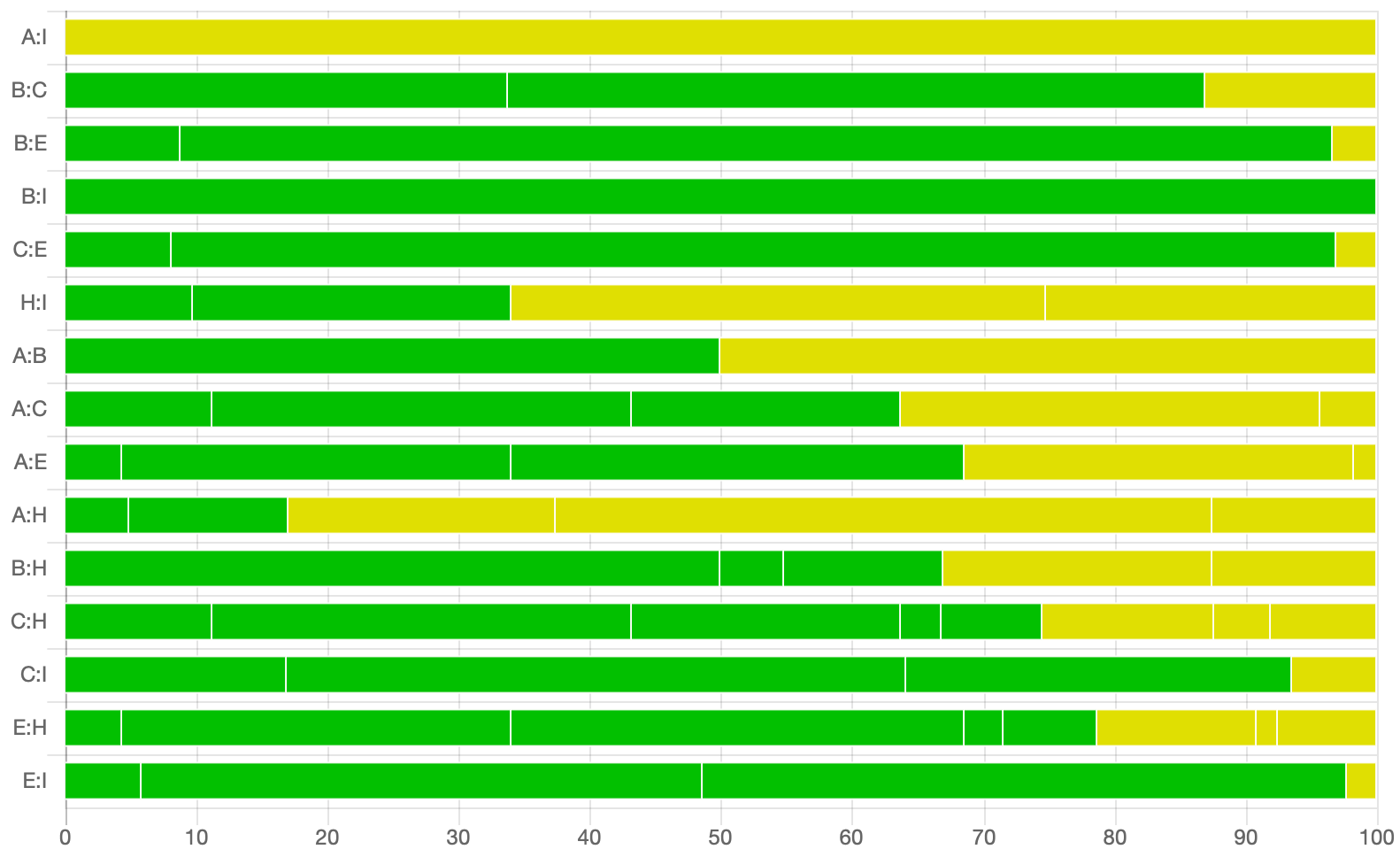


Evaluation of the Certainty of evidence Using CINEMA Framework, mixed evidence for outcome 1 : Negative symptoms at 3-month follow-up.

| **Comparison** | **Number of studies** | **Within-study bias** | **Reporting bias** | **Indirectness** | **Imprecision** | **Heterogeneity** | **Incoherence** | **Confidence rating** |
| --- | --- | --- | --- | --- | --- | --- | --- | --- |
| MED:CM+MED+PSY+FI+SI | 1 | Some concerns | Some concerns | Some concerns | Major concerns | No concerns | No concerns | Very low |
| CM+MED:CM+MED+PSY | 3 | Some concerns | Some concerns | No concerns | Some concerns | Some concerns | No concerns | Very low |
| CM+MED:CM+MED+SI | 1 | Some concerns | Some concerns | No concerns | Major concerns | No concerns | No concerns | Very low |
| CM+MED:CM+MED+PSY+FI+SI | 1 | Some concerns | Some concerns | No concerns | Major concerns | No concerns | No concerns | Very low |
| CM+MED+PSY:CM+MED+SI | 1 | Some concerns | Some concerns | No concerns | Major concerns | No concerns | No concerns | Very low |
| CM+MED+FI+SI:CM+MED+PSY+FI+SI | 4 | Some concerns | Some concerns | Some concerns | Some concerns | Some concerns | No concerns | Very low |
| MED: CM+MED | 0 | Some concerns | Some concerns | Some concerns | Major concerns | No concerns | No concerns | Very low |
| MED: CM+MED+PSY | 0 | Some concerns | Some concerns | No concerns | Major concerns | No concerns | No concerns | Very low |
| MED:CM+MED+SI | 0 | Some concerns | Some concerns | No concerns | Major concerns | No concerns | No concerns | Very low |
| MED:CM+MED+FI+SI | 0 | Some concerns | Some concerns | Some concerns | Some concerns | Some concerns | No concerns | Very low |
| CM+MED:CM+MED+FI+SI | 0 | Some concerns | Some concerns | No concerns | Major concerns | No concerns | No concerns | Very low |
| CM+MED+PSY:CM+MED+FI+SI | 0 | Some concerns | Some concerns | No concerns | Major concerns | No concerns | No concerns | Very low |
| CM+MED+PSY:CM+MED+PSY+FI+SI | 0 | Some concerns | Some concerns | No concerns | Major concerns | No concerns | No concerns | Very low |
| CM+MED+SI:CM+MED+FI+SI | 0 | Some concerns | Some concerns | No concerns | Major concerns | No concerns | No concerns | Very low |
| CM+MED+SI:CM+MED+PSY+FI+S | 0 | Some concerns | Some concerns | No concerns | Major concerns | No concerns | No concerns | Very low |

Risk of Bias Chart for outcome 2 : Negative symptoms at 1-year follow-up.

| **Study** | **Randomisation Process** | **Deviations from intended interventions** | **Missing outcome data** | **Measurement of the outcome** | **Selection of the reported result** | **Overall Risk of bias** |
| --- | --- | --- | --- | --- | --- | --- |
| Chien 2019 | Low | Low | Some concerns | Low | Low | Low |
| Chien 2019 | Low | Low | Some concerns | Some concerns | Some concerns | Some concerns |
| Fan 2005 | Some concerns | Low | Some concerns | Some concerns | Some concerns | Some concerns |
| Jackson 2008 | Low | Low | High | Low | Some concerns | High |
| Kuipers 2004 | Low | Low | Some concerns | Some concerns | Low | Some concerns |
| Lecomte 2008 | Some concerns | Low | Some concerns | Some concerns | Some concerns | Some concerns |
| Myin-Germeys 2022 | Some concerns | Low | Low | Low | Low | Low |
| Penn 2011 | Low | Low | Some concerns | Low | Low | Low |
| Robinson 2022 | Some concerns | Low | Some concerns | Low | Low | Some concerns |
| Sonmez 2020 | Low | Low | Low | Low | Some concerns | Low |
| Srihari 2015 | Low | Low | Some concerns | Some concerns | Low | Some concerns |
| Valencia 2012 | Some concerns | Low | Some concerns | Low | Some concerns | Some concerns |

Risk of Bias Chart showing the contribution of low, moderate or high RoB comparisons to each network estimate for outcome 2 : Negative symptoms at 1-year follow-up.


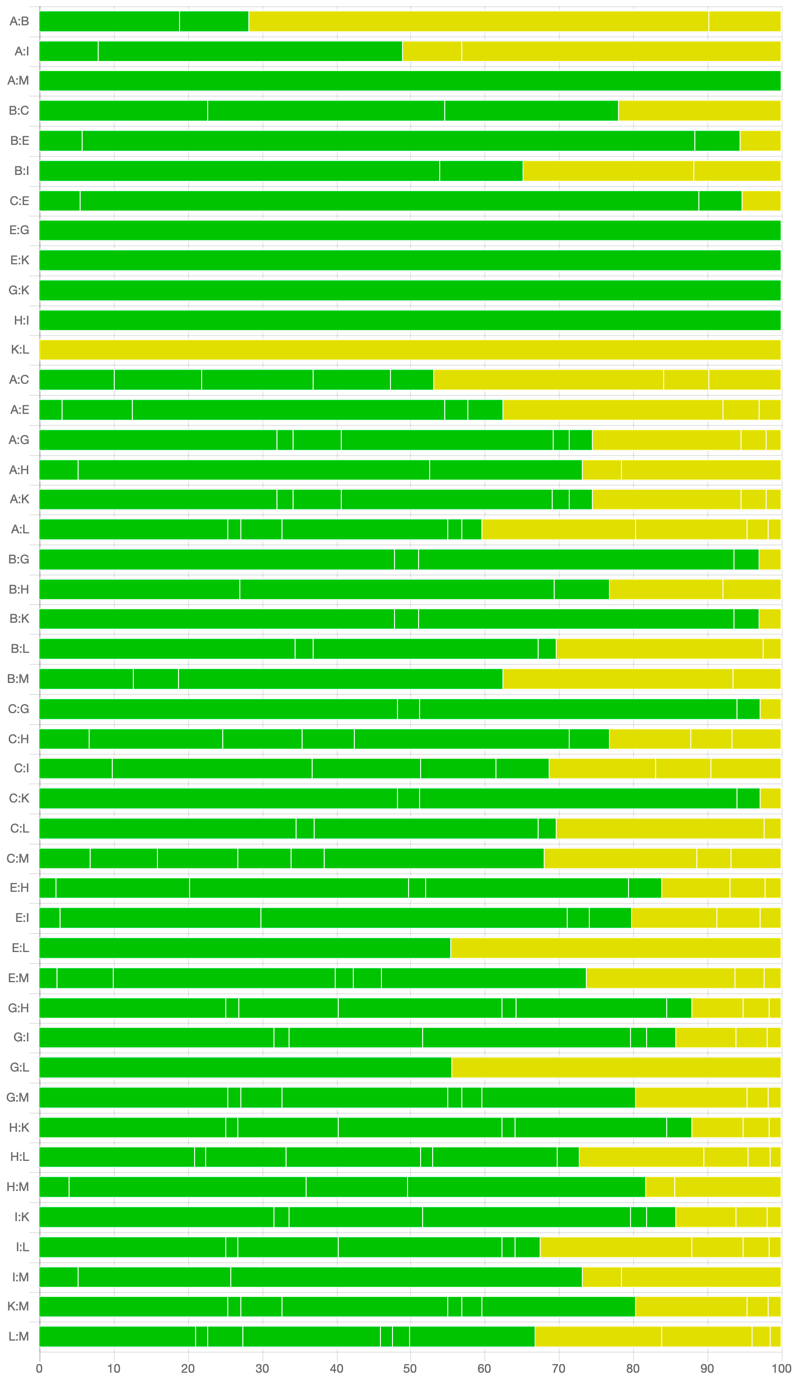

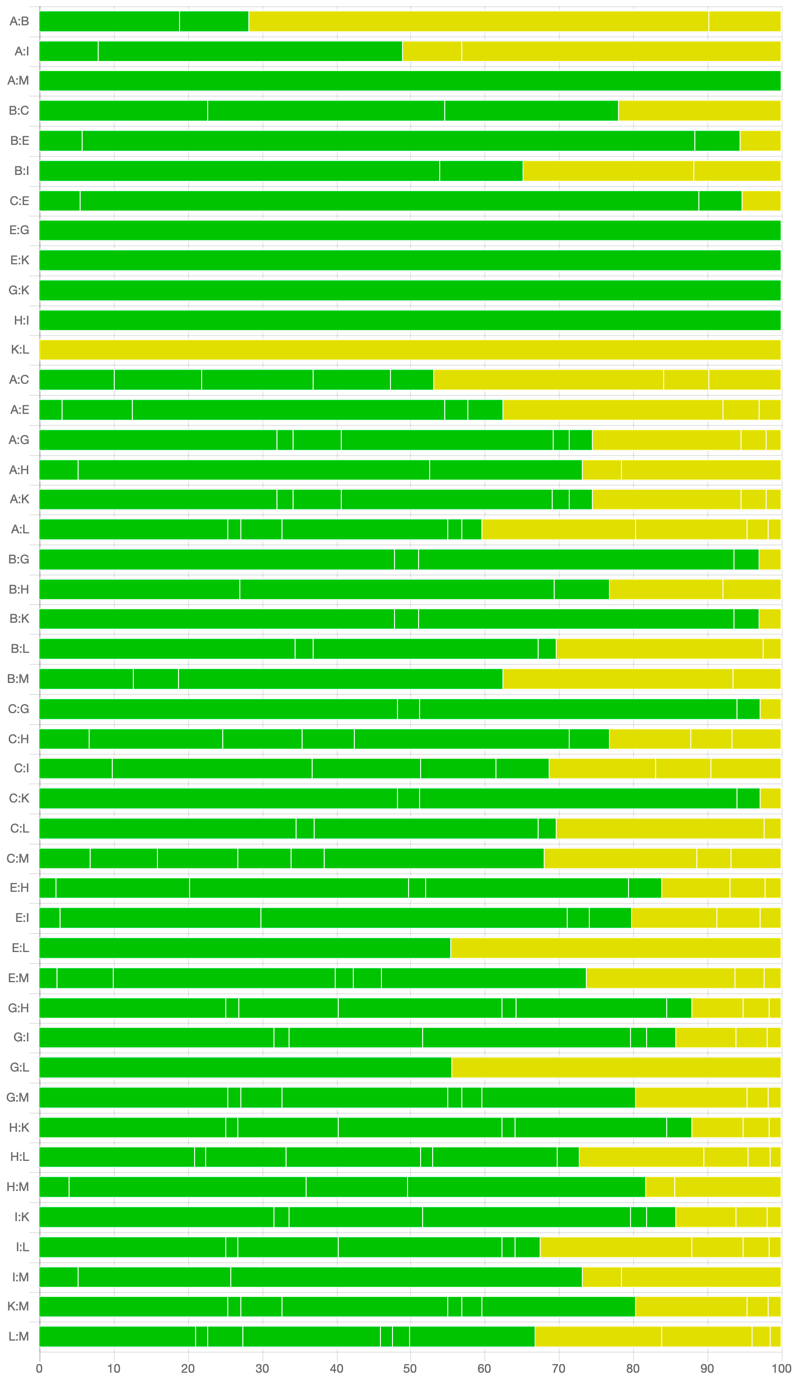


| MED:CM+MED |
| --- |
| MED:CM+MED+PSY+FI+SI |
| MED:MED+FI+SI |
| CM+MED:CM+MED+PSY |
| CM+MED:CM+MED+SI |
| CM+MED:CM+MED+PSY+FI+SI |
| CM+MED+PSY:CM+MED+SI |
| CM+MED+SI:CM+MED+PSY+SI |
| CM+MED+SI:MED+SI |
| CM+MED+PSY+SI:MED+SI |
| CM+MED+FI+SI:CM+MED+PSY+FI+SI |
| MED+SI:MED+PSY+SI |
| MED:CM+MED+PSY |
| MED:CM+MED+SI |
| MED:CM+MED+PSY+SI |
| MED:CM+MED+FI+SI |
| MED:MED+SI |
| MED:MED+PSY+SI |
| CM+MED:CM+MED+PSY+SI |


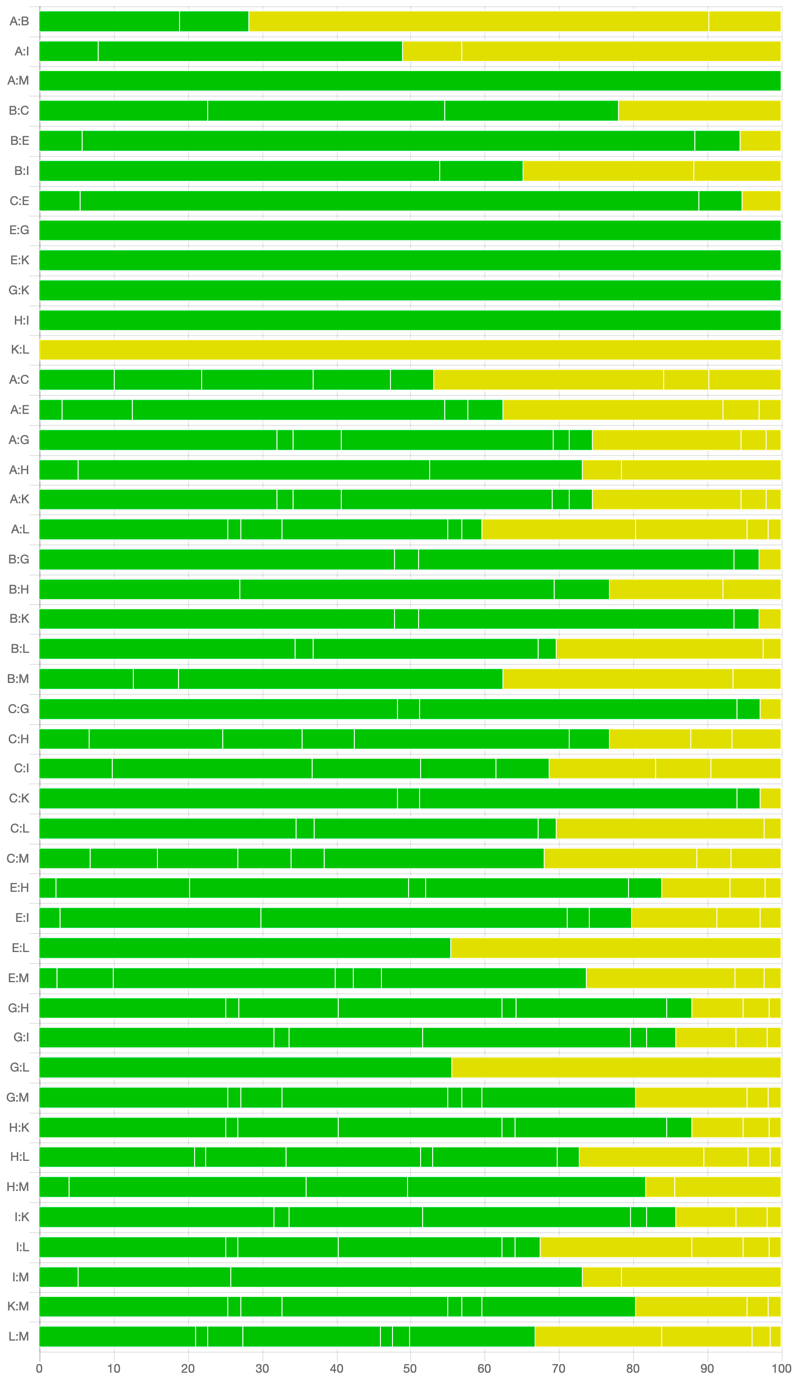

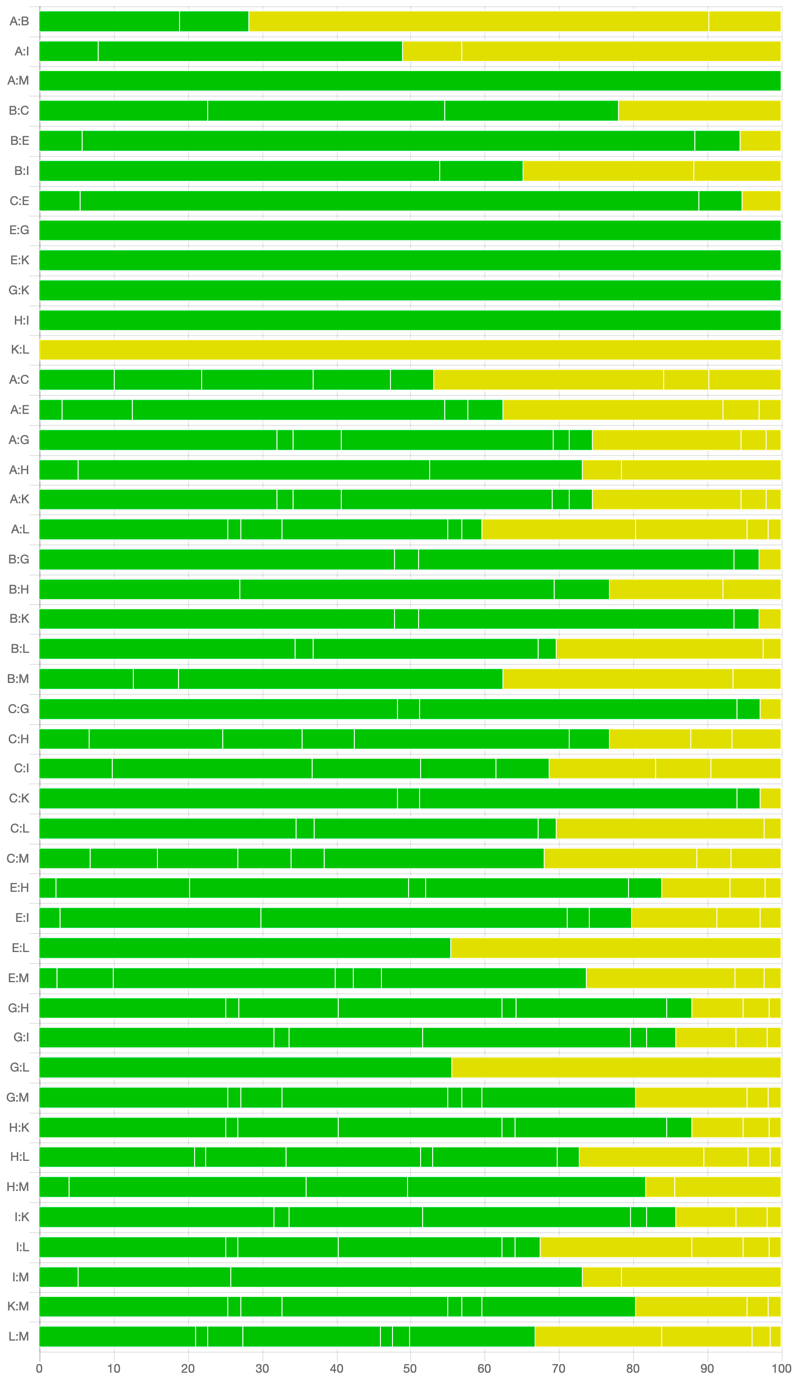


| CM+MED:CM+MED+FI+SI |
| --- |
| CM+MED:MED+SI |
| CM+MED:MED+PSY+SI |
| CM+MED:MED+FI+SI |
| CM+MED+PSY:CM+MED+PSY+SI |
| CM+MED+PSY:CM+MED+FI+SI |
| CM+MED+PSY:CM+MED+PSY+FI+SI |
| CM+MED+PSY:MED+SI |
| CM+MED+PSY:MED+PSY+SI |
| CM+MED+PSY:MED+FI+SI |
| CM+MED+SI:CM+MED+FI+SI |
| CM+MED+SI:CM+MED+PSY+FI+SI |
| CM+MED+SI:MED+PSY+SI |
| CM+MED+SI:MED+FI+SI |
| CM+MED+PSY+SI:CM+MED+FI+SI |
| CM+MED+PSY+SI:CM+MED+PSY+FI+SI |
| CM+MED+PSY+SI:MED+PSY+SI |
| CM+MED+PSY+SI:MED+FI+SI |
| CM+MED+FI+SI:MED+SI |
| CM+MED+FI+SI:MED+PSY+SI |


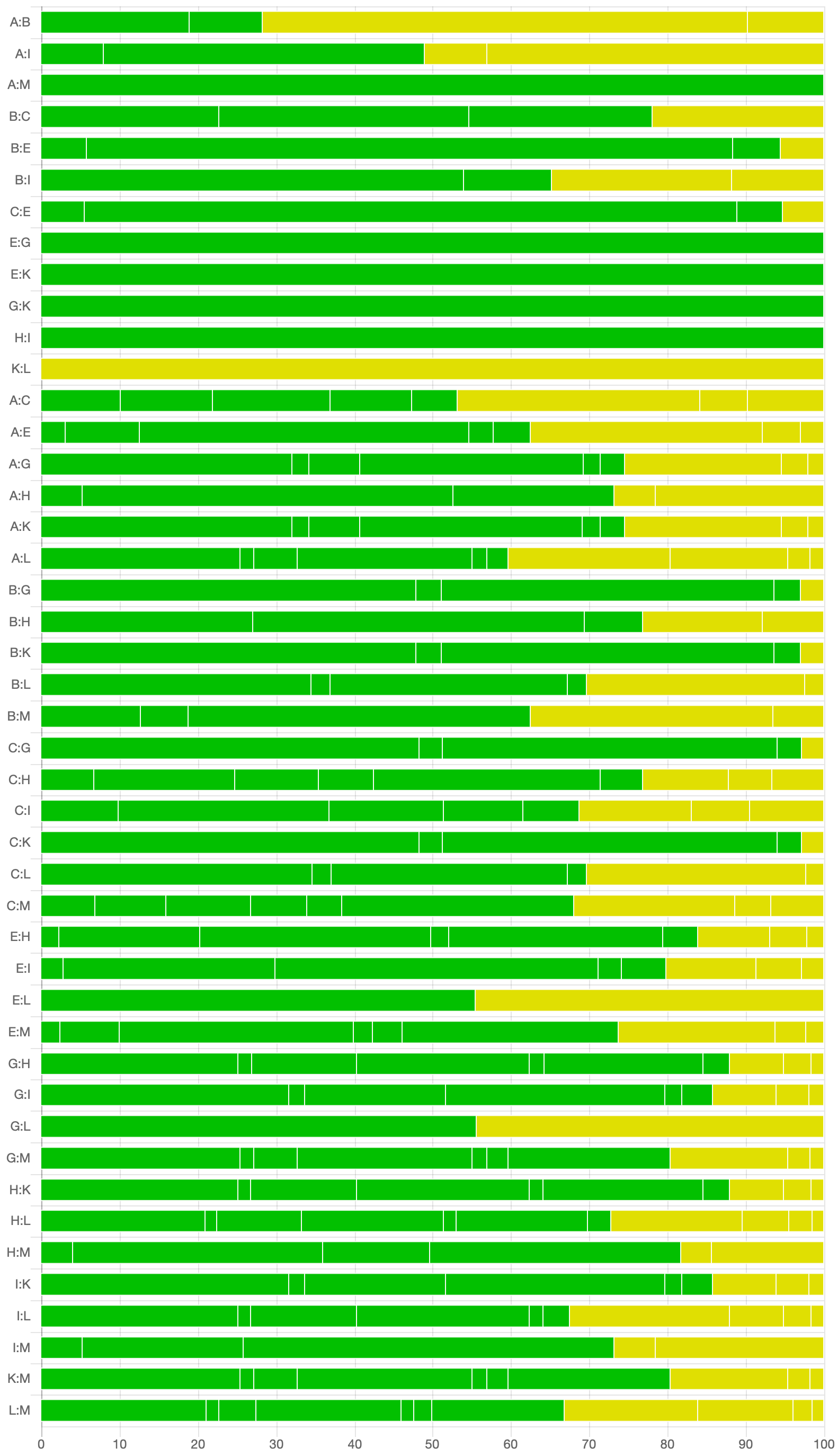


| CM+MED+FI+SI:MED+FI+SI |
| --- |
| CM+MED+PSY+FI+SI:MED+SI |
| CM+MED+PSY+FI+SI:MED+PSY+SI |
| CM+MED+PSY+FI+SI:MED+FI+SI |
| MED+SI:MED+FI+SI |
| MED+PSY+SI:MED+FI+SI |

Risk of Bias Chart showing the contribution of low, moderate or high RoB comparisons to each network estimate for outcome 2 : Negative symptoms at 1-year follow-up.

| **Comparison** | **Number of studies** | **Within-study bias** | **Reporting bias** | **Indirectness** | **Imprecision** | **Heterogeneity** | **Incoherence** | **Confidence rating** |
| --- | --- | --- | --- | --- | --- | --- | --- | --- |
| MED:CM+MED | 1 | Some concerns | Some concerns | Some concerns | No concerns | Some concerns | Major concerns | Very low |
| MED:CM+MED+PSY+FI+SI | 2 | Some concerns | Some concerns | Some concerns | Major concerns | No concerns | Major concerns | Very low |
| MED:MED+FI+SI | 1 | Some concerns | Some concerns | No concerns | Major concerns | No concerns | Major concerns | Very low |
| CM+MED:CM+MED+PSY | 4 | Some concerns | Some concerns | No concerns | Major concerns | No concerns | Major concerns | Very low |
| CM+MED:CM+MED+SI | 1 | Some concerns | Some concerns | No concerns | Major concerns | No concerns | No concerns | Very low |
| CM+MED:CM+MED+PSY+FI+SI | 1 | Some concerns | Some concerns | No concerns | No concerns | Major concerns | Major concerns | Very low |
| CM+MED+PSY:CM+MED+SI | 1 | Some concerns | Some concerns | No concerns | Major concerns | No concerns | No concerns | Very low |
| CM+MED+SI:CM+MED+PSY+SI | 1 | No concerns | Some concerns | No concerns | Major concerns | No concerns | Major concerns | Very low |
| CM+MED+SI:MED+SI | 1 | No concerns | Some concerns | No concerns | Major concerns | No concerns | Major concerns | Very low |
| CM+MED+PSY+SI:MED+SI | 1 | No concerns | Some concerns | No concerns | Major concerns | No concerns | Major concerns | Very low |
| CM+MED+FI+SI:CM+MED+PSY+FI+SI | 1 | No concerns | Some concerns | No concerns | Major concerns | No concerns | Major concerns | Very low |
| MED+SI:MED+PSY+SI | 1 | Some concerns | Some concerns | Some concerns | Major concerns | No concerns | Major concerns | Very low |
| MED:CM+MED+PSY | 0 | Some concerns | Some concerns | No concerns | No concerns | No concerns | Major concerns | Very low |
| MED:CM+MED+SI | 0 | Some concerns | Some concerns | No concerns | No concerns | Major concerns | Major concerns | Very low |
| MED:CM+MED+PSY+SI | 0 | Some concerns | Some concerns | No concerns | No concerns | Major concerns | Major concerns | Very low |
| MED:CM+MED+FI+SI | 0 | Some concerns | Some concerns | No concerns | Major concerns | No concerns | Major concerns | Very low |
| MED:MED+SI | 0 | Some concerns | Some concerns | No concerns | No concerns | Major concerns | Major concerns | Very low |
| MED:MED+PSY+SI | 0 | Some concerns | Some concerns | No concerns | No concerns | Major concerns | Major concerns | Very low |
| CM+MED:CM+MED+PSY+SI | 0 | No concerns | Some concerns | No concerns | Major concerns | No concerns | Major concerns | Very low |
| CM+MED:CM+MED+FI+SI | 0 | Some concerns | Some concerns | No concerns | No concerns | Major concerns | Major concerns | Very low |
| CM+MED:MED+SI | 0 | No concerns | Some concerns | No concerns | Major concerns | No concerns | Major concerns | Very low |
| CM+MED:MED+PSY+SI | 0 | Some concerns | Some concerns | No concerns | Major concerns | No concerns | Major concerns | Very low |
| CM+MED:MED+FI+SI | 0 | Some concerns | Some concerns | No concerns | Major concerns | No concerns | Major concerns | Very low |
| CM+MED+PSY:CM+MED+PSY+SI | 0 | No concerns | Some concerns | No concerns | Major concerns | No concerns | Major concerns | Very low |
| CM+MED+PSY:CM+MED+FI+SI | 0 | Some concerns | Some concerns | No concerns | No concerns | Major concerns | Major concerns | Very low |
| CM+MED+PSY:CM+MED+PSY+FI+SI | 0 | Some concerns | Some concerns | No concerns | No concerns | Major concerns | Major concerns | Very low |
| CM+MED+PSY:MED+SI | 0 | No concerns | Some concerns | No concerns | Major concerns | No concerns | Major concerns | Very low |
| CM+MED+PSY:MED+PSY+SI | 0 | Some concerns | Some concerns | No concerns | Major concerns | No concerns | Major concerns | Very low |
| CM+MED+PSY:MED+FI+SI | 0 | Some concerns | Some concerns | No concerns | No concerns | Major concerns | Major concerns | Very low |
| CM+MED+SI:CM+MED+FI+SI | 0 | Some concerns | Some concerns | No concerns | Major concerns | No concerns | Major concerns | Very low |
| CM+MED+SI:CM+MED+PSY+FI+SI | 0 | Some concerns | Some concerns | No concerns | Some concerns | Some concerns | Major concerns | Very low |
| CM+MED+SI:MED+PSY+SI | 0 | No concerns | Some concerns | No concerns | Major concerns | No concerns | Major concerns | Very low |
| CM+MED+SI:MED+FI+SI | 0 | Some concerns | Some concerns | No concerns | Major concerns | No concerns | Major concerns | Very low |
| CM+MED+PSY+SI:CM+MED+FI+SI | 0 | Some concerns | Some concerns | No concerns | Major concerns | No concerns | Major concerns | Very low |
| CM+MED+PSY+SI:CM+MED+PSY+FI+SI | 0 | Some concerns | Some concerns | No concerns | Some concerns | Some concerns | Major concerns | Very low |
| CM+MED+PSY+SI:MED+PSY+SI | 0 | No concerns | Some concerns | No concerns | Major concerns | No concerns | Major concerns | Very low |
| CM+MED+PSY+SI:MED+FI+SI | 0 | Some concerns | Some concerns | No concerns | Major concerns | No concerns | Major concerns | Very low |
| CM+MED+FI+SI:MED+SI | 0 | Some concerns | Some concerns | No concerns | Major concerns | No concerns | Major concerns | Very low |
| CM+MED+FI+SI:MED+PSY+SI | 0 | Some concerns | Some concerns | No concerns | Major concerns | No concerns | Major concerns | Very low |
| CM+MED+FI+SI:MED+FI+SI | 0 | Some concerns | Some concerns | No concerns | Major concerns | No concerns | Major concerns | Very low |
| CM+MED+PSY+FI+SI:MED+SI | 0 | Some concerns | Some concerns | No concerns | Major concerns | No concerns | Major concerns | Very low |
| CM+MED+PSY+FI+SI:MED+PSY+SI | 0 | Some concerns | Some concerns | No concerns | Major concerns | No concerns | Major concerns | Very low |
| CM+MED+PSY+FI+SI:MED+FI+SI | 0 | Some concerns | Some concerns | No concerns | Major concerns | No concerns | Major concerns | Very low |
| MED+SI:MED+FI+SI | 0 | Some concerns | Some concerns | No concerns | Major concerns | No concerns | Major concerns | Very low |
| MED+PSY+SI:MED+FI+SI | 0 | Some concerns | Some concerns | No concerns | Major concerns | No concerns | Major concerns | Very low |

# REFERENCES

1. Almerie MQ, Okba Al Marhi M, Jawoosh M, Alsabbagh M, Matar HE, Maayan N, et al. Social skills programmes for schizophrenia. *Cochrane Database Syst Rev* 2015; **2015**(6): CD009006.

2. National Institute for Health and Care Excellence. Access and waiting time standards for early intervention in psychosis. . *NICE* 2016.

3. Chang WC, Chan GH, Jim OT, Lau ES, Hui CL, Chan SK, et al. Optimal duration of an early intervention programme for first-episode psychosis: randomised controlled trial. *Br J Psychiatry* 2015; **206**(6): 492-500.

4. Alvarez-Jimenez M, Koval P, Schmaal L, Bendall S, O'Sullivan S, Cagliarini D, et al. The Horyzons project: a randomized controlled trial of a novel online social therapy to maintain treatment effects from specialist first-episode psychosis services. *World Psychiatry* 2021; **20**(2): 233-43.

5. Bertelsen M, Jeppesen P, Petersen L, Thorup A, Ohlenschlaeger J, le Quach P, et al. Five-year follow-up of a randomized multicenter trial of intensive early intervention vs standard treatment for patients with a first episode of psychotic illness: the OPUS trial. *Arch Gen Psychiatry* 2008; **65**(7): 762-71.

6. Cechnicki A, Bielanska A. The influence of early psychosocial intervention on the long-term clinical outcomes of people suffering from schizophrenia. *Psychiatr Pol* 2017; **51**(1): 45-61.

7. Chan SW, Yip B, Tso S, Cheng BS, Tam W. Evaluation of a psychoeducation program for Chinese clients with schizophrenia and their family caregivers. *Patient Educ Couns* 2009; **75**(1): 67-76.

8. Chien WT, Cheng HY, McMaster TW, Yip ALK, Wong JCL. Effectiveness of a mindfulness-based psychoeducation group programme for early-stage schizophrenia: An 18-month randomised controlled trial. *Schizophr Res* 2019; **212**: 140-9.

9. Chien WT, Cheung EFC, Mui JHC, Gray R, Ip G. Adherence therapy for schizophrenia: a randomised controlled trial. *Hong Kong Med J* 2019; **25 Suppl 2**(1): 4-9.

10. Gafoor R, Nitsch D, McCrone P, Craig TK, Garety PA, Power P, et al. Effect of early intervention on 5-year outcome in non-affective psychosis. *Br J Psychiatry* 2010; **196**(5): 372-6.

11. de Haan L, Linszen D, Wouters L, Zwinderman K, Dingemans P. Sustained specialized and family treatment in first-episode schizophrenia or related disorders: a 5-year randomized controlled trial. *Psychol Med* 2022; **53**(10): 1-8.

12. Drake RJ, Day CJ, Picucci R, Warburton J, Larkin W, Husain N, et al. A naturalistic, randomized, controlled trial combining cognitive remediation with cognitive-behavioural therapy after first-episode non-affective psychosis. *Psychol Med* 2014; **44**(9): 1889-99.

13. Erickson DH, Roes MM, DiGiacomo A, Burns A. "Individual Placement and Support" boosts employment for early psychosis clients, even when baseline rates are high. *Early Interv Psychiatry* 2021; **15**(3): 662-8.

14. Fan X. The effects of community nursing intervention on the recovery of patients with primary schizophrenia. . *Journal of the Linyi Medical College* 2005; **27**(6): 419-21.

15. Gonzalez-Ortega I, Vega P, Echeburua E, Alberich S, Fernandez-Sevillano J, Barbeito S, et al. A Multicentre, Randomised, Controlled Trial of a Combined Clinical Treatment for First-Episode Psychosis. *Int J Environ Res Public Health* 2021; **18**(14).

16. Grawe RW, Falloon IR, Widen JH, Skogvoll E. Two years of continued early treatment for recent-onset schizophrenia: a randomised controlled study. *Acta Psychiatr Scand* 2006; **114**(5): 328-36.

17. Haddock G, Tarrier N, Morrison AP, Hopkins R, Drake R, Lewis S. A pilot study evaluating the effectiveness of individual inpatient cognitive-behavioural therapy in early psychosis. *Soc Psychiatry Psychiatr Epidemiol* 1999; **34**(5): 254-8.

18. Hui CLM, Wong AKH, Ho ECN, Lam BST, Hui PWM, Tao TJ, et al. Effectiveness and optimal duration of early intervention treatment in adult-onset psychosis: a randomized clinical trial. *Psychol Med* 2023; **53**(6): 2339-51.

19. Jackson HJ, McGorry PD, Killackey E, Bendall S, Allott K, Dudgeon P, et al. Acute-phase and 1-year follow-up results of a randomized controlled trial of CBT versus Befriending for first-episode psychosis: the ACE project. *Psychol Med* 2008; **38**(5): 725-35.

20. Jackson C, Trower P, Reid I, Smith J, Hall M, Townend M, et al. Improving psychological adjustment following a first episode of psychosis: a randomised controlled trial of cognitive therapy to reduce post psychotic trauma symptoms. *Behav Res Ther* 2009; **47**(6): 454-62.

21. Kuipers E, Holloway F, Rabe-Hesketh S, Tennakoon L, Croydon O, Assertive Support T. An RCT of early intervention in psychosis: Croydon Outreach and Assertive Support Team (COAST). *Soc Psychiatry Psychiatr Epidemiol* 2004; **39**(5): 358-63.

22. Lecomte T, Leclerc C, Corbiere M, Wykes T, Wallace CJ, Spidel A. Group cognitive behavior therapy or social skills training for individuals with a recent onset of psychosis? Results of a randomized controlled trial. *J Nerv Ment Dis* 2008; **196**(12): 866-75.

23. Linke M, Jankowski KS, Wichniak A, Jarema M, Wykes T. Effects of cognitive remediation therapy versus other interventions on cognitive functioning in schizophrenia inpatients. *Neuropsychol Rehabil* 2019; **29**(3): 477-88.

24. Liu Y, Yang X, Gillespie A, Guo Z, Ma Y, Chen R, et al. Targeting relapse prevention and positive symptom in first-episode schizophrenia using brief cognitive behavioral therapy: A pilot randomized controlled study. *Psychiatry Res* 2019; **272**: 275-83.

25. MacDougall AG, Price E, Vandermeer MRJ, Lloyd C, Bird R, Sethi R, et al. Youth-focused group mindfulness-based intervention in individuals with early psychosis: A randomized pilot feasibility study. *Early Interv Psychiatry* 2019; **13**(4): 993-8.

26. Marchira CR, Supriyanto I, Subandi S, Good MJD, Good BJ. Brief interactive psychoeducation for caregivers of patients with early phase psychosis in Yogyakarta, Indonesia. *Early Interv Psychiatry* 2019; **13**(3): 469-76.

27. Myin-Germeys I, van Aubel E, Vaessen T, Steinhart H, Klippel A, Lafit G, et al. Efficacy of Acceptance and Commitment Therapy in Daily Life in Early Psychosis: Results from the Multi-Center INTERACT Randomized Controlled Trial. *Psychother Psychosom* 2022; **91**(6): 411-23.

28. Nuechterlein KH, Ventura J, Subotnik KL, Gretchen-Doorly D, Turner LR, Casaus LR, et al. A randomized controlled trial of cognitive remediation and long-acting injectable risperidone after a first episode of schizophrenia: improving cognition and work/school functioning. *Psychol Med* 2022; **52**(8): 1517-26.

29. Palma C, Farriols N, Frias A, Canete J, Gomis O, Fernandez M, et al. Randomized controlled trial of cognitive-motivational therapy program (PIPE) for the initial phase of schizophrenia: Maintenance of efficacy at 5-year follow up(✰). *Psychiatry Res* 2019; **273**: 586-94.

30. Penn DL, Uzenoff SR, Perkins D, Mueser KT, Hamer R, Waldheter E, et al. A pilot investigation of the Graduated Recovery Intervention Program (GRIP) for first episode psychosis. *Schizophr Res* 2011; **125**(2-3): 247-56.

31. Pos K, Franke N, Smit F, Wijnen BFM, Staring ABP, Van der Gaag M, et al. Cognitive behavioral therapy for social activation in recent-onset psychosis: Randomized controlled trial. *J Consult Clin Psychol* 2019; **87**(2): 151-60.

32. Robinson DG, Schooler NR, Marcy P, Gibbons RD, Hendricks Brown C, John M, et al. Outcomes During and After Early Intervention Services for First-Episode Psychosis: Results Over 5 Years From the RAISE-ETP Site-Randomized Trial. *Schizophr Bull* 2022; **48**(5): 1021-31.

33. Rocha NB, Campos C, Figueiredo JM, Saraiva S, Almeida C, Moreira C, et al. Social cognition and interaction training for recent-onset schizophrenia: A preliminary randomized trial. *Early Interv Psychiatry* 2021; **15**(1): 206-12.

34. Ruggeri M, Bonetto C, Lasalvia A, Fioritti A, de Girolamo G, Santonastaso P, et al. Feasibility and Effectiveness of a Multi-Element Psychosocial Intervention for First-Episode Psychosis: Results From the Cluster-Randomized Controlled GET UP PIANO Trial in a Catchment Area of 10 Million Inhabitants. *Schizophr Bull* 2015; **41**(5): 1192-203.

35. Sonmez N, Romm KL, Ostefjells T, Grande M, Jensen LH, Hummelen B, et al. Cognitive behavior therapy in early psychosis with a focus on depression and low self-esteem: A randomized controlled trial. *Compr Psychiatry* 2020; **97**: 152157.

36. Srihari VH, Tek C, Kucukgoncu S, Phutane VH, Breitborde NJ, Pollard J, et al. First-Episode Services for Psychotic Disorders in the U.S. Public Sector: A Pragmatic Randomized Controlled Trial. *Psychiatr Serv* 2015; **66**(7): 705-12.

37. Uzenoff SR, Perkins DO, Hamer RM, Wiesen CA, Penn DL. A preliminary trial of adherence-coping-education (ACE) therapy for early psychosis. *J Nerv Ment Dis* 2008; **196**(7): 572-5.

38. Valencia M, Juarez F, Ortega H. Integrated treatment to achieve functional recovery for first-episode psychosis. *Schizophr Res Treatment* 2012; **2012**: 962371.

39. van Duin D, de Winter L, Kroon H, Veling W, van Weeghel J. Effects of IPS plus cognitive remediation in early psychosis: 18-month functioning outcomes of a randomized controlled trial. *Schizophr Res* 2021; **236**: 115-22.

40. Vidarsdottir OG, Roberts DL, Twamley EW, Gudmundsdottir B, Sigurdsson E, Magnusdottir BB. Integrative cognitive remediation for early psychosis: Results from a randomized controlled trial. *Psychiatry Res* 2019; **273**: 690-8.

41. Chinn S. A simple method for converting an odds ratio to effect size for use in meta-analysis. *Stat Med* 2000; **19**(22): 3127-31.

42. Nikolakopoulou A, Higgins JPT, Papakonstantinou T, Chaimani A, Del Giovane C, Egger M, et al. CINeMA: An approach for assessing confidence in the results of a network meta-analysis. *PLoS Med* 2020; **17**(4): e1003082.

43. Austin SF, Mors O, Secher RG, Hjorthoj CR, Albert N, Bertelsen M, et al. Predictors of recovery in first episode psychosis: the OPUS cohort at 10 year follow-up. *Schizophr Res* 2013; **150**(1): 163-8.

44. Diaz-Caneja CM, Pina-Camacho L, Rodriguez-Quiroga A, Fraguas D, Parellada M, Arango C. Predictors of outcome in early-onset psychosis: a systematic review. *NPJ Schizophr* 2015; **1**: 14005.
